# The longitudinal course of childhood bullying victimization and associations with self‐injurious thoughts and behaviors in children and young people: A systematic review of the literature

**DOI:** 10.1002/jad.12097

**Published:** 2022-10-09

**Authors:** Emma Wilson, Holly Crudgington, Craig Morgan, Colette Hirsch, Matthew Prina, Charlotte Gayer‐Anderson

**Affiliations:** ^1^ Department of Health Service & Population Research Institute of Psychiatry, Psychology & Neuroscience, King's College London London UK; ^2^ ESRC Centre for Society and Mental Health, King's College London London UK; ^3^ Department of Psychology Institute of Psychiatry, Psychology & Neuroscience, King's College London London UK; ^4^ South London and Maudsley NHS Foundation Trust, Bethlem Royal Hospital Kent UK

**Keywords:** bullying, gender, review, self‐harm, suicide, youth

## Abstract

**Introduction:**

Bullying victimization has consistently been highlighted as a risk factor for self‐injurious thoughts and behaviors (SITBs) in young people. This systematic review of prospective, community‐based studies explored associations between bullying victimization (traditional/face‐to‐face and cyber) across the full spectrum of self‐harm and suicidality, in children and young people aged up to (and including) 25 years. Importantly, associations by sex/gender were explored.

**Methods:**

MEDLINE, Embase, PsycINFO, CINAHL and Scopus were searched for articles meeting the inclusion criteria. Articles were screened by title, abstract and full text. Quality appraisal was performed using the Newcastle‐Ottawa Scale for cohort studies. Data were synthesized narratively. The protocol is registered on PROSPERO (CRD42021261916) and followed PRISMA 2020 guidelines.

**Results:**

A total of 35 papers were included, across 17 countries. Results were presented by bullying type: traditional/face‐to‐face (*n* = 25), cyber (*n* = 7) and/or an aggregate of both types (*n* = 7). Outcomes included suicidal ideation (*n* = 17), self‐harm (*n* = 10), suicide attempt (*n* = 4), NSSI (*n* = 4), other (*n* = 7). Studies measured outcomes in under 18s (*n* = 24), 18–25‐year‐olds (*n* = 8) and both under 18s and 18–25‐year‐olds (*n* = 3). Studies exploring the role of sex/gender (20%) found some interesting nuances.

**Conclusions:**

Some weak to strong associations between bullying and SITBs were found yet conclusions are tentative due to study heterogeneity (e.g., methods used, conceptualizations and operationalisations of exposures/outcomes). Future research should address methodological issues raised in this review, and further explore gender differences in bullying, including by bullying sub‐types (e.g., overt or relational) and victim status (e.g., victim or bully‐victim).

## 1 INTRODUCTION

Traditional, face‐to‐face bullying is described as aggressive, intentional actions carried out by one or more persons repeatedly and over time against a victim who cannot easily defend themselves (Olweus, 1994), and this definition remains widely accepted today (Kwan et al., 2020). Traditional forms of bullying include direct, face‐to‐face forms of physical and verbal attacks, such as hitting, kicking, punching, threats, taunts and insults (Smith et al., 2013), and/or indirect forms, such as intentional exclusion from activities and friendship groups or spreading rumors that aim to tarnish a person's reputation (Björkqvist et al., 1992; Olweus, 1994). Overall, there is consensus that bullying victimization (“bullying” from here on) is repetitive, involves intent to harm by the aggressor, and involves a power imbalance between the bully and victim (Farrington, 1993; Olweus, 1994, 2010; Smith & Brain, 2000; Younan, 2019). This power imbalance could be through physical strength, the size of their group, or being more popular (Hunter et al., 2007).

The field now includes cyberbullying, where technology is a tool for targeting victims (Smith et al., 2008). Although many definitions of cyberbullying are based on the original criteria of traditional bullying (Englander et al., 2017), cyberbullying has its own nuances, particularly the operationalisation of “repetition” and “power imbalance” (Salmon et al., 2018). For example, one message on social media may be retweeted by many people, to a far wider audience, potentially all behind a guise of anonymity. Despite this, some researchers have agreed that the key criteria for bullying are, for the most part, applicable to cyberbullying, and that it is another form of bullying, alongside verbal, physical and relational (Olweus & Limber, 2018; Smith et al., 2013).

Bullying is a form of peer victimization, which refers to instances where a child is frequently targeted by peer aggression, such as at school (Hunter et al., 2007; Kochenderfer & Ladd, 1996). Although bullying and peer victimization both involve recurring aggressive acts with negative effects, bullying is a particular form of peer victimization that may be viewed as more serious due to the requirement of both a power imbalance and intention to harm, which is not required for the definition of peer victimization (Hunter et al., 2007; Menin et al., 2021). Bullying is also distinct from other forms of victimization, such as child maltreatment, assault, sexual abuse and family violence, although these may occur alongside bullying to describe instances of “polyvictimisation” (Finkelhor et al., 2011). Importantly, bullying is differentiated from other forms of abuse by the context in which it occurs and the people involved; for example, where the aggressor is a classmate rather than a parent, older sibling or romantic partner (Skrzypiec et al., 2018). The negative repercussions from childhood bullying have long been studied, ranging from the development of mental health conditions to poor adjustment in adulthood (Arseneault, 2017; Moore et al., 2017). It has consistently been reported as a risk factor for self‐harm and suicidal behavior, which is particularly salient for young people (Hinduja & Patchin, 2010; Holt et al., 2015; Witt et al., 2019).

Self‐harm, defined as self‐inflicted injury or poisoning, irrespective of suicidal intent, is one of the strongest risk factors for future suicide, where suicide is 30 times more likely in those with a history of self‐harm (Hawton et al., 2012, 2020). Among young people, rates of self‐harm have been rising globally in primary care and hospital settings (Cairns et al., 2019; Griffin et al., 2018; Morgan et al., 2017), and it is anticipated that self‐harm is even higher in the community (Geulayov et al., 2018). Suicide is the second largest cause of death in 10–34‐year‐olds in the United States (Centers for Disease Control and Prevention, 2020) and the leading cause of death in under 19s in the United Kingdom (Office for National Statistics, 2019). Despite being separate entities with differences in frequency, intention and fatality, self‐harm and suicidality (i.e., suicidal thoughts and behaviors) may be conceptualized as existing on a spectrum of self‐injurious thoughts and behaviors (SITBs), due to shared risk factors and behaviors that co‐exist (Hamza et al., 2012; Nock et al., 2006).

The relationship between self‐harm and suicide can also be understood from a theoretical perspective. Based on the interpersonal theory of suicidal behavior (Joiner, 2007), suicide attempts require two components: the desire to die (perceived burdensomeness and a sense of low belongingness) and the capacity to do so, also called acquired capability for suicide. It is thought that previous self‐injury may increase perceived capability for suicide through repeated exposure to physical pain, contributing towards the development of fearlessness (Van Orden et al., 2010). However, for many people, self‐harm may exist at the other end of the SITB spectrum as a maladaptive coping strategy that is not driven by suicidal intent, but serves to manage negative affect and communicate distress arising from intra‐ and interpersonal difficulties such as bullying (Nock et al., 2009; Plener et al., 2018).

**Figure 1:**
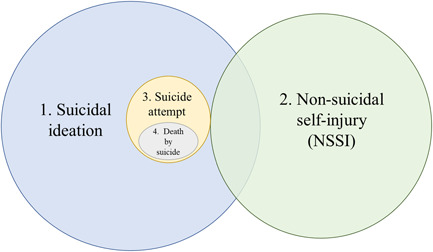
Conceptualization of self‐harm and suicidality in North America [Color figure can be viewed at wileyonlinelibrary.com]

**Figure 2:**
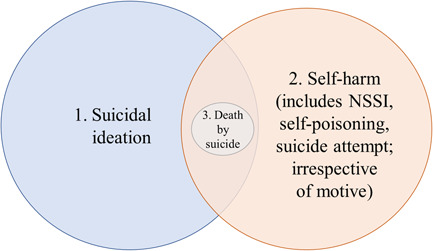
Conceptualization of self‐harm and suicidality in United Kingdom [Color figure can be viewed at wileyonlinelibrary.com]

Definitions of self‐harm and suicide vary, and previous reviews looking at the association between bullying and/or peer victimization have tended to focus on one or two aspects rather than the whole spectrum; such as nonsuicidal self‐injury (NSSI), suicidal ideation, or suicide attempt (Heerde & Hemphill, 2019; Holt et al., 2015; Serafini et al., 2021). While North America tends to separate NSSI from suicide attempts (see Figure 1), countries such as the United Kingdom consider the broader construct of self‐harm (see Figure 2), disregarding motivation, which is seen as too fluid a construct to form the basis of a clinical decision (Kapur et al., 2013). The merits and drawbacks of both approaches is discussed elsewhere (Kapur et al., 2013; Wilson & Ougrin, 2021). This review aims to provide a comprehensive overview across the spectrum of SITBs, bringing together studies from different countries, while retaining the differences in terminology based on the authors' original definitions.

This systematic review aims to build on previous work in several ways: First, by focusing solely on the prospective, longitudinal effects to better understand the temporal nature of the association between bullying and suicidal thoughts and behaviors (Holt et al., 2015), particularly where studies have adjusted for associated covariates. Second, by looking at community‐based studies, as many cases remain undetected in the community, particularly self‐harm (Geulayov et al., 2018; Hawton et al., 2012). Third, by focusing on studies that follow young people up to and including the age of 25, which meets the definition^1^ of the World Health Organization (2021), and given that development is thought to continue until this age (Sawyer et al., 2018). This is also in line with previous reviews (Abdelraheem et al., 2019; John et al., 2018; Williams et al., 2021). Fourth, by considering the broad spectrum of SITBs, and the specific construct of bullying victimization, rather than peer victimization more broadly. Finally, by considering whether the associations differ by sex/gender. As such, the overall aim is to summarize the longitudinal course of childhood bullying victimization and associations with self‐harm, suicidal ideation and suicidal behaviors in children and young people, across nonclinical settings.

Objectives:

1. Is bullying victimization in childhood and adolescence associated with future self‐harm, suicidal thoughts and suicidal behaviors in children and young people up to, and including, the age of 25?
2. Do these associations differ for type of bullying victimization (i.e., traditional bullying and/or cyberbullying victimization)?
3. Do these associations differ by sex/gender?

## 2 METHODS

### 2.1 Protocol and registration

This review follows the recommendations of Preferred Reporting Items for Systematic Reviews and Meta‐Analyses (PRISMA) 2020 guidelines (see Materials S1 for PRISMA checklist). The protocol was pre‐registered on PROSPERO (CRD42021261916).

### 2.2 Inclusion and exclusion criteria

Studies were required to meet the following inclusion criteria: (1) original, empirical research published in a peer reviewed journal; (2) examines the relationship between exposure to bullying victimization as a child or adolescent under 18 years old, and the outcome of self‐harm or suicidal ideation or behavior as a child or young adult under 26 years old; (3) uses a longitudinal, prospective design with a minimum of two time points; (4) community‐based studies; (5) written in English; (6) has a comparator (i.e., a group of bullied vs. nonbullied children). The main outcome was any form of self‐harm (NSSI, self‐poisoning, self‐injury) or suicidal thoughts or behaviors (ideation, attempts). This study did not examine a particular type of bullying victimization; therefore, all direct and indirect forms of bullying, including cyberbullying, were included. Similar to the approach of Holt et al. (2015), studies described by the authors as measuring peer victimization and aggression more generally were not included. Studies were excluded if they only looked at bullying perpetration, and clinical samples were excluded to focus on understanding self‐harm and suicidality in the community. Cross‐sectional studies, case series, case reports, qualitative studies, opinion pieces, editorials, reviews, meta‐analyses and intervention studies were not included.

### 2.3 Search strategy

An electronic search of the following databases was run on July 6, 2021, limited to studies in the English language: MEDLINE (OVID), EMBASE (Ovid), PsycINFO (Ovid), CINAHL and Scopus (Elsevier). A search string was developed to include relevant keywords when searching title and abstracts with subject heading searching where possible (see Materials S2).

Additionally, a manual search of reference lists from relevant published systematic reviews was conducted. Web of Science was used to undertake forward and backwards citation searching of reference lists from included studies. Based on good practice guidance issued by PROSPERO, searches were re‐run just before the final analyses and any further studies identified and retrieved for inclusion.

Citations were imported into EndNote and duplicates removed, before being uploaded onto Raayan (Ouzzani et al., 2016). Two researchers (EW and HC) independently reviewed 10% percent (*n* = 62) of title and abstracts, and agreement checked, before both screened the remaining 90% (*n* = 556) based on the inclusion criteria. The same process was repeated when reviewing the 78 papers at the full text stage. A third researcher CGA made the final decision if consensus was not met. The search was re‐run on April 15, 2022 and due to high agreement in the first screening (>90%), EW independently conducted the updated search and consulted with HC for any papers causing uncertainty.

### 2.4 Data extraction

Data from eligible studies was extracted into a predesigned form in Microsoft Excel based on predetermined criteria: key study details (author, year, country), setting (e.g., urban/rural), study design and duration of follow up period (months or years, waves), sample characteristics (baseline sample size, final sample size, sex/gender, age, attrition rate), details about exposure (traditional, cyber or aggregate, or sub‐types such as physical or relational bullying; measurement/scale used), details about outcome (NSSI, self‐harm, suicidal ideation, suicide attempt or other; measurement/scale used), variables adjusted for/covariates, statistical analyses used (e.g., odds ratio [OR], risk ratio [RR]) and relevant results, including results stratified by sex/gender. Authors were contacted if key information could not be ascertained from the paper, its Supporting Information Materials or a previous paper referenced in the article that lists more details of the sample characteristics and study procedure.

### 2.5 Quality assessment

Each study was subject to quality assessment using the Newcastle‐Ottawa Quality Assessment Scale for cohort studies (NOS; Wells et al., 2014), with grading in the following categories: (1) selection of cohorts, including representativeness and ascertainment of bullying status; (2) comparability of cohorts, and the use of appropriate methods to control for confounding; (3) assessment of outcome, including adequacy of follow up (see Materials S3 for scoring sheet). Specifically, a follow up of 6 months and response rate of 80%, with an adequate description of participants lost to follow up, was deemed appropriate based on previous relevant reviews using NOS for longitudinal studies (Moore et al., 2017; Valencia‐Agudo et al., 2018). The quality score for ascertainment of exposure and outcome was awarded when using secure records (e.g., medical records), structured interview or self‐report questionnaires with validated measurements (Latham et al., 2021; Moore et al., 2017). Using a star grading system based on thresholds used in other reviews (Polihronis et al., 2022; Williams et al., 2021), studies received an overall quality score of low (0–3 stars), medium (4–6 stars) or high (7–9 stars).

### 2.6 Data analysis

To aid comparability, studies were analysed and results reported based on exposure type: traditional bullying victimization only, cyberbullying victimization only, bullying victimization (all types). Papers could appear in more than one group if they separated results by type of bullying (e.g., presenting results for traditional bullying separately to cyberbullying). Results were categorized as measuring traditional bullying if: (1) this was explicitly stated (e.g., they measure face‐to‐face overt, physical or relational bullying but not cyberbullying); (2) if the data was collected before the 2000s (as cyberbullying is a modern concept); or (3) if the validated scale or items did not explicitly refer to electronic bullying (i.e., they were developed to measure traditional bullying; Smith et al., 2008). Results for cyberbullying included studies that gave results for this specific type of bullying and the association with the outcomes. Results were classified as “bullying victimization (all types)” if the study used an aggregate measure (i.e., grouping traditional and cyberbullying together), or if their methods section was too vague to ascertain how bullying was measured.

Additionally, supplementary analyses assessed the measures used to capture bullying; specifically, whether a definition was provided to participants and whether the measure captured the three components of bullying (i.e., power imbalance, repetition, intention to cause harm). Authors were contacted if this information could not be ascertained from the manuscript.

A meta‐analysis was not performed due to heterogeneity between studies in the exposure and outcomes assessed, and the measures used.

## 3 RESULTS

### 3.1 Study selection

**Figure 3:**
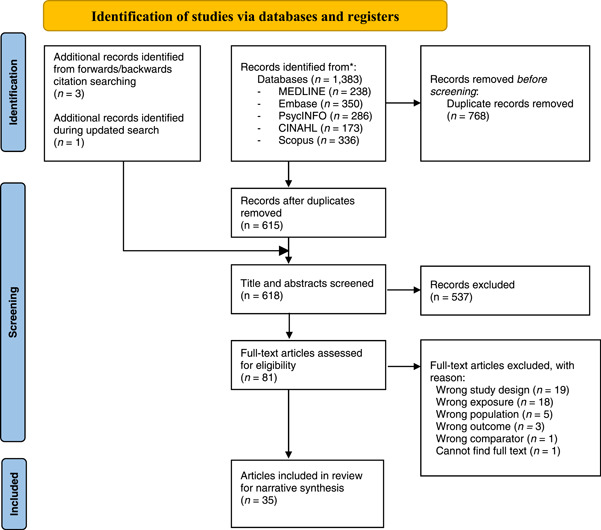
PRISMA flow chart depicting study selection process. PRISMA, Preferred Reporting Items for Systematic Reviews and Meta‐Analyses [Color figure can be viewed at wileyonlinelibrary.com]

A total of 1383 records were identified through searching five academic databases. An additional three articles were identified through searching the reference list of previous relevant systematic reviews, and through forwards/backwards citation searching of articles included for the current review by using Web of Science. After 768 duplicates were removed, the title and abstracts of 61 studies were screened, resulting in 81 studies for full text review. Articles were excluded if they included the wrong age group (*n* = 5), did not use a prospective methodology or community‐based sample (*n* = 19), did not have a suitable comparator to explore the association between exposure and outcome (e.g., if all the cohort are victims of bullying, *n* = 1), measured the wrong exposure (e.g., sexual victimization or peer victimization rather than bullying victimization, *n* = 18) or the wrong outcome (*n* = 3). A total of 35 articles were included in the final review for qualitative synthesis. Figure 1 details the process in a PRISMA flow chart (Figure 3).

### 3.2 Research design of studies

A description of the 35 articles is presented in Table 1 and Materials S4. Of these 35 articles, there were 27 unique cohort/follow‐up studies (i.e., 15 articles used data from a cohort/follow‐up study that is also used in another article; 20 articles used data from a cohort/follow‐up study not used in another included article). Four articles used data from the Avon Longitudinal Study of Parents and Children (ALSPAC) and two articles for each of the following: the Epidemiological Multicenter Child Psychiatric Study in Finland, Great Smoky Mountains Study (GSMS), Korea Welfare Panel Study (KOWEPS), Leuven College Surveys (LCS), Youth and Mental Health Study, a 6‐month follow‐up study of Vietnamese students. The studies represented 17 countries: 5 articles were from the United Kingdom; 4 from Finland; 3 from Korea; 2 each from Australia, Belgium, Canada, China, Norway, United States, Vietnam; 1 each from New Zealand, Israel, Spain, Sweden, Switzerland, Taiwan, The Netherlands. Additionally, there was one study of 10 European countries (Brunstein Klomek et al., 2019), and one study that looked at two samples, one from the United States and one from the United Kingdom (Lereya et al., 2015). All studies were longitudinal and studies had a minimum of two waves of data collection, with overall study duration ranging from 4 months (Quintana‐Orts et al., 2022) to 17 years (Copeland et al., 2013; Lereya et al., 2015). Most studies used univariable (e.g., logistic regression) and multivariable (e.g., multiple logistic regression) inferential statistics, presenting unadjusted and adjusted results (e.g., ORs, RRs). Variables commonly controlled or adjusted for include sex/gender, age, socioeconomic status, baseline mental health including the outcome of interest. Some studies used structural equation models, cross‐lagged panel analysis and/or path analysis instead of, or in addition to, logistic/linear regression models (Brunstein Klomek et al., 2019; Cho & Glassner, 2020; Cho, 2019; Garisch & Wilson, 2015; Le et al., 2019; Lereya et al., 2013; Lung et al., 2020; Zhu et al., 2021).

**Table 1:** Summary of study characteristics

Summary of study characteristics included in the systematic review					
Author(s), year, study name (acronym), country		Bullying victimization: type, assessment, timeframe	Outcome, assessment of outcome (e.g., collection methods and scale)	Analysis used, measure of effect	Adjusted for/covariates
Bannink et al. (2014) Rotterdam Youth Monitor (RYM) The Netherlands		1. Traditional bullying victimization and 2. Cyberbullying victimization Own measure, self‐report questionnaire (in class), past 4 weeks	Suicidal ideation Own measure, self‐report questionnaire (in class), past 12 months	Binary logistic regression, ORs with 95% CIs	Bullying victimization (cyber and traditional), mental health problems, suicidal ideation Model 1 is adjusted for sociodemographic characteristics (i.e., gender, age, ethnicity, education) and BV. Model 2 is adjusted for Model 1 + suicidal ideation at baseline. Model 3a is adjusted for Model 2 + also includes a Gender × Traditional Bullying Victimization interaction term. Model 3b is adjusted for Model 2 + also includes a Gender × Cyberbullying Victimization interaction term
Benatov et al. (2022) Israel		1. Traditional bullying victimization and 2. Cyberbullying victimization Measure used in Klomek et al. (2015), self‐report questionnaire (in class), past 6 months	1. Suicidal ideation 2. Suicide attempts Paykel Suicide Scale (PSS; Paykel et al., 1974), self‐report questionnaire (in class), past 2 weeks	Logistic regression, ORs with 95% CI	Model 1 controlling for suicide ideation/attempts at Time 1. Model 2 additionally controlling for depressive symptoms at Time 1. Model 3 additionally controlling for hostility at Time 1. Model 4 additionally controlling for traditional bullying perpetration
Blasco et al. (2019) UNIVERSAL (University and Mental Health) Spain		Bullying victimization Bully Survey (Swearer & Cary, 2003), self‐report questionnaire (online), before age of 17	Suicidal ideation Self‐Injurious Thoughts and Behaviors Interview (SITBI; Nock et al., 2007), and screening version of the Columbia‐Suicide Severity Rating Scale (C‐SSRS; Posner et al., 2007), self‐report questionnaire (online), past 12 months	Multiple logistic regression, ORs with 95% CIs	Multivariable models adjusted by: Age, gender, university, academic field, country of birth, parents' studies and living location; baseline suicidal ideation (SI) for analyses looking at first‐onset of SI
Borschmann et al. (2020) Childhood to Adolescence Transition Study (CATS) Australia		Bullying victimization Gatehouse Bullying Scale (Bond et al., 2007) and own measure about cyberbullying, self‐report questionnaire, timeframe NR	Self‐harm Own measure, self‐report questionnaire, past 12 months	Multivariable logistic regression within generalized estimating equations framework, ORs with 95% CIs	Adjusted for age (in years, centered around 12.0 years), sex, and Socioeconomic Index For Areas (SEIFA) advantage/disadvantage quintile
Brunstein‐Klomek et al. (2019) Saving and Empowering Young Lives in Europe (SEYLE) 10 European countries		1. Physical BV 2. Verbal BV 3. Relational BV 4. Any BV Global School‐Based Student Health Survey (GSHS; World Health Organization, 2009), self‐report questionnaire (in class), past 3/12 months	1. Suicidal ideation and 2. Suicide attempt Paykel Hierarchical Suicidal Ladder (Paykel et al., 1974), T2 (past 3 months), self‐report questionnaire (in class), T3 (past 12 months)	Multilevel autoregressive cross‐lagged models, ORs with beta values	Gender, age, whether the adolescent was living without his biological parents (yes, no), whether the adolescent is an immigrant (yes, no), and whether the adolescent's parents lost their job during the last 12 months (yes, no) were included as covariates to account for their effects (Wasserman et al., 2015). In the models predicting suicide ideation and/or suicide attempts, depression included as a covariate.
Cho (2019) Korea Welfare Panel Study (KOWEPS) Korea		Bullying victimization Own measure, household survey collected through interviews, past year	Suicidal ideation Scale of Suicide Ideation (Beck et al., 1979), Wave 7 interview, current	Latent class regression model, β with SE, ORs	Model 2: mediating negative emotions
Cho and Glassner (2020) Korea Welfare Panel Study (KOWEPS) Korea		Bullying victimization Own measure, household survey collected through interviews, past year	Suicidal ideation Scale of Suicide Ideation (Beck & Beck, 1972), Wave 7 interview, current	Longitudinal mediation analysis (causal steps), coefficient analysis, beta values and SE	Grade, SES, negative emotions (causal steps analysis)
Copeland et al. (2013) Great Smoky Mountains Study (GSMS) USA		Bullying victimization Child and Adolescent Psychiatric Assessment (CAPA; Angold & Costello, 2000), child and parent interview, past 3 months	Suicidality (ideation, plans, attempt) DSM‐IV criteria for Major Depressive Episode (APA; 1994), as part of Young Adult Psychiatric Assessment interviews (Angold & Costello, 2000), past 3 months	Weighted logistic regressions, ORs with 95% CIs	Childhood psychiatric status, family hardships
Fisher et al. (2012) Environmental Risk (E‐Risk) Study UK		Bullying victimization Own measure previously used with good test‐retest reliability, structured interview with mother at ages 7 and 10, interview with child at age 12, timeframe NR	Self‐harm Own measure, interview with mother when child was age 12, past 6 months	Modified Poisson regression, RRs with robust 95% CIs	Physical maltreatment by adults, internalizing and externalizing problems at age 5, IQ at age 5
Garisch and Wilson (2015) New Zealand		Bullying victimization Six items from Peer Relations Questionnaire (Rigby & Slee, 1995), and additional question on cyberbullying, self‐report questionnaire (at school), timeframe NR	NSSI Deliberate Self‐Harm Inventory—Short form (Lundh et al., 2007), self‐report questionnaire (at school), past 3–8 months	Cross‐lagged panel correlations, Pearson's correlations and *p* value (=<0.10)	Anxiety & depressive symptoms; self‐esteem; alexithymia; adaptive emotional response; resilience; impulsivity; physical & sexual abuse history; substance abuse; sexuality concerns; mindfulness
Geoffroy et al. (2021) National Longitudinal Survey of Children and Youth (NLSCY) Canada		Bullying victimization Own measure, self‐report questionnaire, current	Suicide attempt Own measure, self‐report questionnaire, past 12 months	Univariable and multinomial logistic regressions, RR with 95% CI and *p* value	Univariable adjusted for sex Multivariable associations: 10 risk factors plus sex
Heikkilä et al. (2013) Adolescent Mental Health Cohort Study (AMHCS) Finland		Bullying victimization 2 questions from the WHO Youth Health Study (King et al., 1996), self‐report questionnaire (in class), previous semester	Suicide ideation One item from the R‐BDI (Raitasalo, 2007), a Finnish modification of the 13‐item Beck Depression Inventory (1972), self‐report questionnaire (post/online), current	Logistic regression, ORs with 95% CIs	Model 1: involvement in bullying at age 15 was entered after controlling for sex and age. Model 2: depressive symptoms at age 15 were added. Model 3: depressive symptoms removed and externalizing symptoms at age 15 added. Model 4: both depressive and externalizing symptoms at age 15 are included.
Hemphill et al. (2015) International Youth Development Study (IYDS) Australia		1. Cybervictimisation 2. Traditional victimization CV: own measure, TBV: Gatehouse Bullying Scale (Bond et al., 2000, 2007) self‐report questionnaire (in class or telephone), timeframe NR	Self‐harm Own measure, self‐report questionnaire (in class or telephone), past year	Logistic regression, ORs with 95% CIs	In adjusted models: traditional bullying, individual risk factors (impulsivity, concentration), peer risk factors, family risk factors, school risk factors. All analyses controlled for gender and clustering of schools.
Kiekens et al. (2019) Leuven College Surveys Belgium		Bullying victimization Bully Survey (Swearer & Cary, 2003), self‐report questionnaire, before age of 17	NSSI Self‐report version of the Self‐Injurious Thoughts and Behaviors Interview (Nock et al., 2007), self‐report questionnaire, past 12 months	Logistic regression, ORs with 95% CIs	Multivariate model adjusted for parental psychopathology, physical abuse, emotional abuse, sexual abuse, neglect, dating violence
Kim et al. (2009­) South Korea		Bullying victimization Korean Peer Nomination Inventory (K‐PNI; Kim et al., 2001), self‐report questionnaire (in class), current (peer nomination)	1. Suicidal behaviors 2. Suicidal ideations (a. >6 months, b. >2 weeks) Two items from the Korean Youth Self‐Report (K‐YSR; Oh et al., 1997) and adapted measure for acute suicidal ideation, self‐report questionnaire (in class), past 6 months and past 2 weeks	Multivariate logistic regression, ORs with 95% CIs	(1) socio‐demographic risk factors: sex, family structure, parental educational level and SES; (2) psychopathological risk factors at baseline: anxious/factors at baseline: depression, conduct problems and aggression; and, (3) past suicide history (suicidality at baseline).
Klomek et al. (2008)		Bullying victimization Own measure, self‐report questionnaire (student: in class, parent: by post), past month	Suicidal ideation Item 9 of the Beck Depression Inventory (BDI; Beck et al., 1961), self‐report questionnaire (at military call up), past 6 months	Logistic regression, ORs with 95% CIs	Depression at age 8
Klomek et al. (2009) Epidemiological Multicenter Child Psychiatric Study (EMCPS) in Finland Finland		Bullying victimization Own measure, self‐report questionnaire with student and parent at age 8, timeframe NR	Suicide attempt or completed suicide Finnish Hospital Discharge Register, up to age 25	Logistic regression, ORs with 95% CIs	Baseline conduct symptoms (based on the parent's Rutter conduct scale) and/or baseline depression (based on the CDI).
Le et al. (2017) Vietnam		Bullying victimization Revised Olweus Bully/Victim Questionnaire (Olweus, 1996), self‐report questionnaire (in class), past 6 months	Suicidal ideation Three items adapted from the American School Health Association report (1989), self‐report questionnaire, past 6 months	Binary logistic regression, coefficient and ORs with 95% CIs	Adjusted models controlled for confounders measured at Time 1 including: age, depression (for model of depression), psychological distress (for model of psychological distress), suicidal ideation (for model of suicidal ideation), self‐esteem, average time spending on online, family social support, school social support, friend social support, witness parental violence, and conflict with siblings
Le et al. (2019) Vietnam		Bullying victimization Revised Olweus Bully/Victim Questionnaire (Olweus, 1996), self‐report questionnaire (in class), past 6 months	Suicidal ideation Three items adapted from the American School Health Association report (1989), self‐report questionnaire, past 6 months	Cross‐lagged panel analysis, RRs, ORs and coefficients.	Demographics (gender, age in years, family structure), family, friend, and school social support (perception of students and teachers helping to stop bullying), witness parental violence, conflict with siblings, time spent on online, previous suicidal ideation
Lereya et al. (2013) Avon Longitudinal Study of Parents and Children (ALSPAC) UK		Bullying victimization Bullying and Friendship Interview Schedule (detailed in Wolke et al., 2012), child interview (8 and 10 years), past 6 months; mother interview (child aged 7, 8, 9 years), lifetime; teacher interview (child aged 7 and 10 years), lifetime	Self‐harm Own measure, self‐report questionnaire when child aged 16–17 years, past year	1. Binary logistic regression, OR and 95% CIs 2. Path analysis (The rootmean square error of approximation (RMSEA) and the Comparative Fit Index (CFI)), Probit coefficients (categorical observed variables), with SE and two‐tailed *p* value	Sex, preschool maladaptive parenting, preschool domestic violence, internalizing/externalizing behavior, BPD symptoms, depression symptoms
Lereya et al. (2015) Avon Longitudinal Study of Parents and Children (ALSPAC), UK Great Smoky Mountains Study (GSMS), USA		Bullying victimization ALSPAC: Bullying and Friendship Interview Schedule (Woods & Wolke, 2003), child interview at age 8, 10, and 13, timeframe NR GSMS: Child and Adolescent Psychiatric Assessment (CAPA; Angold & Costello, 2000), child and parent interview, past 3 months	Self‐harm and suicidality Self‐harm: Standard clinical interviews for self‐harm (CIS‐R; Lewis et al., 1992), interview with young person, past year Suicidality (recurrent thoughts of wanting to die, recurrent suicidal ideation without a specific plan, suicidal plans or a suicide attempt): Young Adult Psychiatric Assessment (YAPA; Angold & Costello, 2000), timeframe NR	Binary logistic regressions, OR and 95% CIs	For ALSPAC: adjusted for sex, family adversity during pregnancy and any prenatal maternal mental health problems (anxiety and/or depression) For GSMS: adjusted for sex, socioeconomic status, family instability and family dysfunction; and percentages are weighted; sample sizes are unweighted
Lung et al. (2020) Taiwan Birth Cohort Pilot Study (TBCS‐P) Taiwan		Bullying victimization Own measure, self‐report questionnaire, lifetime	Deliberate self‐harm Own measure, self‐report questionnaire, past year	SEM, β coefficient and *p* values	None
Mars et al. (2020) Avon Longitudinal Study of Parents and Children (ALSPAC) UK		Cyberbullying victimization Own measure, self‐report questionnaire (online), lifetime	Self‐harm Own measure, self‐report questionnaire (post/online), past year	Multiple logistic regression, ORs, Adjusted ORs with 95% CIs	Socioeconomic position (SEP), previous mental health problems, total numbers of hours spent online
Mortier et al. (2017) Leuven College Surveys (LCS) Belgium		Bullying victimization The Bully Survey (Swearer & Cary, 2003), self‐report questionnaire (online), before age of 17	Suicidal thoughts and behaviors (STB): 1. Suicidal ideation, 2. Suicidal plan Items on suicidal thoughts and behaviors taken from the Self‐Injurious Thoughts and Behaviors Interview (Nock et al., 2007) self‐report questionnaire (online), past year	1. Logistic regression, ORs, Adjusted ORs with 95% CIs 2. Multivariate prediction model, predicted probabilities (reported as OR 95% CI and PARP)	Analyses only included those without history of STB at baseline. All analyses were adjusted for sociodemographics (i.e., gender, age, nationality, familial composition and socioeconomic status, sexual orientation, university group membership, and living situation), and all other risk factors shown in the table (i.e., abuse, neglect, dating violence, 12‐month mental disorders, 12‐month stressful experiences)
O'Connor et al. (2009) Child and Adolescent Self‐harm in Europe survey (CASE) UK		Bullying victimization Own measure, self‐report questionnaire (in class), lifetime	Deliberate self‐harm Own measure, self‐report questionnaire (in class), between baseline and T2 (6 months)	Univariate logistic regression, ORs with 95% CIs	None
Özdemir and Stattin (2011) Swedish Seven Schools Longitudinal Study (SSSLS) Sweden		Bullying victimization A measure originally developed for a survey of bullying in Switzerland and Norway (Alsaker & Brunner, 1999), self‐report questionnaire, last semester	Deliberate self‐harm Nine‐item version of Deliberate Self‐Harm Inventory (DSHI‐9), a revised version of the original DSHI (Gratz, 2001), which has been adapted to adolescents (Lundh et al., 2007), self‐report questionnaire, past 6 months	Ordinary least squares (OLS) regression models with hierarchical entry, β coefficient and *p* values	Age, gender
Perret et al. (2020) Quebec Longitudinal Study of Child Development (QLSCD) Canada		1. Face‐to‐face (traditional) bullying victimization 2. Cybervictimisation Modified version of the Self‐Report Victimization Scale (Ladd & Kochenderfer‐Ladd, 2002), self‐report questionnaire, since start of school year	Suicidal ideation/attempt Own measure, self‐report questionnaire, past 12 months	Logistic regression, ORs, Adjusted ORs with 95% CIs	Model 1 adjusted for sex. Model 2 additionally adjusted for prior family socioeconomic status (6–12 years), family structure (12 years), family functioning (6–12 years), hostile‐reactive parenting (6–12 years), depressive symptoms (6–12 years), anxiety (10–12 years), oppositional‐defiant symptoms (6–12 years) and inattention/hyperactivity symptoms (6–12 years). Model 3 additionally adjusted for face‐to‐face victimization or cybervictimization at each given age. Model 4 additionally adjusted for suicidal ideation and attempt at baseline
Quintana‐Orts et al. (2022) Spain		Cybervictimisation The Spanish version of the European Cyberbullying Intervention Project Questionnaire (ECIPQ; Del Rey et al., 2015; Ortega‐Ruiz et al., 2016), self‐report questionnaire (in class), past 2 months	Suicidal ideation The Frequency of Suicidal Ideation Inventory (FSII; Chang & Chang, 2016), self‐report questionnaire (in class), past 12 months	β coefficients, *SE b, t* and *p* values with 95% CIs	Covariates: Gender, age and grade Moderator: Core self‐evaluation
Sigurdson et al. (2018) Youth and Mental Health Study (YMHS) Norway		Bullying victimization Measure used by Alsaker (2003), self‐report questionnaire (in class), past 6 months	1. Suicidal ideation 2. Self‐harm 3. Suicide attempts Suicidal ideation used 4 items from Mood and Feelings Questionnaire (Angold et al., 1987) and one item from Center for Epidemiologic Studies Depression Scale (Andrews et al., 1993), past 2 weeks. Own measure used for self‐harm and suicide attempts, self‐report questionnaire (by post/online), lifetime	Logistic GLMM regression, ORs with 95% CIs	Parents' SES, gender, time points and bullied status
Silberg et al. (2016) Virginia Twin Study of Adolescent Behavioral Development (VTSABD) and the Young Adult Follow‐Up Study (YAFU) USA		Bullying victimization Child and Adolescent Psychiatric Assessment (CAPA; Angold & Costello, 2000), interview (home, family member and child), past 3 months	Suicidal ideation DSM‐III‐R based Structured Clinical Interview (Spitzer et al., 1990), interview (telephone, participant), present	Logistic regression, ORs with 95% CIs	Not reported
Sourander et al. (2006) Finnish Family Competence Study (FCC) Finland		Bullying victimization Own measure, self‐report questionnaire (child and parents, postal), present	1. Self‐harm (ideation) 2. Self‐harm (acts) Own measure, self‐report questionnaire (child and parents, postal), past 6 months	Multinomial logistic regression analysis, ORs, Adjusted ORs with 95% CIs	Univariate: Gender; Multivariate, Model 1: Female sex, mother's health problems, self‐reports of deliberate self‐harm, nonintact family structure, CBCL total scores, learning difficulties, bullied Model 2: Female sex, mother's health problems, self‐reports of deliberate self‐harm, nonintact family structure, CBCL externalizing, learning difficulties, YSR internalizing, bullied Model 3: Female sex, mother's health problems, CBCL aggressivity, self‐reports of deliberate self‐harm, nonintact family structure, YSR somatic complaints, learning difficulties, bullied
Undheim and Sund (2013) Youth and Mental Health Study (YMHS) Norway		Bullying victimization Measure used by Alsaker (2003), self‐report questionnaire (in class), past 6 months	Suicidal ideation 4 items from Mood and Feelings Questionnaire (Angold et al., 1987) and one item from Center for Epidemiologic Studies Depression Scale (Andrews et al., 1993), self‐report questionnaire (in class), past 2 weeks	Multiple linear regression, Standardized and unstandardized beta coefficients	Model 1: Depression at age 15 (T2), gender, age, SES, and being bullied and being aggressive toward others at age 14 (T1) Model 2: as above, and additionally suicidal ideation at T1
Winsper et al. (2012) Avon Longitudinal Study of Parents and Children (ALSPAC) UK		Bullying victimization (including sub‐types) Bullying and Friendship Interview Schedule (Woods & Wolke, 2003), child interview at ages 8 and 10, past 6 months; own measure, mother interview at ages 4, 7, and 9, anytime; own measure, teacher interview at ages 7 and 10, anytime	1. Suicidal ideation 2. Suicidal/self‐injurious behavior Own measure, child interview, past 2 years	Logistic regression, ORs with 95% CIs	Model 1: Controlling for age and gender. Model 2: Controlling for age, gender, and additionally abuse, domestic violence, and maladaptive parenting. Model 3: Controlling for negative emotionality and conduct disorder in addition to age, gender, abuse, domestic violence, and maladaptive parenting.
Wu et al. (2021) China		Bullying victimization School Bullying/Victimization Scale (Chang et al., 2013), self‐reported questionnaire (in class), past year	NSSI Own measure, self‐reported questionnaire (in class), past 6 months	Logistic regression, ORs with 95% CIs	Age and gender
Zhu et al. (2021) China		Cybervictimisation 4‐item cybervictimization subscale in the Chinese version of the brief adaptation Electronic Bullying Questionnaire (EBQ) (Moore et al., 2012; Tian et al., 2018), self‐reported questionnaire (in class), past 6 months	NSSI Own measure, self‐reported questionnaire (in class), past 6 months	SEM, *β* and *B* coefficients and *p* values	Adolescent gender, age, childhood trauma. Other covariates were anxiety symptoms and NSSI at baseline

Abbreviations: aOR, adjusted odds ratio; BV, bullying victimization; CI, confidence interval; CV, cyberbullying victimization; GLMM, generalized linear mixed model; NSSI, nonsuicidal self‐injury; OR, odds ratio; PARP, population‐attributable risk proportion; RR, relative risk; SA, suicide attempt; SEM, structural equation model; SES, socioeconomic status; SH, self‐harm; SI, suicidal ideation.

### 3.3 Sample characteristics

Twenty‐two included studies used samples that were completely unique to their paper. Thirteen papers used samples that were similar to the samples of another paper due to being part of the same cohort study. However, they had slightly different sample sizes for the purpose of analysis based on the following reasons: inclusion of future waves (Lereya et al., 2013, 2015; Sigurdson et al., 2018; Undheim & Sund, 2013; Winsper et al., 2013), different age of participants and predictor of interest (Mars et al., 2020), different outcomes of interest (Copeland et al., 2013; Kiekens et al., 2019; Le et al., 2017, 2019; Mortier et al., 2017), or other unspecified reasons (Cho & Glassner, 2020; Cho, 2019).

Sample sizes ranged from 463 (Özdemir & Stattin, 2011) to 6,043 (Winsper et al., 2012). The male‐female ratio was fairly equal overall, with females overrepresented in two studies (Mars et al., 2020; Perret et al., 2020) and one study included males only (Klomek et al., 2008). Given the exclusion criteria, all studies collected data on the exposure (bullying) that took place before the age of 18; 24 studies measured the outcome between 11 and 18 (i.e., adolescence; Bannink et al., 2014; Benatov et al., 2021; Borschmann et al., 2020; Brunstein Klomek et al., 2019; Cho, 2019; Cho & Glassner, 2020; Fisher et al., 2012; Garisch & Wilson, 2015; Heikkilä et al., 2013; Hemphill et al., 2015; Kim et al., 2009; Le et al., 2017, 2019; Lereya et al., 2013; Lung et al., 2020; O'Connor et al., 2009; Özdemir & Stattin, 2011; Perret et al., 2020; Quintana‐Orts et al., 2022; Sourander et al., 2006; Undheim & Sund, 2013; Winsper et al., 2012; Wu et al., 2021; Zhu et al., 2021), 8 studies between the ages of 18 and 26 (i.e., young adulthood; Blasco et al., 2019; Copeland et al., 2013; Kiekens et al., 2019; Klomek et al., 2008; Lereya et al., 2015; Mars et al., 2020; Mortier et al., 2017; Silberg et al., 2016) and 3 looked at both (Geoffroy et al., 2021; Klomek et al., 2009; Sigurdson et al., 2018). One study (Sigurdson et al., 2018) included a time point with a mean age of 27.2, but was included as it was close to the threshold (and would have included some people aged 25 or younger) and met all other criteria. Data on sex/gender, ethnicity/nationality and social economic status was reported in 31 (89%), 11 (31%), and 11 (31%) of studies, respectively (Table 2).

**Table 2:** Summary of the exposures and outcomes across all studies

Characteristics	Total studies (*n*)^a^
Bullying type reported in study	
Traditional face‐to‐face	21
Cyberbullying victimisation	3
Both	11
Victim status	
Victims only	26
Victims and bully‐victims	9
Scale validation for exposure(s)^a^	
Yes—validated scale	21
No—unvalidated, used in other studies	5
No—unvalidated, new scale	11
Method of data collection for exposure	
Questionnaire	26
Peer nomination	1
Interview	8
SITB type(s) reported in study^a^	
NSSI	4
Self‐harm	10
Suicidal ideation	17
Suicide attempt	4
Other (multiple forms)	7
Scale validation for outcome(s)^a^	
Yes—validated scale	13
No—unvalidated, used in other studies	9
No—unvalidated, new scale	14
Medical record	1
Method of data collection for outcome	
Questionnaire	27
Interview	7
Medical record	1

Abbreviation: SITB, self‐injurious thoughts and behaviors.

^a^ Studies with multiple exposures/outcomes in one paper may be counted more than once (e.g., a study may have used one validated and one unvalidated scale to measure two different types of outcomes).

### 3.4 Exposure and outcome: Definition and types, assessment measurements, and methods

Across the 35 studies, 13 provided a definition of bullying to participants, 14 did not and in 6 studies it was unclear if a definition was provided. Of the 13 studies with a definition, eight contained all three components of bullying (i.e., power imbalance, intention to harm, repetition). Within the measure itself, 8 studies captured power imbalance^2^, 24 studies captured intention to harm^3^ and 32 studies captured repetition, although 15 studies classified bullying at a lower threshold than Solberg and Olweus (2003) frequently‐cited value (i.e., less than two or three times a month). An additional study used a lower threshold for cyberbullying but not traditional bullying (Perret et al., 2020). Eight studies included definitions *and* measures that captured all three elements of bullying (Blasco et al., 2019; Fisher et al., 2012; Garisch & Wilson, 2015; Heikkilä et al., 2013; Kiekens et al., 2019; Le et al., 2017, 2019; Mortier et al., 2017). These studies used the following measures: Bully Survey (Swearer & Cary, 2003), an own measure previously used with good test‐retest reliability (Fisher et al., 2012), two questions from the World Health Organization Youth Health Study (King et al., 1996), Revised Olweus Bully/Victim Questionnaire (Olweus, 1996), Peer Relations Questionnaire (Rigby & Slee, 1995). See Materials S5 for an overview of definitions and measures of each study.

Of the 11 studies that looked at both forms of bullying (i.e., traditional, face‐to‐face bullying *and* cyberbullying), 7 studies reported an aggregated measure (i.e., both types of bullying combined into one measure; Borschmann et al., 2020; Garisch & Wilson, 2015; Kiekens et al., 2019; Le et al., 2017, 2019; Lung et al., 2020; O'Connor et al., 2009) and 4 reported a disaggregated measure (i.e., conducting and reporting separate analyses for each type; Bannink et al., 2014; Benatov et al., 2021; Hemphill et al., 2015; Perret et al., 2020). The conceptualization of bullying in two studies (Lung et al., 2020; O'Connor et al., 2009) which used their own measure was vague and reported in this review as an aggregate measure of bullying due to limited information provided in the manuscript as to whether the questions asked were inclusive of cyberbullying or not. Two studies reported on the risk for different sub‐types of traditional bullying (e.g., relational, physical, verbal). Outcomes for bully perpetrators only is outside the remit of this systematic review. Nine studies used multi‐informant methods that included a combination of child and/or parent and/or teacher reports of bullying (Copeland et al., 2013; Fisher et al., 2012; Klomek et al., 2008, 2009; Lereya et al., 2013, 2015; Silberg et al., 2016; Sourander et al., 2006; Winsper et al., 2012). There was considerable heterogeneity in definitions and measurement of outcomes that ranged across the spectrum of self‐harm and suicidal thoughts and behaviors. Six studies reported more than one SITB outcome (e.g., the authors looked at self‐harm and suicidal thoughts and/or behaviors; Benatov et al., 2021; Brunstein Klomek et al., 2019; Kim et al., 2009; Mortier et al., 2017; Sigurdson et al., 2018; Winsper et al., 2012).

### 3.5 Quality assessment

The methodological quality of studies ranged from 3 to 8.5 (*M* = 6.3), out of a possible range of 0 to 9 (see Materials S6). One was categorized as low (0–3), 21 as medium (4–6) and 14 as high quality (7–9). Studies performed well in representativeness of their cohorts and the majority adjusted for confounders, aiming to minimize inaccurate conclusions from spurious associations. However, many studies failed to control for the outcome at the start of the study, limiting conclusions around causality as it could not be guaranteed that bullying preceded the outcome. There was considerable heterogeneity in choice of measurements to ascertain exposure and/or outcome, with many using unvalidated measures. Follow up times were good, with only two studies less than 6 months (Garisch & Wilson, 2015; Quintana‐Orts et al., 2022). High attrition rates increase the risk of bias, and this varied across studies, from 3% (Kim et al., 2009) to 62% (Bannink et al., 2014). However, drop out was often accounted for through attrition analysis and/or adjustment (e.g., using weights) where appropriate. Four studies did not report rates of attrition (Cho, 2019; Geoffroy et al., 2021; Lereya et al., 2013; Winsper et al., 2012).

### 3.6 Association between traditional bullying only and SITB

Twenty‐five studies collected data that measured traditional bullying (e.g., face‐to‐face and/or school‐based bullying; Bannink et al., 2014; Benatov et al., 2022; Blasco et al., 2019; Brunstein Klomek et al., 2019; Cho, 2019; Cho & Glassner, 2020; Copeland et al., 2013; Fisher et al., 2012; Geoffroy et al., 2021; Heikkilä et al., 2013; Hemphill et al., 2015; Kim et al., 2009; Klomek et al., 2008, 2009; Lereya et al., 2013, 2015; Mortier et al., 2017; Özdemir & Stattin, 2011; Perret et al., 2020; Sigurdson et al., 2018; Silberg et al., 2016; Sourander et al., 2006; Undheim & Sund, 2013; Winsper et al., 2012; Wu et al., 2021). Table 3 provides an overview of the main associations, the range of effect sizes and references to the included studies. The majority collected data on suicidal ideation (*n* = 14). Other outcomes were NSSI (*n* = 1), self‐harm (*n* = 6), suicidal attempt (*n* = 4) and other (aggregated) measures of SITBs (*n* = 7). Some papers measured multiple outcomes in separate analyses. Sixteen studies measured the outcomes in under 18s (Bannink et al., 2014; Benatov et al., 2022; Brunstein Klomek et al., 2019; Cho, 2019; Cho & Glassner, 2020; Fisher et al., 2012; Heikkilä et al., 2013; Hemphill et al., 2015; Kim et al., 2009; Lereya et al., 2013; Özdemir & Stattin, 2011; Perret et al., 2020; Sourander et al., 2006; Undheim & Sund, 2013; Winsper et al., 2012; Wu et al., 2021), six in young adulthood (Blasco et al., 2019; Copeland et al., 2013; Klomek et al., 2008; Lereya et al., 2015; Mortier et al., 2017; Silberg et al., 2016) and three studies looked at outcomes in both childhood and young adulthood (Geoffroy et al., 2021; Klomek et al., 2009; Sigurdson et al., 2018). Across included studies that reported ORs (*n* = 20), the effect of traditional bullying on the various measures of SITBs ranged from *aOR* 0.6, 95% confidence interval (*CI*) [0.2, 2.2] to *aOR* 18.5 [6.2, 55.1]. Two studies measured associations using RRs, ranging from *aRRs* 0.94, 95% *CI* [0.31, 2.49] to 2.44 [1.36, 4.40]. Three studies reported *ß* values, ranging from *ß* = .01 to .21. Associations were particularly large for youths who were both bullies and victims (i.e., bully‐victims; Copeland et al., 2013; Kim et al., 2009; Winsper et al., 2012) and for those frequently victimized (Fisher et al., 2012; Klomek et al., 2009; Lereya et al., 2013), although many studies (*n* = 18) did not differentiate between victims and bully‐victims, or used a dichotomous measure of bullying (i.e., yes/no, rather than run analyses based on severity/frequency). Only two studies (Brunstein Klomek et al., 2019; Winsper et al., 2012) looked at associations between bullying and SITB by subtype of traditional bullying (i.e., physical/verbal/relational and overt/relational). The effect of the different bullying sub‐types on the various measures of SITBs ranged from *aOR* 0.58 [95% *CI* not reported] to *aOR* 7.69 [95% *CI* not reported], with particularly large associations between physical bullying and suicidality. Materials S4 presents more details for each individual study, and Materials S7 presents a text‐based summary for each outcome under investigation.

**Table 3:** Table of associations between traditional bullying (i.e., face‐to‐face, including verbal, relational, physical bullying) and self‐harm/suicide, with effect sizes

Outcome	Author	Effect size^a^	
		Bivariable	Multivariable
NSSI	Wu et al. (2021)		*aOR*s: 1.26 [95% *CI*: not reported], *SE* = 0.30, *p* > .05–**2.76 [95%** ***CI***: **nr]**, ***SE*** = **0.26**, ***p*** = **.008**
Self‐harm	Fisher et al. (2012)	***RRs*** **: 2.553, 95%** ***CI***: **[1.23, 5.28]–4.92 [2.33, 10.40]**	***aRRs***: **1.92, 95%** ***CI***: **[1.18, 3.12]–2.44 [1.36, 4.40]**
	Lereya et al. (2013)	*ORs*: 1.18, 95% *CI*: [0.97, 1.43]–**2.68 [1.41, 5.11]**	*aOR*s: 1.13, 95% *CI*: [0.87, 1.47]–**4.75 [1.72, 13.07]**
	Sigurdson et al. (2018)		***aOR*** **s: 1.91, 95%** ***CI***: **[1.01, 3.63]–4.62 [2.47, 8.67]**
	Hemphill et al. (2015)		***aOR*** **s: 1.91, 95%** ***CI***: **[1.02, 3.58]–2.40 [1.34, 4.29]**
			NB. Nonsignificant *aOR* not reported
	Özdemir and Stattin (2011)		***ß*** **s** = **.13 [95%** ***CIs***: **not reported]**, ***p*** < **.05–0.21 [95%** ***CI***: **nr]**, ***p*** < **.05**
	Sourander et al. (2006)		*aORs*: 0.6, 95% *CI*: [0.2, 2.2]–**4.0 [1.4, 11.4]**
Suicidal ideation	Silberg et al. (2016)	***ORs***: **1.9, 95%** ***CI***: **[1.3, 3.0]–2.9 [1.2, 7.2]**	
	Mortier et al. (2017)	*OR*: 1.38, 95% *CI*: [0.80, 2.38]	
	Blasco et al. (2019)	***OR***: **2.4, 95%** ***CI***: **[1.55, 3.70]**	*aOR*s: 1.51, 95% *CI*: [0.80, 2.84]–**3.2 [1.08, 9.53]**
	K. Brunstein et al. (2008)	*ORs*: 1.2, 95% *CI*: [0.8, 2.0]–1.3 [0.96, 1.7]	*aOR*s: not reported*p*, > .05
	Bannink et al. (2014)		***aOR***: **1.57, 95%** ***CI***: **[1.21, 2.03]**
	Winsper et al. (2012)		***aOR*** **s: 1.57, 95%** ***CI***: **[1.15, 2.16]–3.20 [2.07, 4.95]**
	B. Klomek et al. (2019)		*aOR*s: 0.58 [95% *CI*: not reported], *p* > .05–**2.63** [95% *CI*: nr], ***p*** < **.05**
	Cho (2019)		*aOR*s: 1.32 [95% *CI*: not reported], *p* > .05–**1.70** [95% *CI* nr], ***p*** < **.05**
	Cho and Glassner (2019)		*ß*s = .014 [95% *CI*: not reported], *SE* = 0.106, *p* > .05–**0.089, 95%** ***CI*** **[0.023, 0.155]**
	Heikkila et al. (2013)		*aOR*s: 1.9, 95% *CI*: [0.4, 10.7]–**2.3 [1.0, 5.2]**
	Kim et al. (2009)		*aOR*s: 0.52, 95% *CI*: [0.11, 2.49]–**6.39 [1.58, 25.88]**
	Sigurdson et al. (2018)		*aOR*s: 1.76, 95% *CI*: [0.89, 3.49]–**3.63 [2.37, 5.57]**
	Undheim and Sund (2013)		*ß* = *.07 [95% CIs: not reported], t* = 4.1, ***p*** < .**001**
			NB. Nonsignificant *ß* not reported
	Benatov et al., (2021)		*aOR*s: 1.04, 95% *CI*: [0.67, 1.60]
Suicide attempt	B. Klomek et al. (2019)		*aOR*s: 0.51 [95% *CI*: not reported], *p* > .05–**7.69** [95% *CI*: nr], ***p*** < **.05**
	Sigurdson et al., 2018)		*aOR*s: 1.30, 95% *CI*: [0.49, 3.45]–**6.26 [2.94, 13.30]**
	Benatov et al. (2021)		*aOR*s: 1.15, 95% *CI*: [0.63, 2.09]
	Geoffroy et al. 2021)		*aRRs*: 0.94, 95% *CI*: [0.31, 2.49]–2.23 [0.53, 9.33.29]
Other	Mortier et al. (2017)	(Other: Suicidal plan) *OR*: 1.71, 95% *CI*: [0.98, 2.99]	
	Copeland et al (2013)	(Other: Suicidality) *ORs*: 1.6, 95% *CI*: [0.7, 4.0]–**5.5 [1.7, 17.4]**	*aOR*s: 0.6, 95% *CI*: [0.1, 3.9]–**18.5 [6.2, 55.1]**
	K. Brunstein et al., (2009)	(Other: Suicide attempt/death) *ORs*: 1.3, 95% *CI*: [0.5, 3.0]–**6.5 [2.1, 20.7]**	(Multivariable) *aOR*s: 1.5, 95% *CI*: [0.6, 3.7]–**6.3 [1.5, 25.9]**
	Lereya et al. (2015)	(Other: Self‐harm/suicidality) ***ORs*** **: 1.8, 95%** ***CI*** **: [1.4, 2.3]–3.0 [1.2, 8.0]**	(Multivariable) ***aOR*** **s: 1.7, 95%** ***CI***: **[1.4, 2.2]–3.0 [1.2, 7.7]**
	Perret et al. (2020)		(Other: Suicide ideation/attempt) ***aOR*** **s: 2.06, 95%** ***CI***: **[1.56, 2.72]–2.45 [1.82, 3.29]**
	Winsper et al. (2012)		(Other: Suicidal/self‐injurious behavior) ***aOR*** **s: 1.77 [1.31, 2.41]–3.34 [2.17, 5.15]**
	Kim et al. (2009)		(Other: Suicidal behaviors) *aOR*s: 0.53, 95% *CI*: [0.04, 6.75]–4.94 [0.86, 28.33]

*Note*: Effect sizes in **bold** indicate statistically significant *p* < .05.

Abbreviations: aOR, adjusted odds ratio; CI, confidence interval; nr, not reported; OR, (unadjusted) odds ratio; SE, standard error.

^a^ Indicates range of effect sizes given when there are multiple analyses with different outcomes/stratified results analyses within the same paper.

### 3.7 Association between cyberbullying only and SITB

Seven studies measured associations between cyberbullying and the following outcomes: NSSI (*n* = 1), self‐harm (*n* = 2), suicidal ideation (*n* = 3), suicide attempt (*n* = 1), aggregate of suicidal ideation/attempt (*n* = 1). Table 4 provides an overview of the main associations, the range of effect sizes and references to the included studies. Cyberbullying was measured at ages 12–13 in three studies (Bannink et al., 2014; Perret et al., 2020; Zhu et al., 2021), at age 15 in two studies (Benatov et al., 2022; Hemphill et al., 2015) and at 18 years in another (Mars et al., 2020). Outcomes were collected under 18 years of age except one (Mars et al., 2020) and one study reported results for both bully‐victims and victims only (Hemphill et al., 2015). Across included studies that reported ORs (*n* = 5), the effect of cyberbullying on the various measures of SITBs ranged from *aOR*s 0.87, 95% *CI* [0.36, 2.11] to 2.42 [1.41, 4.15]. Two studies reported *ß* values, ranging from *ß* = .04 to .38. Associations were largest for young women, and with self‐harm and suicidal ideation. Definitions of cyberbullying were provided in two studies (Benatov et al., 2022 and Perret et al., 2020) and none of the studies captured “power imbalance” in their measures. Materials S4 and S7 provide further study‐specific information.

**Table 4:** Table of associations between cyberbullying and self‐harm/suicide, with effect sizes

Outcome	Author		Effect size^a^	
			Bivariable	Multivariable
NSSI	Zhu et al. (2021)			***ß*** **s** = **.04, 95%** ***CI***: **[0.014, 0.083]–0.21 [95%** ***CI***: **not reported]**, ***p*** < **.05**
Self‐harm	Hemphill et al. (2015)		*OR*: 1.64, 95% *CI*: [0.76, 3.50]–**3.21, [1.51, 6.81]**	*aOR*: 0.87, 95% *CI*: [0.36, 2.11]–2.01 [0.82, 4.92]
				NB. Nonsignificant *aOR* not reported
	Mars et al. (2020)		*ORs*: 1.75, 95% *CI*: [0.39, 7.77]–**3.01 [1.82, 4.96]**	*aOR*s: 1.59, 95% *CI*: [0.35, 7.26]–**2.42 [1.41, 4.15]**
Suicidal ideation	Bannink et al. (2014)			*aOR*: 1.36, 95% *CI*: [0.81, 2.28]
	Benatov et al. (2022)			***aOR***: **1.88, 95%** ***CI***: **[1.08, 3.29]**
	Quintana‐Orts et al. (2022)			***ß*** **= .38 [95%** ***CI***: **not reported]**, ***p*** < **.001**
Suicide attempt	Benatov et al. (2022)			*aOR*s: 1.25, 95% *CI*: [0.58, 2.74]
Other	Perret et al. (2020)			Suicide ideation/attempt *aOR*s: 0.98, 95% *CI*: [0.73, 1.33]–1.37 [0.97, 1.93]

*Note*: Effect sizes in **bold** indicate statistically significant *p* < .05.

Abbreviations: aOR, adjusted odds ratio; CI, confidence interval; nr, not reported; OR, (unadjusted) odds ratio.

^a^ indicates range of effect sizes given when there are multiple analyses with different outcomes/stratified results analyses within the same paper.

### 3.8 Association between bullying (aggregate of traditional and cyberbullying) and SITB

Of the seven studies which aggregated all forms of bullying, five explicitly stated the use of a measure that aggregated items on traditional and cyberbullying (Borschmann et al., 2020; Garisch & Wilson, 2015; Kiekens et al., 2019; Le et al., 2017, 2019), and two were assumed to be an aggregate as they used an own measure with an unreported or broad definition (Lung et al., 2020; O'Connor et al., 2009). Table 5 provides an overview of the main associations, the range of effect sizes and references to the included studies. Outcomes were NSSI (*n* = 2), self‐harm (*n* = 3) and suicidal ideation (*n* = 2). Six studies measured outcomes in under 18s (Borschmann et al., 2020; Garisch & Wilson, 2015; Le et al., 2017; Le et al., 2019; Lung et al., 2020; O'Connor et al., 2009) and one in over 18s (Kiekens et al., 2019). Across the included studies that reported adjusted ORs (aOR) (*n* = 5), the effect of (aggregated) bullying on the various measures of SITBs ranged from *aOR*s 1.32, 95% *CI* [0.54, 3.23] to *aOR* 24.63 *CI* [3.83, 158.21]. One study looked at associations with self‐harm by reporting *ß* values (*ß* = .10) while another reported unadjusted *ORs* (1.06, 95% *CI* [0.32, 3.49] to 2.98 [1.15, 7.71]). Another study reported Pearson's *r* when looking at bullying and NSSI (*r* = .12). Associations were particularly large for children who were both bullies and victims (i.e., bully‐victims; Le et al., 2017; Le et al., 2019), and for those frequently victimized (Borschmann et al., 2020), although some samples had wide confidence intervals. This may be explained by failing to provide a definition of bullying to participants (e.g., Borschmann et al., 2020). Larger effect sizes within the same sample of participants may be explained by having a lower threshold for frequency of bullying. For example, Le et al. (2019) used a threshold of “once or twice a month” whereas Le et al. (2017) used the cutoff point “a few times a month.” Materials S4 and S7 provide further study‐specific information.

**Table 5:** Table of associations between aggregated bullying (traditional and cyber) and self‐harm/suicide, with effect sizes

Outcome	Author	Effect size^a^	
		Bivariable	Multivariable
NSSI	Garisch and Wilson (2016)		*r* = .12 (ns), *p* > .10
	Kiekens et al. (2019)	***ORs***: **1.9, 95%** ***CI*** **: [1.3, 2.6]–2.1 [1.5, 3.0]**	***aOR*** **s: 1.6, 95%** ***CI***: **[1.0, 2.5]–1.6 [1.0, 2.6]**
Self‐harm	Borschmann et al. (2020)	*ORs*: 6.00, 95% *CI*: [0.81, 44.36]–**23.05 [3.53, 150.55]**	*aOR*s: 6.78, 95% *CI*: [0.94, 49.07]–**24.63, [3.83, 158.21]**
	O'Connor et al. (2009)	*ORs*: 1.06, 95% *CI*: [0.32, 3.49]–**2.98 [1.15, 7.71]**	
	Lung et al. (2020)		***β*** = **.10 [95%** ***CI***: **not reported]**, ***p*** < **.001**
Suicidal ideation	Le et al. (2017)	***ORs***: 1.6**95%**, ***CI*** **: [0.5, 56.0]–8.9 [3.5, 22.5]**	*aOR*s: 1.7, 95% *CI*: [−0.3, 3.8]–6.5 **[2.2, 19.5]**
	Le et al. (2019)		*aOR*s: 1.32, 95% *CI*: [0.54, 3.23]–**2.30 [1.07, 4.92]**

*Note*: Effect sizes in **bold** indicate statistically significant *p* < .05.

Abbreviations: aOR, adjusted odds ratio; CI, confidence interval; nr, not reported; OR, (unadjusted) odds ratio

^a^ Indicates range of effect sizes given when there are multiple analyses with different outcomes/stratified results analyses within the same paper.

### 3.9 Influence of sex/gender on the association between bullying and SITB

Sex and/or gender was often included in multivariable models as a control variable and many studies provided prevalence rates of bullying and/or SITB by sex/gender (see Materials S7 and S8).

Four studies looked at whether sex/gender acted as a moderator in the association between bullying and SITB by adding an interaction term into their models (Bannink et al., 2014; Copeland et al., 2013; Perret et al., 2020; Sigurdson et al., 2018). One reported nonstatistically significant interactions without specifying the effect size (Perret et al., 2020), one did not report on the interaction terms (Sigurdson et al., 2018), and the other did not specify the interaction terms but stratified significant interactions by gender (Copeland et al., 2013). The final study, looking at suicidal ideation, reported small interactions for gender × traditional bullying (*aOR*: 1.41, 95% *CI*: [0.83, 2.33], *p* = .20) and gender × cyberbullying (*aOR*: 1.39 [0.56, 3.45], *p* = .48) but did not stratify the results due to a nonstatistically significant interaction (Bannink et al., 2014). Additionally, one study looked at the direct and indirect associations between sex and self‐harm via being bullied using path analysis (Lereya et al., 2013).

Four studies stratified all findings by sex/gender and did not present unstratified results (Kim et al., 2009; Klomek et al., 2009; Mars et al., 2020; Sigurdson et al., 2018), and three studies presented results that were stratified and unstratified by sex/gender (Fisher et al., 2012; Le et al., 2017, 2019). Five studies looked at associations with self‐harm, four with suicidal ideation, and four with other suicidal behaviors.

#### 3.9.1 Associations between bullying and self‐harm

The association with self‐harm by sex/gender was explored in one study of young adults (Mars et al., 2020), two studies in pre‐ to early adolescence (Fisher et al., 2012; Lereya et al., 2013), and a final study which looked at both mid‐adolescence and young adults (Sigurdson et al., 2018). In Mars et al. (2020), the association of cyberbullying with self‐harm was stronger for young women (*aOR*: 2.42, 95% *CI*: [1.41, 4.15]) than young men (*aOR*: 1.59 [0.35, 7.26]), while in Sigurdson et al. (2018), traditional bullying had a stronger association with self‐harm for young men (*aOR*: 3.86, 95% *CI*: [1.31, 11.41], *p* = .014) than women (*aOR*: 1.91, 95% *CI*: [1.01, 3.63], *p* = .047) although there was greater variability in scores for young men. The risk of self‐harm aged 12 after being bullied in preadolescence was high for both boys and girls (Fisher et al., 2012), with the associations strongest for boys when bullying was reported by the mother (*RR*: 4.92, 95% *CI*: [2.33, 10.40]) and strongest for girls when reported by the children themselves (*RR*: 4.16, [1.93, 8.95]). Finally, in a study using path analysis (Lereya et al., 2013), boys were significantly more likely to be bullied and girls more likely to self‐harm, and the association between the sex of the child and self‐harm via being bullied was stronger for boys (*β* = −.04, *SE* = 0.01, *p* = .001).

#### 3.9.2 Associations between bullying and suicidal ideation

In adjusted models, girls who were victims of bullying had 2.1–4.1 times greater odds of experiencing suicidal ideation compared to female nonvictims, with strong associations at both early/mid‐adolescence and young adulthood (Kim et al., 2009; Le et al., 2017; Le et al., 2019; Sigurdson et al., 2018). The pattern for boys was less clear, with odds of 0.94–3.63 as compared to male nonvictims. The association with suicidal ideation for victims only (in childhood) remained modest in young women (*aOR*: 2.68, 95% *CI*: [1.52, 4.73], *p* < .001) and smaller in young men (*aOR*: 1.76 [0.89, 3.49], *p* = .103; Sigurdson et al., 2018). For bully‐victims in Kim et al. (2009), the association with ideation was high for boys (*aOR*: 6.39, [1.58, 25.88] *p* < .01), and in Le et al. (2017) for girls (*aOR*: 6.50, 95% *CI*: [2.2, 19.5], *p* < .001).

#### 3.9.3 Associations between bullying and other suicidal behaviors

Finally, a strong effect for bullying on suicide attempts or death by suicide was found when bullying was frequent rather than occasional (Klomek et al., 2009), in both girls (*aOR*: 6.30, 95% *CI*: [1.50, 25.90]) and boys (*aOR*: 3.80, 95% *CI*: [0.99, 14.30]). Another Scandinavian study found high odds of suicide attempts in mid‐adolescence for boys (*aOR*: 6.26, 95% *CI*: [2.94, 13.30]) and girls (*aOR*: 3.90 [2.26, 6.73] bullied aged 13 (Sigurdson et al., 2018). These risks from childhood bullying continued into young adulthood for these same young men (: 6.06 [2.25, 16.36]) but not the young women in the sample (*aOR*: 1.30 [0.49, 3.45]; Sigurdson et al., 2018). For bully‐victims in a US‐based study (Copeland et al., 2013), the association with suicidality (i.e., self‐harm, suicidal ideation and attempts) was even higher for young adult men (*OR*: 18.5, 95% *CI*: [6.2, 55.1], *p* < .001) compared to young women (*OR*: 0.6 95% *CI*: [0.1, 3.9], *p* = .56), although these unadjusted ORs did not control for other variables.

## 4 DISCUSSION

### 4.1 Summary

The associations between bullying and/or peer victimization and the different components of SITBs have been explored in recent decades (Hong et al., 2015; John et al., 2018; Kim & Leventhal, 2008; Serafini et al., 2021). This review extends the literature by focusing on bullying victimization (i.e., characterized by repetition, power imbalance and intention to harm), separated by sub‐types (i.e., traditional, cyber or aggregated measures of both) across the broad spectrum of SITBs (from NSSI to attempted or completed suicide) in longitudinal studies that includes children and adolescents as well as young adults. Additionally, this review looks at whether these associations differ by sex/gender.

Bullying was frequently associated with SITBs, with small to large associations, most often in mid‐adolescence. This is generally unsurprising, due to profound developmental changes at this age and the wider influence of the social environment (Pfeifer & Allen, 2021). Traditional forms of bullying have also been reported as most present during early to mid‐adolescence (Kowalski et al., 2014). Most studies looked at the association between traditional bullying and suicidal ideation and/or self‐harm, or aggregated measures of self‐harm and suicidality. Fewer studies looked at the impact of bullying on NSSI, suicide attempts and completed suicide, outcomes among young adults, or the long‐term impact of cyberbullying. There were no noteworthy differences across countries, nor for studies with longer timespans, nor between smaller and larger studies. Findings were often mixed, with heterogeneity in study design and variables being explored. Some studies focused on traditional bullying *or* cyberbullying (or both combined), some explored the effects for bully‐victims and victims separately, some chose different confounders (or none at all) and few stratified their findings by sex or gender. For this reason, it is difficult to present consistent patterns of findings that can be generalized across groups.

With the few studies that stratified by sex/gender, this review has found strongest associations between bullying and suicide attempts in older adolescent boys and young men (particularly bully‐victims), and bullying and self‐harm and suicidal ideation in girls and young women. Despite the heterogeneity of findings in this review, this study highlights the importance of investigating the experience of different types of bullying (e.g., traditional bullying or cyberbullying; overt vs. relational bullying) and its frequency/chronicity, on different types of victims (i.e., those who are only victims or also perpetrators of bullying), at different ages, with results stratified by sex/gender. Future studies which provide this level of detail may help to better tailor any anti‐bullying prevention and intervention programs, rather than assuming victims are one homogenous group.

This review also extended previous reviews by looking at the spectrum of youth, from childhood into young adulthood. Although most studies looked at outcomes in childhood and adolescence (i.e., under 18), some studies explored and found negative outcomes for young adults who were victimized many years before. This included suicidal ideation in a twin study, and in young women, as well as self‐harm and suicide attempts among young men. Indeed, it is thought that life events that take place during periods of transition such as early to mid‐adolescence may have a longer‐lasting effect (de Moor et al., 2019; Graber et al., 2018). For example, research on self‐harm is often focused on teenagers, but research suggests older adults who self‐harm often have a history of this behavior (Troya et al., 2019), highlighting the importance of continued follow up to better understand the long‐term impact of victimization across the lifespan.

Overall, the studies were of good quality, and study quality was not related to outcomes. The highest rated studies considered multiple confounding factors and used well‐defined and validated measures to assess exposures and outcomes. Importantly, these studies also provided participants with a definition of bullying, and used measures that captured the three elements of bullying (i.e., power imbalance, intention to harm, repetition). Studies had smaller effect sizes when they scored lowest on the quality assessment and/or used measures that lacked a definition/examples of bullying or failed to capture several of the core element. For example, being a single‐item question in a large survey. Although less than half of studies controlled for baseline levels of the outcome (an issue for inferring causality), they regularly found small to large effect sizes, tentatively support directionality between bullying and SITBs. Future studies should account for this in the design or analysis stage.

Many studies failed to capture the component “power imbalance” within their measure of bullying, despite incorporating this within the definition of bullying at the start of their manuscript. This supports findings from a previous review on bullying measures (Vivolo‐Kantor et al., 2014) and is important because “power imbalance” is one of the two elements that differentiates bullying from peer victimization. Only two studies failed to capture any of the components of bullying in their measure (Lung et al., 2020; O'Connor et al., 2009), also scoring lower quality assessment scores, and were given less weight in the review's overall conclusions.

#### 4.1.1 Associations by type of bullying (traditional vs. cyber)

In this review, 21 studies measured only traditional, face‐to‐face bullying (2 of which looked at sub‐types of traditional bullying), 3 studies measured only cyberbullying, and 4 studies looked at both forms but presented separate analyses. Additionally, 7 studies looked at both forms in a combined measure. Due to the small numbers, it is difficult to draw confident conclusions about differences between the types although tentatively it may appear there were slightly weaker effects for cyberbullying, in studies that mostly measured outcomes in adolescence rather than young adulthood.

Although associations between cyberbullying and SITBs were found in mid‐adolescence after controlling for sociodemographic factors and baseline depression, many of these effects reduced after adjusting for baseline suicidality and/or traditional forms of bullying. The two forms of bullying are often associated (Kowalski et al., 2019; Zych & Farrington, 2021), with previous cross‐sectional studies finding some variance above and beyond traditional bullying, particularly suicidal ideation (Kowalski et al., 2014; van Geel et al., 2014). One explanation for our results is the younger age range of our studies due to the requirement of cyberbullying occurring before 18 years old; negative outcomes from cyberbullying may appear later than traditional bullying (Bannink et al., 2014), with prevalence of cyberbullying thought to peak in mid‐adolescence but may reappear in young adulthood. For this reason, future studies may wish to explore cyberbullying that starts in young adulthood, supporting previous recommendations (Kowalski et al., 2019).

Additionally, the two studies that looked at sub‐types of traditional bullying had some striking findings. Victims of chronic physical bullying (i.e., persists over time) may have over seven times more risk of suicide attempts in mid‐adolescence compared to non‐victims (Brunstein Klomek et al., 2019). Worryingly, at the age of 11, victims of overt bullying (e.g., physical and verbal) may be 2.5 times more likely to engage in suicidal or self‐injurious behaviors (Winsper et al., 2012). There is clearly a space for future longitudinal research studies to consider looking at bullying sub‐types, to better understand patterns of behavior among different groups of young people.

#### 4.1.2 Associations by sex/gender

In the present review, less than 20% of studies looked at the moderating effect of sex/gender in the association between bullying and SITBs. Similar to previous reviews (Heerde & Hemphill, 2019; Holt et al., 2015; John et al., 2018), the role of sex/gender in these associations was not fully clear. However, closer inspection revealed some interesting patterns worth further exploration. First, although rates of bullying were not massively different between boys and girls, boys were more likely to be bully‐victims, a group at higher risk of negative mental health outcomes compared to pure victims or bullies (Hunter et al., 2007; Menin et al., 2021). The small number of papers that stratified by sex/gender and victim status results found some alarmingly high rates of suicidal behaviors in boys and young men, particularly bully‐victims. This group may be at most risk due to experiencing internalizing and externalizing behaviors, warranting further study (Kelly et al., 2015). For example, by incorporating measures to assess co‐occurrence of bullying victim/perpetration status (Jadambaa et al., 2019), and looking at the influence of sex/gender.

Second, only two studies looked at outcomes from specific sub‐types of traditional forms of face‐to‐face bullying, which helps to better understand any nuances between bullying and SITBs, across genders. Research suggests that girls are more likely to be victims of relational bullying (e.g., gossiping or exclusion), and boys of physical bullying (Crick & Bigbee, 1998). Relational bullying, including gossiping and exclusion, has shown a stronger link with suicidal ideation, while physical bullying is more associated with suicidal acts (Van der Wal et al., 2003; Zhao & Yao, 2022). Repeated exposure to physical bullying may increase tolerance to pain; in turn, this may provide the acquired capability to transition from suicidal ideation to acts, according to the interpersonal theory of suicide (Brunstein Klomek et al., 2019; Joiner, 2007). This may explain our findings that traditional bullying in boys may have a stronger association with suicidal behaviors in late adolescence and early adulthood, whereas it is more strongly associated with ideation in girls and young women. However, these conclusions cannot be confirmed in the present review, as few studies looked at the association between bullying sub‐types and SITBs. It is clear that future studies would benefit from stratifying by sex/gender and looking at sub‐types of bullying to better understand the trajectories over time, enhancing bullying prevention strategies and more tailored support.

### 4.2 Methodological issues

Although studies were all longitudinal, community‐based studies, there was great heterogeneity in measurements used, scope and definitions of the key concepts, whether the outcome was controlled at baseline, and statistical methods used to interpret the results (specifically, reliance on *p* values).

A range of measurements were used to ascertain bullying and the SITBs, with many being unvalidated measures, particularly for the outcome(s). Indeed, several studies aggregated several outcomes (e.g., self‐harm and suicidal ideation, or simply said “suicidality”), resulting in 10 unique types of outcome overall. Studies rated higher in quality regularly provided a clear definition, with examples, of bullying and/or the outcome to study participants, with authors signposting the reader to an example text. This is important for ensuring the correct concept is being measured.

Moreover, with cyberbullying being another potentially more subtle form of bullying, well‐defined, validated self‐report measures are clearly necessary to gauge an accurate picture of the extent of the problem (Olweus & Limber, 2018). Providing a clear working definition tailored to the target audience and/or presenting participants with a list of experiences is one such step, alongside focus groups with young people themselves to prevent any disconnect with researchers' definitions (Furlong et al., 2010; Menin et al., 2021). First, this will help better understand who is most at risk. Second, traditional bullying prevention programs can be adapted to better address the nuances of cyberbullying (Olweus et al., 2019).

This review found that *who* reports on the bullying is an important consideration. In the two studies (ALSPAC and E‐Risk) that presented findings according to whether bullying aged 7–10 years was reported by the child, mother and/or teacher, associations were strongest for boys when the mother and teacher reports were included. It has been suggested that indirect forms of bullying may be more subtle and missed by adults, possibly underestimating bullying in these children, who are more likely to be girls (Husky et al., 2022). Despite the methodological limitations of self‐report data, these measures may therefore better capture power imbalance and intention to harm that other informants may miss (Furlong et al., 2010; Jadambaa et al., 2019).

Longitudinal research aims to enhance the ability to draw conclusions about causality, for example by ensuring the exposure precedes the outcome. Observational study designs are most appropriate for harmful exposures, due to the unethical implications of manipulating exposure to bullying. Unfortunately, only 11 out of 35 studies controlled for baseline levels of the outcome under investigation, providing less certainty that bullying preceded self‐harm or suicidal behavior and there is potential for reverse causality (i.e., a person displaying self‐harming or suicidal behaviors may become a target of being bullied).

Finally, many studies made conclusions based on *p* values rather than interpreting effect sizes, and confidence intervals were often missing. This inhibited the opportunity, at times, to draw meaningful inferences to the wider population with any degree of certainty.

### 4.3 Future research

This review found several gaps in the literature that future studies should address.

First, there are very few prospective, longitudinal studies that look at cyberbullying and SITBs. Prevalence rates of cyberbullying often appear consistently lower than traditional bullying (Modecki et al., 2014), and the power to detect statistically significant differences are reduced when looking at an uncommon exposure such as cyberbullying and an uncommon outcome such as suicidality (Bannink et al., 2014). For this reason, future studies should not draw conclusions based solely on statistical significance testing. Rather, strength of effects should be explored, and qualitative studies should be conducted that have potential to generate greater depth of understanding about the experience of being a victim of cyberbullying. Moreover, previous studies suggest suicides linked to cyberbullying are often associated with other proximal risk factors (Hinduja & Patchin, 2010). Research should look at better understanding the different environmental factors which may work together and exacerbate feelings of perceived burdensomeness and thwarted belonginess—elements of suicidal ideation—and ways to reduce this risk (Joiner, 2007).

Second, future studies should collect data on socioeconomic status and ethnicity, as this was captured in less than a quarter of studies. It is, therefore, unclear if the association between bullying and SITBs could be generalized across sociodemographic groups. There may be specific nuances faced within or between people of different ethnicities (Kuldas et al., 2021), with victimization greater among poorer students (Hosozawa et al., 2021). Indeed, current in‐school bullying prevention programs may be less effective in minority ethnic groups, therefore highlighting the importance of understanding the nature of bullying across diverse populations (Limber et al., 2018). Moreover, studies would benefit from recruiting samples in diverse, urban areas, in preparation for the future direction of global development (i.e., greater urbanization). This is particularly important as 55% of the world's population live in urban areas, a proportion expected to increase to 68% by 2050 (Valencia‐Agudo et al., 2018).

Finally, given the potential nuances in experiences of bullying by boys and girls, as highlighted above, future studies would benefit from stratifying results by sex/gender. Sample sizes, if small, may result in false negative findings, once again highlighting the limitations of relying on *p* values when interpreting gender × bullying interaction terms, for example. The few studies which stratified results in this review had some interesting findings which may have been missed if adolescents are treated as a homogonous group with respect to sex/gender.

### 4.4 Strengths and limitations

A key strength of this review is the focus on prospective studies, which can better explore the direction of effect between bullying and SITBs.

There is ongoing discussion about whether cyberbullying and traditional face‐to‐face bullying are distinct or overlapping constructs (Olweus, 2010; Walker et al., 2013), and this review adds to the literature by presenting results grouped based on the original conceptualizations of authors and the respective measures, as recommended in a previous review (Camerini et al., 2020). Few prospective studies have looked at cyberbullying and SITBs, something which is greatly warranted given the rise of smartphone ownership among young people and the need to disentangle causal relationships from findings in cross‐sectional studies (John et al., 2018).

As with any review, there are limitations. There is debate about whether cyberbullying should be better conceptualized as cyberaggression, and the current review took a restrictive approach that drew on the traditional definition of bullying (Olweus & Limber, 2018). Moreover, the age limit was restricted to under 18s for the exposure, despite cyberbullying potentially continuing into young adulthood. Although few studies were excluded on this basis, some relevant studies may have been missed. The quality assessment scores should be interpreted with some caution; across similar reviews, many scales have been heavily adapted (Epstein et al., 2020; Latham et al., 2021; Moore et al., 2017), suggesting they may not be fit‐for‐purpose in studies looking at bullying and suicidality.

Finally, the decision to focus on bullying victimization was based on arguments in the literature (Furlong et al., 2010), and studies looking at the broader construct of peer victimization were excluded. The decision to restrict exposure types in this way may have excluded some studies looking at peer victimization with findings of some relevance. Moreover, there remains great heterogeneity of measurement in the bullying literature (Vivolo‐Kantor et al., 2014), with many studies failing to capture the three core components that have general consensus with researchers: repetition, intention to harm, power imbalance (Farrington, 1993; Olweus, 1994, 2010; Smith & Brain, 2000; Younan, 2019). Although the present review only included papers with measures explicitly stated as “bullying,” there is the possibility that some studies may in fact be capturing peer victimization. Greater precision of terms being measured is required, building on existing good practice and drawing on arguments in the literature (Furlong et al., 2010; Quinlan et al., 2020).

## 5 CONCLUSION

The present review has found prospective associations of varying effect sizes between bullying and self‐harm and suicidal thoughts and behaviors. The field is marked with great heterogeneity in terms of methodologies, making it difficult to draw concrete conclusions. Future research should aim to capture the nuances of bullying (e.g., by sub‐type and frequency) and its impact across the spectrum of SITBs, at different ages, among bullies, victims and bully‐victims. Importantly, results should be stratified by sex/gender, to better understand the complex dynamics that could be targeted in anti‐bullying interventions, and tailor support for victims of bullying.

## AUTHOR CONTRIBUTIONS

**Emma Wilson**: conceptualization, methodology, formal analysis, investigation, writing ‐ original draft, writing ‐ review & editing, visualization, project administration. **Holly Crudgington**: conceptualization, validation, formal analysis, investigation. Craig Morgan: conceptualization, methodology, supervision, writing ‐ review & editing, funding acquisition. **Colette Hirsch**: conceptualization, methodology, supervision, writing ‐ review & editing. **Matthew Prina**: methodology, writing ‐ review & editing. **Charlotte Gayer‐Anderson**: conceptualization, validation, methodology, supervision, writing ‐ review & editing.

## CONFLICTS OF INTEREST

The authors declare no conflicts of interest.

## ETHICS STATEMENT

Ethics approval was not needed for this review paper.

## Supporting information

Supporting information.

Supporting information.

Supporting information.

Supporting information.

Supporting information.

Supporting information.

Supporting information.

Supporting information.

## Data Availability

All additional materials are available as Supporting Information Materials.

## Footnotes

1 A note on terminology: In line with the United Nation's (1989) Convention on the Rights of the Child, “child” refers to anyone under 18 years old; in line with the World Health Organization's definitions, “adolescence” refers to 10–19 years, “youth” refers to 15–24 years, and “young people” refers to anyone aged 10–24 years old (although for the purpose of our review it also includes anyone aged 25 years of age); ‘young adults' in this review refer to people aged 18–25.

2 In four studies, it was not clear whether power imbalance was captured in the measure.

3 In four studies, it was not clear whether intention to harm was captured in the measure.
