# Supplementary material for: The longitudinal course of childhood bullying victimization and associations with self‐injurious thoughts and behaviors in children and young people: A systematic review of the literature
Source: J Adolesc. 2022 Oct 9;95(1):5–33. doi: 10.1002/jad.12097 (PMC10092090; doi:10.1002/jad.12097)
Supplement: Supplementary file 2 — Supporting information. [file JAD-95-5-s002.docx]

OVID

**Medline**

1. (self harm* or self injur* or self cut* or self destruct* or self mutil* or self poison* or self inflict* or automutilat* or auto mutilat* or suicid* or para-suicid* or parasuicid* or life-threatening behavio*).mp. [mp=title, abstract, original title, name of substance word, subject heading word, floating sub-heading word, keyword heading word, organism supplementary concept word, protocol supplementary concept word, rare disease supplementary concept word, unique identifier, synonyms]

2. ((NSSI or (nonsuicid* or non suicid*)) adj2 (self* or injury*)).mp. [mp=title, abstract, original title, name of substance word, subject heading word, floating sub-heading word, keyword heading word, organism supplementary concept word, protocol supplementary concept word, rare disease supplementary concept word, unique identifier, synonyms]

3. ((nonfatal or non fatal) adj2 (overdose* or over dose*)).mp. [mp=title, abstract, original title, name of substance word, subject heading word, floating sub-heading word, keyword heading word, organism supplementary concept word, protocol supplementary concept word, rare disease supplementary concept word, unique identifier, synonyms]

4. (self adj2 (cut* or burn* or bit* or hit*)).mp. [mp=title, abstract, original title, name of substance word, subject heading word, floating sub-heading word, keyword heading word, organism supplementary concept word, protocol supplementary concept word, rare disease supplementary concept word, unique identifier, synonyms]

5. (bully* or bullie* or harassment* or victimi?ation or peer aggression or peer victim* or peer abuse or school violence or cyberbull* or cybervictimi?ation).mp. [mp=title, abstract, original title, name of substance word, subject heading word, floating sub-heading word, keyword heading word, organism supplementary concept word, protocol supplementary concept word, rare disease supplementary concept word, unique identifier, synonyms]

6. (adoles* or young people or youth* or child* or teen* or juv* or young adult or young person or pupils).mp. [mp=title, abstract, original title, name of substance word, subject heading word, floating sub-heading word, keyword heading word, organism supplementary concept word, protocol supplementary concept word, rare disease supplementary concept word, unique identifier, synonyms]

7. (longitudinal or trajector* or prospective or course or time point* or follow-up or wave*).mp. [mp=title, abstract, original title, name of substance word, subject heading word, floating sub-heading word, keyword heading word, organism supplementary concept word, protocol supplementary concept word, rare disease supplementary concept word, unique identifier, synonyms]

8. exp Self-Injurious Behavior/

9. exp Bullying/

10. exp Adolescent/ or exp Adolescent Behavior/ or exp Adolescent Health/ or exp Adolescent Psychiatry/ or exp Psychology, Adolescent/

11. exp Young Adult/

12. cohort studies/ or follow-up studies/ or longitudinal studies/ or prospective studies/

13. 1 or 2 or 3 or 4 or 8

14. 5 or 9

15. 6 or 10 or 11

16. 7 or 12

17. 13 and 14 and 15 and 16

18. Limit 18 to English Language

**PsychINFO**

1. (self harm* or self injur* or self cut* or self destruct* or self mutil* or self poison* or self inflict* or automutilat* or auto mutilat* or suicid* or para-suicid* or parasuicid* or life-threatening behavio*).mp. [mp=title, abstract, heading word, table of contents, key concepts, original title, tests & measures, mesh]

2. ((NSSI or (nonsuicid* or non suicid*)) adj2 (self* or injury*)).mp. [mp=title, abstract, heading word, table of contents, key concepts, original title, tests & measures, mesh]

3. ((nonfatal or non fatal) adj2 (overdose* or over dose*)).mp. [mp=title, abstract, heading word, table of contents, key concepts, original title, tests & measures, mesh]

4. (self adj2 (cut* or burn* or bit* or hit*)).mp. [mp=title, abstract, heading word, table of contents, key concepts, original title, tests & measures, mesh]

5. (bully* or bullie* or harassment* or victimi?ation or peer aggression or peer victim* or peer abuse or school violence or cyberbull* or cybervictimi?ation).mp. [mp=title, abstract, heading word, table of contents, key concepts, original title, tests & measures, mesh]

6. (adoles* or young people or youth* or child* or teen* or juv* or young adult or young person or pupils).mp. [mp=title, abstract, heading word, table of contents, key concepts, original title, tests & measures, mesh]

7. (longitudinal or trajector* or prospective or course or time point* or follow-up or wave*).mp. [mp=title, abstract, heading word, table of contents, key concepts, original title, tests & measures, mesh]

8. exp Self-Injurious Behavior/

9. exp self-destructive behavior/

10. exp Attempted Suicide/ or exp Suicidal Ideation/

11. exp bullying/

12. exp Adolescent Psychiatry/ or exp Adolescent Behavior/ or exp Adolescent Psychology/ or exp Adolescent Health/

13. exp Child Psychiatry/ or exp Child Behavior/ or exp Child Psychology/ or exp Child Health/

14. exp emerging adulthood/

15. exp longitudinal studies/ or exp prospective studies/ or exp followup studies/

16. 1 or 2 or 3 or 4 or 8 or 9 or 10

17. 5 or 11

18. 6 or 12 or 13 or 14

19. 7 or 15

20. 16 and 17 and 18 and 19

21. Limit 20 to English Language

**EMBASE**

1. (self harm* or self injur* or self cut* or self destruct* or self mutil* or self poison* or self inflict* or automutilat* or auto mutilat* or suicid* or para-suicid* or parasuicid* or life-threatening behavio*).mp. [mp=title, abstract, heading word, drug trade name, original title, device manufacturer, drug manufacturer, device trade name, keyword, floating subheading word, candidate term word]

2. ((NSSI or (nonsuicid* or non suicid*)) adj2 (self* or injury*)).mp. [mp=title, abstract, heading word, drug trade name, original title, device manufacturer, drug manufacturer, device trade name, keyword, floating subheading word, candidate term word]

3. ((nonfatal or non fatal) adj2 (overdose* or over dose*)).mp. [mp=title, abstract, heading word, drug trade name, original title, device manufacturer, drug manufacturer, device trade name, keyword, floating subheading word, candidate term word]

4. (self adj2 (cut* or burn* or bit* or hit*)).mp. [mp=title, abstract, heading word, drug trade name, original title, device manufacturer, drug manufacturer, device trade name, keyword, floating subheading word, candidate term word]

5. (bully* or bullie* or harassment* or victimi?ation or peer aggression or peer victim* or peer abuse or school violence or cyberbull* or cybervictimi?ation).mp. [mp=title, abstract, heading word, drug trade name, original title, device manufacturer, drug manufacturer, device trade name, keyword, floating subheading word, candidate term word]

6. (adoles* or young people or youth* or child* or teen* or juv* or young adult or young person or pupils).mp. [mp=title, abstract, heading word, drug trade name, original title, device manufacturer, drug manufacturer, device trade name, keyword, floating subheading word, candidate term word]

7. (longitudinal or trajector* or prospective or course or time point* or follow-up or wave*).mp. [mp=title, abstract, heading word, drug trade name, original title, device manufacturer, drug manufacturer, device trade name, keyword, floating subheading word, candidate term word]

8. exp automutilation/

9. exp suicidal behavior/

10. exp bullying/

11. exp adolescent/ or exp child psychiatry/ or exp adolescent behavior/ or exp child psychology/ or exp adolescent health/

12. exp young adult/

13. exp longitudinal study/ or exp prospective study/ or exp follow up/ or exp cohort analysis/

14. 1 or 2 or 3 or 4 or 8 or 9

15. 5 or 10

16. 6 or 11 or 12

17. 7 or 13

18. 14 and 15 and 16 and 17

19. limit 18 to english language

Scopus

( TITLE-ABS-KEY ( bully* OR bullie* OR harassment* OR victimi?ation OR "peer aggression" OR "peer victim*" OR "peer abuse" OR "school violence" OR cyberbull* OR cybervictimi?ation ) ) AND ( TITLE-ABS-KEY ( adoles* OR "young people" OR youth* OR child* OR teen* OR juv* OR "young adult" OR "young person" OR pupils ) ) AND ( TITLE-ABS-KEY ( longitudinal OR trajector* OR prospective OR course OR "time point*" OR "follow-up" OR "follow up" OR wave* ) ) AND ( ( TITLE-ABS-KEY ( "self harm*" OR "self injur*" OR "self cut*" OR "self destruct*" OR "self mutil*" OR "self poison*" OR "self inflict*" OR "automutilat*" OR "auto mutilat*" OR "suicid*" OR "para-suicid*" OR "parasuicid*" OR "life-threatening behavio*" ) OR TITLE-ABS-KEY ( ( nssi OR ( "nonsuicid*" OR "non suicid*" ) ) W/2 ( self* OR injury* ) ) OR TITLE-ABS-KEY ( ( ( nonfatal OR "non fatal" ) n/2 ( overdose* OR "over dose*" ) ) ) OR TITLE-ABS-KEY ( ( self AND n/2 ( cut* OR burn* OR bit* OR hit* ) ) ) ) ) AND LANGUAGE ( english )

CINAHL

S14 AND S15 AND S16 AND S17 Expanders - Apply equivalent subjects

Search modes - Boolean/Phrase Interface - EBSCOhost Research Databases

Search Screen - Advanced Search

Database - CINAHL 194 EditS18

S17 S7 OR S13 Expanders - Apply equivalent subjects

Search modes - Boolean/Phrase Interface - EBSCOhost Research Databases

Search Screen - Advanced Search

Database - CINAHL 924,990 EditS17

S16 S6 OR S11 OR S12 Expanders - Apply equivalent subjects

Search modes - Boolean/Phrase Interface - EBSCOhost Research Databases

Search Screen - Advanced Search

Database - CINAHL 1,448,670 EditS16

S15 S5 OR S8 Expanders - Apply equivalent subjects

Search modes - Boolean/Phrase Interface - EBSCOhost Research Databases

Search Screen - Advanced Search

Database - CINAHL 22,394 EditS15

S14 S1 OR S2 OR S3 OR S4 OR S9 OR S10 Expanders - Apply equivalent subjects

Search modes - Boolean/Phrase Interface - EBSCOhost Research Databases

Search Screen - Advanced Search

Database - CINAHL 52,339 EditS14

S13 (MH "Prospective Studies+") Limiters - English Language

Search modes - Boolean/Phrase Interface - EBSCOhost Research Databases

Search Screen - Advanced Search

Database - CINAHL 463,066 EditS13

S12 (MH "Young Adult") Limiters - English Language

Search modes - Boolean/Phrase Interface - EBSCOhost Research Databases

Search Screen - Advanced Search

Database - CINAHL 257,642 EditS12

S11 (MH "Adolescence+") OR (MH "Child Psychiatry") OR (MH "Child Psychology") OR (MH "Adolescent Health") OR (MH "Adolescent Behaviour") Limiters - English Language

Search modes - Boolean/Phrase Interface - EBSCOhost Research Databases

Search Screen - Advanced Search

Database - CINAHL 541,923 EditS11

S10 (MH "Injuries, Self-Inflicted+") OR (MH "Self-Injurious Behavior+") Limiters - English Language

Search modes - Boolean/Phrase Interface - EBSCOhost Research Databases

Search Screen - Advanced Search

Database - CINAHL 6,780 EditS10

S9 (MH "Suicide, Attempted+") OR (MH "Suicidal Ideation+") Limiters - English Language

Search modes - Boolean/Phrase Interface - EBSCOhost Research Databases

Search Screen - Advanced Search

Database - CINAHL 13,115 EditS9

S8 (MH "Bullying+") Limiters - English Language

Search modes - Boolean/Phrase Interface - EBSCOhost Research Databases

Search Screen - Advanced Search

Database - CINAHL 9,075 EditS8

S7 TX longitudinal or trajector* or prospective or course or time point* or follow-up or wave* Limiters - English Language

Search modes - Boolean/Phrase Interface - EBSCOhost Research Databases

Search Screen - Advanced Search

Database - CINAHL 924,352 EditS7

S6 TX adoles* or young people or youth* or child* or teen* or juv* or young adult or young person or pupils Limiters - English Language

Search modes - Boolean/Phrase Interface - EBSCOhost Research Databases

Search Screen - Advanced Search

Database - CINAHL 1,448,670 EditS6

S5 TX bully* or bullie* or harassment* or victimi?ation or peer aggression or peer victim* or peer abuse or school violence or cyberbull* or cybervictimi?ation Limiters - English Language

Search modes - Boolean/Phrase Interface - EBSCOhost Research Databases

Search Screen - Advanced Search

Database - CINAHL 22,394 EditS5

S4 TX (self N2 (cut* or burn* or bit* or hit*)) Limiters - English Language

Search modes - Boolean/Phrase Interface - EBSCOhost Research Databases

Search Screen - Advanced Search

Database - CINAHL 778 EditS4

S3 TX (nonfatal or non fatal) N2 (overdose* or over dose*) Limiters - English Language

Search modes - Boolean/Phrase Interface - EBSCOhost Research Databases

Search Screen - Advanced Search

Database - CINAHL 343 EditS3

S2 TX (NSSI or (nonsuicid* or non suicid*)) N2 (self* or injury*) Limiters - English Language

Search modes - Boolean/Phrase Interface - EBSCOhost Research Databases

Search Screen - Advanced Search

Database - CINAHL 1,018 EditS2

S1 TX self harm* or self injur* or self cut* or self destruct* or self mutil* or self poison* or self inflict* or automutilat* or auto mutilat* or suicid* or para-suicid* or parasuicid* or life-threatening behavio*

Web of science

1. ALL = (self harm$ or self injur$ or self cut$ or self destruct$ or self mutil$ or self poison$ or self inflict$ or automutilat$ or auto mutilat$ or suicid$ or para-suicid$ or parasuicid$ or life-threatening behavio$) *AND***LANGUAGE:** (English) *AND* **DOCUMENT TYPES:** (Article)

2. AB=(NSSI NEAR/2 self$ or injury$)
