# Supplementary material for: The longitudinal course of childhood bullying victimization and associations with self‐injurious thoughts and behaviors in children and young people: A systematic review of the literature
Source: J Adolesc. 2022 Oct 9;95(1):5–33. doi: 10.1002/jad.12097 (PMC10092090; doi:10.1002/jad.12097)
Supplement: Supplementary file 3 — Supporting information. [file JAD-95-5-s006.docx]

# Newcastle Ottawa Scale scoring sheet

| **Area** | **Quality Criteria** | **Quality Score** |
| --- | --- | --- |
| Selection (of cohorts) | 1. Representativeness of exposed cohort  (max one star) | Truly representative of young people exposed to bullying in the target population (random sampling or whole population) = 1 star  Somewhat representative of young people exposed to bullying in the target population (non-random sampling including purposive sampling of representative schools or evidence the sample represents the source population) = 1 star  Selected group of users = 0  No description of how cohort is selected = 0 |
|  | 2. Selection of non-exposed cohort  (max one star) | Drawn from same community as exposed cohort = 1 star  Drawn from a different source = 0  No description of how non-exposed cohort is selected = 0 |
|  | 3. Ascertainment of exposure to bullying  (max one star) | Secure record (e.g., clinician reported or medical record) = 1 star  Structured interview or self-report questionnaire using validated measure = 1 star  Unstructured interview or self-report questionnaire using unvalidated measure = 0  No description = 0 |
|  | 4. Demonstration that outcome of interest was not present or accounted for at start of study (i.e., only includes participants without the outcome at baseline or outcome was collected and controlled for at baseline)  (max one star) | Yes = 1 star  No = 0 |
| Comparability (of cohorts) | 1. Comparability of victims of bullying vs. non-victims was increased on basis of design or analysis (i.e., appropriate methods to control confounding)  (max two stars) | Study controls for age and sex/gender (or analyses presented separately by gender) = 1 star  Study controls for any other additional factor = 1 star |
| (Assessment of) Outcome | 1. Assessment of outcome (self-harm, suicidality)  (max one star) | Independent, blind assessment stated or confirmation of the outcome by reference to secure records (e.g., medical records) or record linkage = 1 star  Structured interview or self-report questionnaire using validated measure = 1 star  Unstructured interview or self-report questionnaire using unvalidated measure = 0  No description = 0 |
|  | 2. Was follow-up long enough for outcome(s) to occur?  (max one star) | Yes, 6 months = 1 star  No = 0 |
|  | 3. Adequacy of follow-up of cohorts (where relevant) or response rate  (max one star) | Complete follow up (all subjects accounted for) = 1 star  Subjects lost to follow up unlikely to introduce bias - small number lost (≥20%) or description of those lost to follow-up (attrition is described and accounted for in the analyses) = 1  Follow up rate poor (<80%) and no statement about attrition = 0 |

Note. Some adaptations to the original NOS for nonrandomised studies were made based on the NOS scoring sheets from the following studies:

Epstein, S., Roberts, E., Sedgwick, R., Polling, C., Finning, K., Ford, T., Dutta, R., & Downs, J. (2020). School absenteeism as a risk factor for self-harm and suicidal ideation in children and adolescents: a systematic review and meta-analysis. *European Child & Adolescent Psychiatry*, *29*(9), 1175-1194. <https://doi.org/10.1007/s00787-019-01327-3>

Latham, R. M., Newbury, J. B., & Fisher, H. L. (2021). A systematic review of resilience factors for psychosocial outcomes during the transition to adulthood following childhood victimisation. *Trauma, Violence, & Abuse*, 15248380211048452. <https://doi.org/10.1177/15248380211048452>

Moore, S. E., Norman, R. E., Suetani, S., Thomas, H. J., Sly, P. D., & Scott, J. G. (2017). Consequences of bullying victimization in childhood and adolescence: A systematic review and meta-analysis. *World Journal of Psychiatry*, *7*(1), 60-76. <https://doi.org/10.5498/wjp.v7.i1.60>
