# Supplementary material for: The longitudinal course of childhood bullying victimization and associations with self‐injurious thoughts and behaviors in children and young people: A systematic review of the literature
Source: J Adolesc. 2022 Oct 9;95(1):5–33. doi: 10.1002/jad.12097 (PMC10092090; doi:10.1002/jad.12097)
Supplement: Supplementary file 4 — Supporting information. [file JAD-95-5-s001.docx]

Supplementary Table. Main results of the included studies, looking at the associations between bullying and SITBs, and the role of sex/gender

| **Main results of the included studies** | | | | | |
| --- | --- | --- | --- | --- | --- |
| **Author(s), year, study acronym** | **Population: sample size, Mean age (SD, range)** | **Sex/gender**  **(%), SES, ethnicity/ nationality** | **Follow up (time, waves),**  **adjusted for/covariates** | **Main associations, prevalence rates** | **Study findings specific to gender** |
| Bannink et al., 2014  RYM | Baseline (T1), *N =*  8,272  FU (T2), *N =* 3,181  T1, *M =* 12.50, (0.62)  T2, *M =* 14.31 (0.58) | 49% female  Nationality: Dutch (48.4%) | 2 years, 2 waves  Model 1 is adjusted for sociodemographic characteristics (i.e., gender, age, ethnicity, education) and BV  Model 2 is adjusted for Model 1 + suicidal ideation at baseline  Model 3a is adjusted for Model 2 + also includes a Gender x Traditional Bullying Victimisation interaction term  Model 3b is adjusted for Model 2 + also includes a Gender x Cyber Bullying Victimisation interaction term | 1. Traditional BV and suicidal ideation  **Model 1 (*aOR* 1.95, 95% *CI* [1.53, 2.48], *p <* .001)**  **Model 2 (*aOR* 1.56, 95% *CI* [1.21, 2.02], *p <* .001)**  **Model 3a (*aOR* 1.77, 95% *CI* [1.29, 2.44], *p <* .001)**  **Model 3b (*aOR* 1.57, 95% *CI* [1.21, 2.03], *p =* .001)**  2. Cyber BV and suicidal ideation **Model 1 (*aOR* 1.74, 95% *CI* [1.17, 2.61], *p =* .007)** Model 2 (*aOR* 1.22, 95% *CI* [0.80, 1.87], *p =* .36) Model 3a (*aOR* 1.23, 95% *CI* [0.80, 1.89], *p =* .34) Model 3b (*aOR* 1.36, 95% *CI* [0.81, 2.28], *p =* .24)  Prevalence (exposure): 1. Traditional BV victim (21.4%); 2. Cyber BV victim (5.1%)  Prevalence (outcome): Suicidal ideation (11.8% of total sample, *n* = 3181, at follow up) | Prevalence: Traditional BV only (boys: 19.6%, girls: 17.9%)  Cyber BV only (boys: 2.0%, girls: 3.2%)  Both types of bullying (boys: 2.8%, girls: 2.4%)  Suicidal ideation at T2 (**boys: 7.5%, girls: 16.3%, *p <* .001**)  No significant interaction between gender and traditional BV (*aOR* 1.41 95% *CI* [0.83, 2.33], *p =* .20) or cyber BV (*aOR* 1.39 [0.56, 3.45], *p =* .48) on suicidal ideation was found. |
| Benatov et al., 2021 | Baseline (T1), *N =* 5,500  FU (T2), *N =* 2,150  *M =* 15.42 (0.97, 13 – 20 years) | 21.3% female  Arab (52%)  Average or above-average socioeconomic background (92.2%) | 1 year, 2 waves  Model 1 controlling for suicide ideation/attempts at Time 1.  Model 2 additionally controlling for depressive  symptoms at Time 1.  Model 3 additionally controlling for hostility at Time 1.  Model 4 additionally controlling for traditional bullying perpetration | 1. Traditional BV and suicidal ideation  Model 1 (*aOR* 1.26, 95% *CI* [0.83, 1.93], *p =* NR)  Model 2 (*aOR* 1.04, 95% *CI* [0.68, 1.61], *p =* NR)  Model 3 (*aOR* 1.04, 95% *CI* [0.67, 1.60], *p =* NR)  2. Traditional BV and suicide attempts  Model 1 (*aOR* 1.58, 95% *CI* [0.88, 2.81], *p =* NR)  Model 2 (*aOR* 1.16 95% *CI* [0.64, 2.13], *p =* NR)  Model 3 (*aOR* 1.15 95% *CI* [0.63, 2.09], *p =* NR)  3. Cyber BV and suicidal ideation **Model 1 (*aOR* 2.13 95% *CI* [1.27, 3.57], *p <* .05) Model 2 (*aOR* 1.77 95% *CI* [1.04, 2.99], *p <* .05) Model 3 (*aOR* 1.76 95% *CI* [1.04, 2.97], *p <* .05) Model 4 (*aOR* 1.88 95% *CI* [1.08, 3.29], *p <* .05)**  4. Cyber BV and suicide attempts Model 1 (*aOR* 1.67 95% *CI* [0.80, 3.47], *p =* NR) Model 2 (*aOR* 1.28 95% *CI* [0.61, 2.71], *p =* NR) Model 3 (*aOR* 1.28 95% *CI* [0.61, 2.70], *p =* NR)  Model 4 (*aOR* 1.25 95% *CI* [0.58, 2.74], *p =* NR)  Prevalence (exposure): 1. Traditional BV: victims (7.1%, *n* = 153), bully-victims (2.5%, *n* = 50)  2. Cyber BV: victims (3.1%, *n* = 66), bully-victims (1.9%, *n* = 41);  Prevalence (outcome): 1. Suicidal ideation (11.4%, *n* = 243); 2. Suicide attempts (4.6%, *n* = 99) | Not reported |
| Blasco et al., 2019,  UNIVERSAL | Baseline (T1), *N =* 2118  FU (T2), *N =* 1,248  T1, *M =* 18.7 (1.3, 18 – 24)  T2, 67.9% aged 18 years old | T1: 55.4% female  T2: 56.0% female  Nationality: Spanish (country of birth; 95.1%) | 12 months, 2 waves  Multivariable models adjusted by: Age, gender, university, academic field, country of birth, parents’ studies and living location; baseline suicidal ideation (SI) for analyses looking at first-onset of SI | 1. BV and suicidal ideation at 12-month follow up (***OR* 2.40, 95% *CI* [1.55, 3.70], *p <* .01)**  2. BV and suicidal ideation at 12-month follow up (*aOR* 1.51, 95% *CI* [0.80, 2.84], *p =* .20)  3. BV and first-onset suicidal ideation at T2 (***aOR*** **3.2, 95% *CI* [1.08, 9.53], *p =* .04)**  Prevalence (exposure): BV before 17 (32.5%, *SE* 1.37)  Prevalence (outcome): 7.3% (95% CI [5.85-8.77]) reported 12-month SI at T2.  Incidence (outcome): 3.4% (95% *CI* [2.26−4.54]) reported 12-month first-onset of SI at T2 | Not reported – NB has results of gender in a multivariate model (just not looking at association between BV and SITB) |
| Borschmann et al., 2020  CATS | Baseline, *N =* 1,239  FU (T4), *N =* 1,067  T2, T3 (exposure), 9 – 10 years, 10 – 11 years  T4 (outcome), *M =* 11.9 years (0.39, 10.7 - 13.4) | 52.5% female  Nationality: Australia (country of birth, 88.4%)  1st quintile (most advantaged, 35.9%) to 5th quintile (most disadvantaged, 10.7%) | 3 years, 4 waves (waves 2-3 assessed exposure; wave 4 assessed outcome)  Adjusted for age (in years, centred around 12.0 years), sex, and Socio-Economic Index For Areas (SEIFA) advantage/disadvantage quintile | 1. BV reported at one wave before the outcome (i.e., wave 2 *OR* 3) and self-harm (*OR* 6.00, 95% *CI* [0.81, 44.36], *p =* NR)  2. BV reported at one wave before the outcome (i.e., wave 2 *OR* 3) and self-harm (*aOR* 6.78, 95% *CI* [0.94, 49.07], *p =* NR)  3. BV reported at 2 waves before the outcome (i.e., waves 2 AND 3) and self-harm (***OR* 23.05, 95% *CI* [3.53, 150.55], *p =*** NR**)**  4. BV reported at 2 waves before the outcome (i.e., waves 2 AND 3) and self-harm (***aOR* 24.63, 95% *CI* [3.83, 158.21], *p =* NR)**  Prevalence (exposure): BV at one wave (29.7%, *n* = 317), BV at two waves (27.6%, *n* = 294), No BV (42.7%, *n* = 456)  Prevalence (outcome): 3% (out of *n* = 1059) | Prevalence: Self-harm at 11-12 years (boys: 2%, girls: 3%). Girls more likely to self-harm (*OR* 3.2, 95% *CI* [2.1, 5.0], *p =* NR) |
| Brunstein Klomek et al., 2019  SEYLE | T1, *N =* 2,933  T2, *N =* 2,483  T3, *N =* 2,389  T1, *M =* 14.78 (0.89, 13 – 18 years) | T1: 56.1% female  ~10% sample from each of the 10 countries | 12 months, 3 waves (at baseline, 3 months, 12 months)  Gender, age, whether the adolescent was living without his biological parents (yes, no), whether the adolescent  is an immigrant (yes, no), and whether the adolescent’s parents lost their job during the last 12 months (yes, no) were included as covariates to account for their effects (Wasserman et al., 2015).  In the models predicting suicide ideation and/or suicide attempts, depression included as a covariate. | 1. Physical BV (T1) and suicidal ideation/attempts at T2: ***aOR* 2.18 (95% *CI* NR)**, ideation ***p <* .01**; attempts ***p <* .05.**  2. Physical BV (T2) and suicidal ideation/attempts at T3: ideation ***aOR* 2.54 (95% *CI* NR), *p <* .05;** attempts ***aOR* 4.72 (95% *CI* NR), *p <* .001.**  3. Verbal and relational BV did not lead to new cases of suicide ideation and/or attempts at 3 and 12 months (statistics not reported).  4. Physical BV and suicidal ideation at T3  a. Chronic (T1 and T2) physical BV vs. No BV: *aOR* 2.08 (95% *CI* NR), *p =* ns (specific p value NR)  b. Chronic physical BV vs. Sporadic (T1 or T2) BV: *aOR* 0.88 (95% *CI* NR), *p =* ns (specific p value NR)  5. Verbal BV and suicidal ideation at T3  a. Chronic (T1 and T2) verbal BV vs. No BV: *aOR* 0.58 (95% *CI* NR), *p =* ns (specific p value NR)  b. Chronic verbal BV vs. Sporadic (T1 or T2) BV: *aOR* 0.62 (95% *CI* NR), *p =* ns (specific p value NR)  6. Relational BV and suicidal ideation at T3  a. Chronic (T1 and T2) relational BV vs. No BV: ***aOR* 2.63 (95% *CI* NR), *p <* .05**  b. Chronic relational BV vs. Sporadic (T1 or T2) BV: ***aOR* 2.27 (95% *CI* NR), *p <* .05**  7. Physical BV and suicide attempt at T3  a. Chronic (T1 and T2) physical BV vs. No BV: ***aOR* 7.69 (95% *CI* NR), *p <* .01**  b. Chronic physical BV vs. Sporadic (T1 or T2) BV: ***aOR* 3.85 (95% *CI* NR), *p <* .05**  8. Verbal BV and suicide attempt at T3  a. Chronic (T1 and T2) verbal BV vs. No BV: *aOR* 0.51 (95% *CI* NR), *p =* ns (specific *p* value NR)  b. Chronic verbal BV vs. Sporadic (T1 or T2) BV: *aOR* 1.01 (95% *CI* NR), *p =* ns (specific *p* value NR)  9. Relational BV and suicide attempt at T3  a. Chronic (T1 and T2) relational BV vs. No BV: *aOR* 1.10 (95% *CI* NR), *p =* ns (specific *p* value NR)  b. Chronic relational BV vs. Sporadic (T1 or T2) BV: *aOR* 1.19 (95% *CI* NR), *p =* ns (specific *p* value NR)  Prevalence (exposure) 1. Baseline (T1, past 12 months): Relational victimization (8.7%), verbal victimisation (34.6%), relational victimisation (32.3%); 2. (T2, past 3 months): Relational victimization (6.8%), verbal victimisation (23.9%), relational victimisation (23.0%)  Prevalence (outcome): 1. T2 (past 3 months): suicidal ideation (2.3%), suicide attempts (3.1%); 2. T3 (past 12 months): suicidal ideation (2.0%), suicide attempts (2.8%) | Not reported – NB has results of gender in a multivariate model (just not looking at association between BV and SITB) |
| Cho, 2019  KOWEPS | Baseline (Wave 1, T1), *N =* 747  FU (T3, Wave 7), *N =* 512  Students enrolled in either fourth, fifth, or sixth grade at Wave 1 (i.e., ages 9 - 11) | Sex/gender NR | 6 years, 3 waves (Wave 1 (2006), then follow up at Wave 4 (2009) and 7 (2012))  Model 2: mediating negative emotions | Model 1 (multinomial)  1. Direct effects of BV on serious vs. low/no suicidal ideation class membership: **ß = 0.528 (*SE* = 0.236), *OR* 1.696 (95% *CI* NR), *p <* .05**  2. Direct effects of BV on moderate vs. low/no suicidal ideation class membership: ß = 0.28 (*SE* = 0.195), *OR* 1.323 (95% *CI* NR), *p =* ns (specific *p* value NR)  Model 2 (multinomial)  1. Indirect/mediated effects of BV on serious vs. low/no suicidal ideation class membership: ß = 0.327 (*SE* = 0.24), *OR* 1.387 (95% *CI* NR), *p =* ns (specific *p* value NR)  2. Indirect effects of BV on moderate vs. low/no suicidal ideation class membership: ß = 0.324 (*SE* = 0.196), *OR* 1.382 (95% *CI* NR), *p =* ns (specific *p* value NR)  Prevalence: NR | Not measured |
| Cho & Glassner, 2020  KOWEPS | Baseline (T1), *N =* 747  FU (T3, Wave 7), *N =* 542  Students enrolled in either fourth, fifth, or sixth grade at Wave 1 (i.e., ages 9 - 11) | Sex/gender NR  Mean SES = 0.33 (SD = .470), where 0 = general class; 1 = low income class. | 6 years, 3 waves (Wave 1 (2006), then follow up at Wave 4 (2009) and 7 (2012))  Grade, SES, negative emotions (causal steps analysis) | Causal steps analysis:  Model 1: BV on suicidal ideation: ß = 0.100, *SE* = 0.120 , *p =* ns (specific *p* value NR)  Model 4: BV on suicidal ideation (adjusting for negative emotions): ß = 0.014 *SE* = 0.106, *p =* ns (specific *p* value NR)  Coefficient analysis (Indirect Effects of Strains on Suicidal Ideation via Calculating the Product of Coefficients): BV on suicidal ideation, via negative emotions**: ß = 0.089 *SE* = 0.034 (0.023, 0.155), *p <* .01;**  Prevalence: NR | Not measured |
| Copeland et al., 2013  GSMS | Baseline, *N =* 1,420  FU, *N =* 1,273  9, 11 and 13 years at enrolment  Outcomes assessed at 19, 21, or 24-26 years | 49% female  American Indians (25%), African American (8%), Under 1% (Hispanic) | 17 years, 4 – 6 waves between 9 – 16 years, then outcome at 19, 21 or 24 – 26 years  Childhood psychiatric status, family hardships | 1. (Bivariate) BV in childhood and suicidality in young adulthood:  Victims only: *OR* 1.6 95% *CI* [0.7, 4.0], *p =* .29  Bully-victims: ***OR* 5.5 95% *CI* [1.7, 17.4], *p =* .004**  2. BV in childhood and suicidality in young adulthood: *aOR* 1.2, 95% *CI* [0.4, 3.3], *p =* .78  Prevalence (outcome, weighted % by exposure group): victims reported suicidality (9.0%), bully-victims reporting suicidality (24.8%) | Prevalence: Being bullied at least once (boys: 28.8%, girls: 23.4%, *p =* .15)  Victims only (boys: 52.9%, girls: 47.8%, *p =* .34)  **Bully-victims (boys: 72.4%, *p <* .01)**  No significant interaction between sex and BV on suicidality was found for the victim vs neither group.  Significant interactions found for bully-victims, with young men 18.5 times more likely to experience suicidality than males who are not bully-victims: a***OR* 18.5 95% *CI* [6.2, 55.1], *p <* .001**. No association found for young women: a*OR* 0.6 95% *CI* [0.1, 3.9], *p =* .56 |
| Fisher et al., 2012  E-RISK | Baseline, *N =* 2,232  FU, *N =* 2,141  Range: 5 years (confounding questions), 7, 10, 12 years (bullying), 12 years (self-harm) | 51% female | 7 years, data collected at 5, 7, 10 and 12 years old  Model 1: unadjusted  Model 2: adjusted for physical maltreatment by adults  Model 3: adjusted for internalising/externalising problems at age 5  Model 4: adjusted for IQ age 5  Model 5: adjusted for all confounders (physical maltreatment by adults, internalising and externalising problems at age 5, IQ at age 5) | BV in pre-adolescence (mothers’ reports) and self-harm age 12:  Model 1: ***RR* 3.53 95% *CI* [2.10, 5.93]**  Model 2: ***aRR* 2.54 95% *CI* [1.55, 4.16]**  Model 3: ***aRR* 2.43 95% *CI* [1.49, 3.97]**  Model 4: ***aRR* 3.29 95% *CI* [1.95, 5.56]**  Model 5: ***aRR* 1.92 95% *CI* [1.18, 3.12]**  BV in pre-adolescence (child report) and self-harm age 12:  Model 1: ***RR* 3.33 95% *CI* [1.91, 5.82]**  Model 2: ***aRR* 2.71 95% *CI* [1.52, 4.84]**  Model 3: ***aRR* 2.76 95% *CI* [1.60, 4.78]**  Model 4: ***aRR*** **3.02 95% *CI* [1.67, 5.47]**  Model 5**: *aRR* 2.44 95% *CI* [1.36, 4.40]**  Prevalence (exposure): 16.5% (350/2127) reported by mothers as frequently bullied before age of 10; 11.2% (237/2124) reported by children as frequently bullied before age 12.  Prevalence (outcome): 2.9% (62/2141) had self-harmed by age 12 | Prevalence: Bullying (mothers’ reports): Girls: 15.11%, boys: 17.87%  Prevalence: Bullying (child reports): Girls 10.28%, boys: 12.09%  Prevalence: Self-harm (girls: 1.5%, boys: 1.4%)  Exposure to frequent BV before age 12 associated with increased risk of self-harm at age 12, whether BV reported by mothers (**girls: *RR* 2.55 95% *CI* [1.23, 5.28]; boys: *RR* 4.92 95% *CI* [2.33, 10.40**] or by children themselves (**girls: *RR* 4.16 95% *CI* [1.93, 8.95]; boys: *RR* 2.64 95% *CI* [1.18, 5.92**]. |
| Garisch and Wilson, 2016 | T1, *N =* 1,162  T2, *N =* 830  Matched cross-lagged analysis, *N =* 495  T1, *M =* 16.35 (0.62, 16 – 18 years)  T2, *M =* 16.49 (0.71)  Matched cross-lagged data (*M =* 16.23) | 48% female  74.6 % identified themselves as Pākehā, 8.9 % as Māori, and 16.5 % as 'other'.  School deciles ranged from 3 (low) to 10 (high; mean = 7.6, *SD* = 2.54) | 5 months, 2 waves  Anxiety & depressive symptoms;  self-esteem; alexithymia; adaptive  emotional response; resilience;  impulsivity; physical & sexual abuse  history; substance abuse; sexuality  concerns; mindfulness | 1. BV at T1 was not a significant predictor of NSSI at T2 (*r* = 0.12, *p* > .10)  2. Cross-lagged panel correlations of T1 BV and T2 NSSI: (*r* not reported; adjusted *p* not significant, exact *p* value not reported)  Prevalence (exposure): NR  Prevalence (outcome): NSSI at T1 (lifetime): 48.7%; NSSI at T2 (past 3 - 8 month) 34.48% | Prevalence: Lifetime NSSI at T1 (boys: 48%, girls: 49.4%, *p* > .05)  No significant difference between males and females for NSSI at T1, *t(*1337) = .42, *p =* .67. |
| Geoffroy et al., 2021  NLSCY | Baseline, *N =*  8,698  FU, *N =* 2,233  Exposure: 7 – 11 years  Outcome: 12 – 23 years | Sex/gender NR | 11 years, 7 – 11 (Cycle 1) to age 23 or Cycle 8 (whichever came first)  Univariable adjusted for sex  Multivariable associations: 10 risk factors plus sex | 1. Univariable associations (adjusted for sex)  a. BV (Adolescence-limited v. never attempted suicide) *RR* 1.14 95% *CI* [0.40, 3.23], *p =* 0.811  b. BV (Persisting into adulthood v. never attempted suicide) *RR* 2.53 95% *CI* [0.93, 6.93], *p =* 0.071  c. BV (Persisting into adulthood v. adolescence-limited suicide attempt) *RR* 2.23 95% *CI* [0.53, 9.33], *p =* 0.273  2. Multivariable associations  a. BV (Adolescence-limited v. never attempted suicide) *aRR* 0.94 95% *CI* [0.31, 2.49]  b. BV (Persisting into adulthood v. never attempted suicide) *aRR* 1.60 95% *CI* [0.61, 4.23]  c. BV (Persisting into adulthood v. adolescence-limited suicide attempt) *aRR* 1.71 95% *CI* [0.40, 7.29]  Prevalence (outcome): age 13-14 (3.6%), age 14-15 (5.6%), age 22-23 (1%) | Not reported – NB has results of gender in a multivariable model (just not looking at association between BV and SITB) |
| Heikkilä et al., 2013  AMHCS | Baseline (T1), *N =* 3,278  FU (T2), *N =* 2,070  T1, *M =* 15.5 (0.36)  T2, *M =* 17.6 (0.41) | 56.40% female | 2 years, 2 waves  Model 1: involvement in bullying at age 15 was entered after controlling for sex and age  Model 2: depressive symptoms at age 15 were added  Model 3: depressive symptoms removed and externalizing symptoms at age 15 added.  Model 4: both depressive and externalizing symptoms at age 15 are included | 1. BV, victim only, (age 15) and suicidal ideation (age 17):  **Model 1: *aOR* 3.8 95% *CI* [1.8, 8.0], *p <* 0.001**  **Model 2: *aOR* 2.4 95% *CI* [1.1, 5.3], *p =* 0.04**  **Model 3: *aOR* 3.3 95% *CI* [1.5, 7.0], *p =* 0.02**  **Model 4: *aOR* 2.3 95% *CI* [1.0, 5.2], *p =* 0.04**  2. BV, bully-victim, (age 15) and suicidal ideation (age 17):  **Model 1: *aOR* 5.4 95% *CI* [1.5, 20.0], *p =* 0.01**  Model 2: *aOR* 2.0 95% *CI* [0.4, 11.1], *p =* 0.4  Model 3: *aOR* 3.6 95% *CI* [0.9, 13.8], *p =* 0.07  Model 4: *aOR* 1.9 95% *CI* [0.4, 10.7], *p =* 0.5  Prevalence (exposure): 2.6% victims, 0.7% bully-victims  Prevalence (outcome): 8.50% | Not reported – NB has results of gender in a multivariate model (just not looking at association between BV and SITB) |
| Hemphill et al., 2015  IYDS | Baseline (T1), *N =* 805  T2, *N =* 825  T3, *N =*: 791  Sample for analysis (completed data on over 690 participants)  T1, *M =* 15.1 (0.4, 14.1 – 16.5)  T2, *M =* 16.0, SD: 0.4, 15-17.3 years  T3, *M =* 17.0 (0.4, 16.1 – 18.5 years) | 54% female | 2 years, 3 waves  In adjusted models: traditional bullying, individual risk factors (impulsivity, concentration), peer risk factors, family risk factors, school risk factors. All analyses controlled for gender and clustering of schools | 1. Association between CV and self-harm (Grade 9)  (Unadjusted) Victims only: *OR* 1.64 95% *CI* [0.76, 3.50], *p =* NR  Victims only: *aOR* 0.87 95% *CI* [0.36, 2.11], *p =* NR  (Unadjusted) Bully-victims: ***OR*** **2.42 95% *CI* [1.27, 4.61], *p <* .01**  Bully-victims: *aOR* 0.99 95% *CI* [0.46, 2.13], *p =* NR  2. Association between CV and self-harm (Grade 10)  (Unadjusted) Victims only: ***OR* 3.21, 95% *CI* [1.51, 6.81], *p <* .01**  Victims only: *aOR* 2.01, 95% *CI* [0.82, 4.92]  (Unadjusted) Bully-victims: ***OR* 2.74, 95% *CI* [1.43, 5.25], *p <* .01**  Bully-victims: *aOR* 1.26, 95% *CI* [0.59, 2.71]  3. Association between traditional BV (Grade 9) and self-harm (Grade 10)  Victims only: not reported, p > .05.  Bully-victims**: *aOR* 2.40, 95% *CI* [1.34, 4.29], *p <* .01**  4. Association between traditional BV (Grade 10) and self-harm (Grade 11)  Victims only: ***aOR* 1.91, 95% *CI* [1.02, 3.58], *p <* .05**  Bully-victims**: *aOR* 2.16, 95% *CI* [1.16, 4.03], *p <* .05**  Prevalence (exposure): 6–8 % reported victimisation only, and 8–9 % both cyber-bullied others and were cyber-bullied  Prevalence (outcome): Self-harm, grade 11: 8.89% | Not reported |
| Kiekens et al., 2019 | Baseline (T1), *N =* 4,565  FU (T2), *N =* 2, 163  T1, *M =* 18.3 (1.1) | 56.8% female | 2 years, 2 waves  Multivariate model adjusted for parental psychopathology, physical abuse, emotional abuse, sexual abuse, neglect, dating violence | 1. BV and sporadic NSSI (i.e., 1 – 4 times):  (Bivariate) ***OR* 1.9, 95% *CI* [1.3, 2.6]**  (Multivariate) ***aOR* 1.6, 95% *CI* [1.1, 2.5]**  2. BV and repetitive NSSI (i.e., > 5 times):  (Bivariate) ***OR* 2.1, 95% *CI* [1.5, 3.0]**  (Multivariate) ***aOR* 1.6 95% *CI* [1.0, 2.6]**  Prevalence (exposure): Of those without a history of NSSI at baseline (26%, *SE* = 0.8)  Prevalence (outcome): 12-month incidence of NSSI (Year 1: 10.3%, *SE* = 0.8; Year 2: 6.0%, *SE* = 0.7). Aggregated rates NSSI onset across years 1 and 2 (15.6%, *SE* = 0.9, with 8.6% [*SE* = 0.8] reporting sporadic NSSI and  7.0% [*SE* = 0.6] reporting repetitive NSSI) | Not reported – NB has results of gender in a multivariate model (just not looking at association between BV and NSSI) |
| Kim et al., 2009 | Baseline (T1), *N =* 1,719  FU (T2), *N =* 1,666  T1, 7^th^ grade, *M =* 13.10 (0.32); 8^th^ grade, *M =* 14.11 (0.31) | 44.80% female  High (1.7%), Middle high (17.0%), Middle (67.2%), Middle Low (9.9%), Low (0.8%) SES | 10 months, 2 waves  (1) socio-demographic risk factors:  sex, family structure, parental educational level and SES; (2) psychopathological risk factors at baseline: anxious/factors at baseline: depression, conduct problems and aggression; and, (3) past suicide history (suicidality at baseline). | 1. BV and suicidal behaviours over 6 months (BOYS):  Victim: a. Transient (victim at T1 but not T2): *aOR* 1.36, 95% *CI* [0.48, 3.86];  b. Persistent (victim at T1 and T2): *aOR* 1.94, 95% *CI* [0.77, 4.91]  Victim-bully: a. Transient *aOR* 1.01, 95% *CI* [0.13, 8.17];  Persistent: *aOR* 1.99, 95% *CI* [0.50, 7.98]  2. BV and suicidal behaviours over 6 months (GIRLS):  Victim: a. Transient *aOR* 2.21, 95% *CI* [0.74, 6.55]; b. Persistent: *aOR* 1.81, 95% *CI* [0.48, 6.79]  Victim-bully: a. Transient *aOR* 0.53, 95% *CI* [0.04, 6.75]; b. Persistent: *aOR* 4.94, 95% *CI* [0.86, 28.33]  3. Ideations over 6 months (BOYS):  Victim: a. Transient: *aOR* 1.70 95% *CI* [0.75, 3.84]; b. Persistent *aOR* 2.13, 95% *CI* [0.95, 4.78]  Victim-bully: a. Transient *aOR* 3.30, 95% *CI* [0.98, 11.14]; b. Persistent *aOR* 0.76, 95% *CI* [0.20, 2.84]  4. Ideations over 6 months (GIRLS):  Victim: a. Transient *aOR* 1.78, 95% *CI* [0.81, 3.90]; b. Persistent *aOR* 1.59, 95% *CI* [0.63, 4.06]  Victim-bully: a. Transient *aOR* 1.73, 95% *CI* [0.31, 9.72]; b. Persistent *aOR* 0.52, 95% *CI* [0.11, 2.49]  5. Ideations over 2 weeks (BOYS):  Victim: a. Transient *aOR* 0.94, 95% *CI* [0.41, 2.16]; b. Persistent *aOR* 1.74, 95% *CI* [0.75, 4.05]  **Victim-bully: a. Transient *aOR* 6.39, [1.58, 25.88] *p <* .01**; b. Persistent *aOR* 0.80, 95% *CI* [0.19, 3.30];  6. Ideations over 2 weeks (GIRLS):  Victim: a. Transient *aOR* 1.23, 95% *CI* [0.50, 3.02]; b. Persistent *aOR* 2.29, 95% *CI* [0.62, 8.48]  Victim-bully: a. Transient *aOR* 3.44, 95% *CI* [0.52, 22.87]; b. Persistent *aOR* 2.48, 95% *CI* [0.54, 11.35]  Prevalence (exposure): Victims only (14%), Bully-victims (9%)  Prevalence (outcome): a. Suicidal behaviours (boys: 9.5%, girls: 13.4%, *p <* .01); b. Suicidal ideations over 6 months (boys: 24.38%, girls 41.59%, *p <* .001); c. Suicidal ideations over two weeks (boys: 35.33%, girls: 50.55%, *p <* .001). | |
| Klomek et al., 2008 | Baseline (T1), *N =* 2,946  FU (T2), *N =* 2,348  T1, *M =* 8 years  T2, *M =* 18 years | 0% female (100% male) | 10 years, 2 waves  Depression at age 8 | 1. (Unadjusted) Association between experiencing BV sometimes and suicidal ideation *OR* 1.3, 95% *CI* [0.96, 1.7]  2. (Unadjusted) Association between experiencing BV frequently and suicidal ideation *OR* 1.2, 95% *CI* [0.8, 2.0]  Adjusted results not reported (not statistically significant)  Prevalence (exposure): 56.2% (sometimes, 47.6%, frequently, 8.6%)  Prevalence (outcome): 12.7% | N/A |
| Klomek et al., 2009  EMCPS | Baseline, *N =* 5,813  FU, *N =* 5,302  T1, *M =* 8 years (1989);  FU = 16 years later (2005) | 49% female | 16 years  Baseline conduct symptoms (based on the parent’s Rutter conduct scale) and/or baseline depression (based on the CDI). | 1. "Frequent" BV and suicide attempt/death by suicide (BOYS):  a. **Unadjusted *OR* 6.5, 95% *CI* [2.1, 20.7], *p <* .01)**  b. **Adjusted for depression at baseline *OR* 7.7, 95% *CI* [2.2, 26.9], *p <* .01)**  c. **Adjusted for conduct disorder at baseline *OR* 3.5, 95% *CI* [1.02, 12.0], *p <* .05)**  d. Adjusted for conduct disorder and depression at baseline *OR* 3.8, 95% *CI* [0.99, 14.3]  2. "Frequent" BV and suicide attempt/death by suicide (GIRLS):  a. **Unadjusted *OR* 4.2, 95% *CI* [1.2, 15.0], *p <* .05**  b. **Adjusted for depression at baseline *OR* 5.3, 95% *CI* [1.3, 21.0], *p <* .05**  c. **Adjusted for conduct disorder at baseline *OR* 4.3, 95% *CI* [1.1, 16.1], *p <* .05**  d. **Adjusted for conduct disorder and depression at baseline *OR* 6.3, 95% *CI* [1.5, 25.9], *p <* .05.**  3. "Sometimes" BV and suicide attempt/death by suicide (BOYS):  a. Unadjusted *OR* 2.5, 95% *CI* [0.9, 7.0]  b. Adjusted for depression at baseline *OR* 2.7, 95% *CI* [0.9, 7.5]  c. Adjusted for conduct disorder at baseline *OR* 1.9, 95% *CI* [0.7, 5.4]  d. Adjusted for conduct disorder and depression at baseline *OR* 1.9, 95% *CI* [0.7, 5.5]  4. "Sometimes" BV and suicide attempt/death by suicide (GIRLS):  a. Unadjusted *OR* 1.3, 95% *CI* [0.5, 3.0]  b. Adjusted for depression at baseline *OR* 1.4, 95% *CI* [0.6, 3.4]  c. Adjusted for conduct disorder at baseline *OR* 1.3, 95% *CI* [0.5, 3.1]  d. Adjusted for conduct disorder and depression at baseline *OR* 1.5, 95% *CI* [0.6, 3.7]  Prevalence: Frequent BV (boys: 9.4%, girls: 3.7%); Sometimes BV (boys: 47.8%, girls: 36.1%); Frequent BV and suicide attempt/death by suicide (boys: 2.9%, girls: 3.3%); Sometimes BV and suicide attempt/death by suicide (boys: 1.2%, girls: 1.0%) | |
| Le et al., 2017 | Baseline (T1), *N =* 1,539  FU (T2), *N =* 1,424  *M =* 14.7 (1.9, 12-17) | 54.90% female | 6 months, 2 waves  Adjusted models controlled for confounders measured at Time 1 including: age, depression (for model of depression), psychological distress (for model of psychological distress), suicidal ideation (for model of suicidal ideation), self-esteem, average time spending on online, family social support, school social support, friend social support, witness parental violence, and conflict with siblings | 1. (Bivariate) BV (‘declining’ – reported at Time 1 but not T2) and suicidal ideation at T2:  a. **Victims only**: ***OR* 2.8, 95% *CI* [1.3, 6.0], *p <*.01**  **b. Bully-victims: *OR* 3.2 (1.7;6.0) *p <* .001**  2. (Multivariate) BV (‘declining’) and SI at T2:  a. Victims only: ***aOR* 2.5, 95% *CI* [1.1, 5.5], *p <* .05**  b. Bully-victims: *aOR* 1.7, 95% *CI* [−0.3, 3.8], *p =* ns  3. (Bivariate) BV (‘stable-high’ – reported at T1 and T2) and suicidal ideation at T2:  **a. Victims only: *OR* 3.8, 95% *CI* [1.6, 9.1], *p <* .05**  **b. Bully-victims: *OR* 6.6, 95% *CI* [3.5, 12.5], *p <* .001**  4. (Multivariate) BV (‘stable-high’) and suicidal ideation at T2:  a. Victims only: ***aOR* 3.1, 95% *CI* [1.2, 8.1], *p <* .05**  b. Bully-victims: ***aOR* 3.8, 95% *CI*** [**1.4, 6.2), *p <* .01**  Prevalence (exposure): a. Victim: traditional and cyber (3.8%), traditional (18%), cyber (0.9%);  b. Bully-victim: traditional and cyber (8.9%), traditional (13.1%), cyber (0%)  Prevalence (outcome): NR | Prevalence: Victims only (boys: 24.9%, girls: 23.3%)  Bully-victims (boys: 34.7%, girls: 27.0%)  Suicidal ideation at T2 (boys: 12.2%, girls: 13.4%, *p =* ns)  1. (Bivariate), BV (‘declining’) Victims only and SI: Boys *OR* 1.6 [0.5, 5.0], *p =* ns; **Girls *OR* 4.7, 95% *CI* [1.7, 13.1], *p <* .01;**  Bully-Victims and SI: Boys 1.6 [0.6, 4.7], *p =* ns; **Girls *OR* 5.1, 95% *CI* [2.2, 11.5] *p <* .001**;  2. (Multivariate), BV (‘declining’) Victims only and SI: Boys *aOR* 1.7, 95% *CI* [0.5, 5.8], *p =* ns**; Girls *aOR* 4.1 95% *CI* [1.4, 12.0], *p <* .05;**  Bully-victims and SI: Boys *aOR* 2.8 95% *CI* [0.9, 8.3], *p =* ns; **Girls *aOR* 3.9, 95% *CI* [1.6, 9.6], *p <* .01**  3. (Bivariate), BV (‘stable-high’) Victims only and SI: Boys *OR* 2.8, 95% *CI* [0.9, 8.3], *p =* ns; **Girls *OR* 5.9, 95% *CI* [1.4, 24.0], *p <* .05**  Bully-Victims and SI: Boys ***OR* 4.8, 95% *CI* [2.0, 11.8], *p <* .001**; Girls ***OR* 8.9, 95% *CI* [3.5, 22.5], *p <* .001**  4. (Multivariate), BV (‘stable-high’) Victims only and SI: Boys *aOR* 2.7, 95% *CI* [0.8, 9.1], *p =* ns; Girls *aOR* 3.3, 95% *CI* [0.6, 17.3], *p =* ns  Bully-victims and SI: Boys *aOR* **3.5, 95% *CI* [1.2, 9.9], *p <* .001**; Girls *aOR* **6.5, 95% *CI* [2.2, 19.5], *p <* .001** |
| Le et al., 2019 | Baseline (T1), *N =* 1,539  FU (T2), *N =* 1,167  *M =*  13.71 (1.89, 11-16) | 55% female | 6 months, 2 waves  Demographics (gender, age in years, family structure), family, friend, and school social support (perception of students and teachers helping to stop bullying), witness parental violence, conflict with siblings, time spent on online, previous suicidal ideation | BV at T1 and SI at T2 for victims: ***aOR* 2.02, 95% *CI* [1.33, 3.06], *p <* .001**  BV at T1 and SI at T2 for bully-victims: ***aOR* 1.83, 95% *CI* [1.01, 3.32], *p <* .05**  Prevalence (exposure): a. Victim (26.19%); b. Bully-victim (9.41%)  Prevalence (outcome): 12.85% | Prevalence: Victims only (boys: 31.78%, girls: 21.61%)  Bully-victims (boys: 10.90%, girls: 8.18%)  Suicidal ideation at T2 (boys: 12.15%, girls: 13.43%, *p =* ns)  1. Girls (BV at T1 on SI at T2**)**  **a. Victims only: *aOR* 2.12 (1.21, 3.72), *p <* .01; b. Bully-victims: *aOR* 2.30 [1.07, 4.92], *p <* .05**  2. Boys (BV at T1 on SI at T2)  a. Victims only: *aOR* 1.88 [0.98, 3.60], *p =* ns;  b. Bully-victims: *aOR* 1.32 [0.54, 3.23], *p =* ns |
| Lereya et al., 2013  ALSPAC | Baseline, NR  FU, *N =* 4,810  Exposure collected between 7 and 10 years.  Outcome collected aged 16 – 17 (*M =* 16.7, *SD* = 0.2) | 59% female  White 96.1% | 6 to 10 years, multiple time points  Sex, preschool maladaptive parenting, preschool domestic violence, internalising/externalising behaviour, BPD symptoms, depression symptoms | Crude and adjusted associations between stable BV (i.e., BV at two time points) or unstable BV (i.e., BV at one time point) and SH:  1. Model A (bivariate)  a. Child report (stable), ***OR* 1.78, 95% *CI* [1.38, 2.30]**  b. Child report (unstable**) *OR* 1.43 [1.20, 1.71]**  c. Mother report (stable) ***OR* 1.79 [1.45, 2.21]**  d. Mother report (unstable) *OR* 1.18 [0.97, 1.43]  e. Teacher report (stable) ***OR* 2.68 [1.41, 5.11]**  f. Teacher report (unstable) ***OR* 1.52 [1.19, 1.94]**  2. Model B (adjusted for sex, preschool domestic violence, preschool maladaptive parenting, and internalizing/externalizing behavior)  a. Child report (stable) ***aOR* 1.79, 95% *CI* [1.34, 2.41]**  b. Child report (unstable**) *aOR* 1.44 [1.17, 1.77]**  c. Mother report (stable**) *aOR* 1.64 [1.28, 2.11]**  d. Mother report (unstable) *aOR* 1.19 [0.95, 1.50]  **e. Teacher report (stable) *aOR* 3.50 [1.46, 8.42]**  f. Teacher report (unstable) *aOR* 1.24 [0.90, 1.70]  3. Model C (controlling for borderline personality disorder symptoms and depression symptoms in addition to sex, preschool domestic violence, preschool maladaptive parenting, and internalizing/externalizing behavior)  a. Child report (stable) *aOR* **1.66, 95% *CI* [1.20, 2.31]**  **b**. Child report (unstable**) *aOR* 1.38 [1.10, 1.74]**  c. Mother report (stable**) *aOR* 1.59 [1.19, 2.13]**  d. Mother report (unstable) *aOR* 1.13 [0.87, 1.47]  e. Teacher report (stable) ***aOR* 4.75 [1.72, 13.07]**  f. Teacher report (unstable) *aOR* 1.39 [0.97, 2.00]  Path analysis found bullying indirectly increased self harm through the development of depressive symptoms **(*B =* 0.07; *SE* = 0.02; *p <* .001).**  Prevalence (exposure): a. Child report at 8 years (38%); b. Child report at 10 years (22.9%); c. Mother report at 7 years (16%); d. Mother report at 8 years (20.5%); e. Mother report at 9 years (21.5%); e. Teacher report at 7 years (8.7%); f. Teacher report at 10 years (12.3%).  Prevalence (outcome): 16.5% (*N =* 792) | Prevalence: self-harm in 12 months (boys: *n* = 162 [3.38%], girls: *n* = 630 [13.13%])  Prevalence: lifetime self-harm (boys: *n* = 180 [3.74%], girls: *n* = 725 [15.06%])  Boys were significantly more likely to be bullied, and the association between the sex of the child and self-harm via being bullied was stronger for boys **(*B =* -0.04, *SE* = 0.01, *p =* .001);** overall, girls were more likely to engage in self-harm and the indirect association between child sex and self-harm via depression symptoms was significantly stronger for girls **(*B =* 0.07, *SE* = 0.02, *p <* .001).** |
| Lereya et al., 2015  ALSPAC and GSMS | Baseline, NR  FU, *N =* 4,026 (ALSPAC),  *N =* 1,420 (GSMS)  ALSPAC: T1 collected at 8 years, outcome at 18 years  GSMS: T1 collected at 9 years, outcome at 19, 21 or 24 – 26 years | ALSPAC: 56% female  GSMS: 49% female | ALSPAC: 5 to 10 years, multiple time points  GSMS: 3 to 17 years, multiple time points  For ALSPAC: adjusted for sex, family adversity during pregnancy and any prenatal maternal mental health problems (anxiety and/or depression)  For GSMS: adjusted for sex, socioeconomic status, family instability and family dysfunction; and percentages are weighted; sample sizes are unweighted | 1. (Bivariate) Association between BV and self-harm / suicidality  ALSPAC:  **Being bullied only v not bullied: *OR* 1.8, 95% *CI*, [1.4, 2.3], *p <* 0.0001**  **Being bullied and maltreated v not bullied or maltreated *OR* 2.0 [1.4, 3.0], *p =* 0.002**  GSMS:  **Being bullied only v not bullied: *OR* 3.0, 95% *CI* [1.2, 8.0], *p =* 0.02**  Being bullied and maltreated v not bullied or maltreated: *OR* 2.2 [0.7, 6.9], *p =* 0.19  2. (Adjusted results) Association between BV and self-harm / suicidality  ALSPAC:  **Being bullied only v not bullied: *aOR* 1.7 [1.4, 2.2], *p <* 0.0001**  **Being bullied and maltreated v not bullied or maltreated: *aOR* 1.8 [1.2, 2.8], *p =* 0.003**  GSMS:  **Being bullied only v not bullied: *aOR* 3.0 [1.2, 7.7], *p =* 0.02**  Being bullied and maltreated v not bullied or maltreated: *aOR* 2.2 [0.6, 7.7], *p =* 0.24  Prevalence (exposure): a. ALSPAC: 30% exposed to only bullying, 7% exposed to maltreatment and bullying (at least once age 8, 10 or 13);  b. GSMS: 225 (16%) exposed to only bullying, and 159 (10%) to both maltreatment and bullying (at least once age 9-16)  Prevalence (outcome): ALSPAC: 9% reported self-harm in the past year; GSMS: 7% reported self-harm in the past year | Not reported  NB. Looks at Phi correlations between sex and bullying. |
| Lung et al., 2020  TBCS-P | Baseline (T1), *N =* 1,561  FU (T2), *N =* 1,457  Exposure collected at 12 years, outcome collected at 13 years | 45.70% female | 1 year, 2 waves  None | **Being bullied at age 12 years increased likelihood of self-harm at 13 years old (β = 0.10, *p <* 0.001)**  Prevalence (exposure): 24.3%, past year  Prevalence (outcome): 5.4%, past year | Not reported |
| Mars et al., 2020  ALSPAC | Baseline (T1), *N =* 1,496  FU (T2), *N =* 1,431  T1, *M =* 18.2 (6 months)  T2, *M =* 20.8 | 62.50% female  White 96% | 3 years, 2 waves  Socioeconomic position (SEP), previous mental health problems, total numbers of hours spent online | 1. (Unadjusted) Association between CV and self-harm:  Males: *OR* 1.75, 95% *CI* [0.39, 7.77]  Females: ***OR* 3.01 [1.82, 4.96]**  2. (Adjusted for socioeconomic position (SEP) and hours online) Association between CV and self-harm:  Males: *OR* 1.55, 95% *CI* [0.35, 6.93]  Females: ***OR* 2.96 [1.78, 4.92]**  3. (Adjusted for SEP, hours online and previous mental health problems) Association between CV and self-harm:  Males: *OR* 1.59, 95% *CI* [0.35, 7.26]  Females: ***OR* 2.42 [1.41, 4.15]**  Prevalence (exposure): NR  Prevalence (outcome): Self-harm in the past 12 months (males: 10%, females: 13%) | |
| Mortier et al., 2017  LCS | Baseline (T1), *N =* 2,337  FU (Wave 2 or 3), *N =* 1,253  T1, *M =* 18.43 (0.96)  T3, *M*= 20.27 (0.80) | 55.60% female  Nationality: Non-Belgian (8.1%)  Parents' financial situation difficult 16.0% | 2 years, 3 waves (FU survey at 12 and 24 months)  Analyses only included those without history of STB at baseline. All analyses were adjusted for sociodemographics (i.e., gender, age, nationality, familial composition and socio-economic status, sexual orientation, university group membership, and living situation), and all other risk factors shown in the table (i.e., abuse, neglect, dating violence, 12-month mental disorders, 12-month stressful experiences) | 1. (Bivariate) BV before 17 and suicidal ideation *OR* 1.38, 95% *CI* [0.80, 2.38], PARP 9.5%  2. (Bivariate) BV before 17 and suicidal plan *OR* 1.71 [0.98, 2.99], PARP 16.7%  Prevalence (exposure): 29.8%  Prevalence (outcome): Among students without STB at baseline (*N =* 2,042):  a. Suicidal ideation at 12 month FU (3.7%, *SE* = 0.6); at 24 month FU (3.9%, *SE* = 0.6);  b. Suicide plans at 12 month FU (0.9%, *SE* = 0.2); at 24 month FU (2.2%, *SE* = 0.5);  c. Suicide attempts were estimated at 0.2% (*SE* = 0.1) in both year 1 and 2.  d. Aggregated one-year incidence proportions for any form of first-onset STB (i.e., at least suicidal ideation) were 4.8% (*SE* = 0.7) in year 1, and 6.4% (*SE* = 0.9) in year 2. | Not reported |
| O’Connor et al., 2009  CASE | Baseline (T1), *N =* 737  FU (T2), *N =* 500  *M =* 15.2 (0.72, 15 – 16) | 47% female | 6 months, 2 waves  None | 1. BV and self-harm (first time, at T2) unadjusted *OR* 1.06, 95% *CI* [0.32, 3.49], *p =* .924  2. BV and self-harm (repetition, at T1 and T2) unadjusted ***OR* 2.98 [1.15, 7.71], *p =* .024**  Prevalence (exposure): 31% (lifetime prevalence)  Prevalence (outcome): a. Baseline (9.5%, 12 month prevalence);  b. Self-harm between T1 and T2 (6.2%) | Prevalence: Self-harm between T1 and T2 (boys: 2.2%, girls: 4.0%)  Prevalence: Girls more likely to repeat self-harm at T1 *and* T2 (*OR* 5.81, [1.66, 20.32]) |
| Özdemir and Stattin, 2011  SSSLS | Baseline, *N =* 508  FU, *N =* 463  T1, *M =* 13.2 (0.61)  T2, *M =* 14 (0.75)  T3, *M =* 14.98 (0.73) | 53.3% female | 3 years, 3 waves (annually, spring term)  Age, gender | 1. Association between BV at T1 and self-harm behaviours at T2:  Victims only: **β = 0.15, *p <* 0.05**  Bully-victims: **β = 0.21, *p <* 0.05**  2. Association between BV at T2 and self-harm behaviours at T3:  Victims only: **β = 0.14, *p <* 0.05**  Bully-victims: **β = 0.13, *p <* 0.05**  3. Whether BV predicted changes in SH at T2 (compared to T1):  Victims only: **β = 0.10, *p <* 0.05**  Bully-victims: **β = 0.15, *p <* 0.05**  4. Whether BV predicted changes in SH at T3 (compared to T2):  Victims only: β = 0.06, *p =* ns  Bully-victims: β = 0.03, *p =* ns  Prevalence (exposure): Overall (T1: 35%, last semester); Bully-victims (21%); Victims only (16%)  Prevalence (outcome): Not reported | The effects of BV remained the same after controlling for gender |
| Perret et al., 2020  QLSCD | Birth, *N =* 2,120  FU, *N =* 1,160 – 1,192  T1 = 13 years  T2 = 15 years  T3 = 17 years | T1/T2/T3 = 62.5% / 61.1% / 63.0% female | Taken from birth cohort study; Data in this paper covers 4 years total, 3 waves (data taken every 2 years, analyses at two year intervals: 13 to 15; 15 to 17)  Model 1 adjusted for sex.  Model 2 additionally adjusted for prior family socioeconomic status (6 – 12 years), family structure (12 years), family functioning (6 – 12 years), hostile-reactive parenting (6 – 12 years), depressive symptoms (6 – 12 years), anxiety (10 – 12 years), oppositional-defiant symptoms (6 – 12 years) and inattention/hyperactivity symptoms (6 – 12 years).  Model 3 additionally adjusted for face-to-face victimization or cybervictimization at each given age.  Model 4 additionally adjusted for suicidal ideation and attempt at baseline | 1. Face-to-face BV 13 years & suicide ideation/attempt at 15:  **Model 1: *aOR* 4.26, 95% *CI* [3.34, 5.43]**  **Model 2: *aOR* 3.08 [2.36, 4.03]**  **Model 3: *aOR* 2.78 [2.11, 3.67]**  **Model 4 *aOR* 2.45 [1.82, 3.29]**  2. Face-to-face BV at 15 years & suicide ideation/attempt at 17:  **Model 1: *aOR* 2.95 [2.33, 3.73]**  **Model 2: *aOR* 2.50 [1.94, 3.23]**  **Model 3: *aOR* 2.26 [1.72, 2.97]**  **Model 4: *aOR* 2.06 [1.56, 2.72]**  3. Cyber BV at 13 years & suicide ideation/attempt at 15:  **Model 1: *aOR* 3.02 [2.28, 4.00]**  **Model 2: *aOR* 2.30 [1.70, 3.12]**  **Model 3: *aOR* 1.79 [1.30, 2.44]**  Model 4: *aOR* 1.37 [0.97, 1.93]  4. Cyber BV at 15 years & suicide ideation/attempt at 17:  **Model 1: *aOR* 2.23 [1.74, 2.86]**  **Model 2: *aOR* 1.82 [1.40, 2.37]**  **Model 3: aOR1.34 [1.01, 1.78]**  Model 4: *aOR* 0.98 [0.73, 1.33]  Prevalence (exposure): a. Cybervictimization: 12 years (6.8%), 13 years (9.8%), 15 years (16.0%), 17 years (7.2%); b. Face-to-face NR  Prevalence (outcome): Overall, prevalence of suicidal ideation/attempt increased from 3.4% (*n* = 42), 3.5% (*n* = 42) and 5.8% (*n* = 62) for those never cybervictimized at 13, 15 and 17 years, respectively, to 13.3% (*n* = 14), 19.3% (*n* = 44) and 25.5% (*n* = 22) for those exposed to cybervictimization in given school year. | Prevalence: CV at 12 years (boys: 6.4%, girls: 7.1%, *p =* .600)  **CV at 13 years (boys: 6.8%, girls: 11.6%, *p =* .005)**  **CV at 15 years (boys: 10.7%, girls: 19.3%, *p <* .001)**  **CV at 17 years (boys: 5.3%, girls: 8.3%, *p =* .044)**  No significant sex-by-cybervictimization interaction was found (*p* > .05) |
| Quintana-Orts et al., 2022 | Baseline (T1), *N =* 1,001  FU (T2), *N =* 835  T1, *M =* 13.77 (1.34, 12 – 18 years)  T2, *M =* 13.71 (1.31, 12 – 18 years) | T1 = 54.1% female  Nationality: Spanish (96.9% T1, 97.9% T2) | 4 months, 2 waves  Covariates: Gender, age and grade  Moderator: Core self-evaluation | 1. Cybervictimization and suicidal ideation at T2**: ß = 0.38, *p <* 0.001** 2. Moderation analysis: CV x core self-evaluation (CSE) explained a medium (f^2^ = 0.012) additional amount of variance in T2 suicide ideation. Overall 29% of variance was explained by the full model **(r^2^ = 0.28, F (6,828) = 53.79, *p <* 0.001).** 3. Significant positive association between cybervictimization and suicide ideation at low levels of CSE **(ß = 0.60, t(828) = 7.70, *p <* 0.001**), which was weaker at mean levels of CSE (**ß = 0.38, t(851) = 4.82, *p <* 0.001).** At high levels of CSE, the relationship between cybervictimization and suicide ideation was not significant (ß = 0.15, t(851) = 1.29, *p =* 0.20).   Prevalence (exposure): Cybervictimisation at T1 (0.27, SD = 0.43), range 0 – 3  Prevalence (outcome): Suicidal ideation at T2 (1.63, SD = 0.91), range 1 - 5 | No gender differences were found for cybervictimization scores (t (902.01) = 0.23, *p =* 0.82; *M =* 0.27, SD = 0.46 for boys and *M =* 0.26, SD = 0.39 for girls)  Gender significantly predicted T2 suicide ideation **(ß = 0.19, *p <* 0.001).** Girls scored higher in suicide ideation than boys in T2 **(t(831.09) = - 5.30, *p <* 0.001**; *M =* 1.46, SD = 0.76 for boys and *M =* 1.78, SD = 0.99 for girls). |
| Sigurdson et al., 2018  YMHS | Baseline (T1), *N =* 2,464  FU (Time 2), *N =* 2,432  FU (Time 4), *N =* 2,532  T1, *M =* 13.7 (0.58, 12.5 - 15.7 years)  T2, *M =* 14.9 (0.59, 13.7 - 17.0)  T4, *M =* 27.2 (0.59, 26.0 - 28.2) | T1/T2/T4 = 50.8% / 50.4% / 56.7% female  Place of parents’ birth: Norway (93.4%), one parent outside Norway (3.9%), Eastern Europe or Third World country (2.7%)  Parental occupation (SES proxy): Professional leader (9.8%), Upper middle class (28.6%), Lower middle class (13.2%), primary industry (8.3%), manual worker (36.5%), missing (3.7%) | 14 years (T1, 1998; T2, 1999; T4, 2012), 3 waves with 14 year gap - T3 data not used  Parents' SES, gender, time points and bullied status | 1. Associations between BV and suicidal ideation (T2)  a. Females ***aOR* 2.37, 95% *CI* [1.61, 3.47], *p <* .001**  b. Males ***aOR* 3.63 [2.37, 5.57], *p <* .001**  2. Associations between BV and suicidal ideation (T4)  a. Females ***aOR* 2.68 [1.52, 4.73], *p <* .001**  b. Males *aOR* 1.76 [0.89, 3.49], *p =* .103  3. Associations between BV and self-harm (T2)  a. Females ***aOR* 3.30 [2.07, 5.26], *p <* .001**  b. Males ***aOR* 4.62 [2.47, 8.67], *p <* .001**  4. Associations between BV and self-harm (T4)  a. Females ***aOR* 1.91 [1.01, 3.63], *p =* .047**  b. Males ***aOR* 3.86 [1.31, 11.41], *p*= .014**  5. Associations between BV and suicide attempts (T2)  a. Females ***aOR* 3.90, 95% *CI* [2.26, 6.73], *p <* .001**  b. Males ***aOR* 6.26 [2.94, 13.30], *p <* .001**  6. Associations between BV and suicide attempts (T4)  a. Females *aOR* 1.30 [0.49, 3.45], *p =* .600  b. Males ***aOR* 6.06 [2.25, 16.36], *p <* .001**  Prevalence (exposure): BV (males: 9.7%, females: 10.0%, *p =* .784); Teasing (males: 7.7%, females: 8.7%); Physical assault (males: 2.8%, females: 1.4%); Exclusion (males: 3.3%, females: 3.9%)  Prevalence (outcome): a. Suicidal ideation at T2 (boys: 16.83%, girls: 32.55%);  b. SI at T4 (males: 19.83%, females: 15.66%);  c. Self-harm at T2 (boys: 5.37%, girls: 12.48%); SH at T4 (males: 4.28%, females: 14.24%); Suicide attempts at T2 (boys: 3.01%, girls: 7.26%); SA at T4 (males: 4.30%, females: 6.52%) | |
| Silberg et al., 2016  VTSABD and YAFU | Baseline, *N =* 2,824  FU, *N =* 2,307  VTSABD: Range = 8 – 17 years  YAFU: *M =* 23 years | Sex/gender NR | VTSABD T1 data collected 1980-85, every 2 years; YAFU collected when children 18+ (young adults only)  Not reported | (Bivariate) Association between BV in childhood and suicidal ideation as young adult in bullied v non-bullied twins: ***OR* 1.9, 95% *CI* [1.3, 3.0], *p <* .01**  (Bivariate) MZ-discordant twins ***OR* 2.9 [1.2, 7.2], *p <* .01**  Prevalence (exposure): Not reported  Prevalence (outcome): 11% experienced suicidal ideation among bullied twins, 6% experienced suicidal ideation among non-bullied twins | Not reported |
| Sourander et al., 2006  FCC | Baseline, *N =*  907 parents, 900 children  FU, *N =* 738 parents, 839 children  T1 = 12 years  T2 = 15 years | 52.8% female | 3 years, 2 waves  Univariate: Gender;  Multivariate, Model 1: Female sex, mother's health problems, self-reports of deliberate self-harm, nonintact family structure, CBCL total scores, learning difficulties, bullied  Model 2: Female sex, mother's health problems, self-reports of deliberate self-harm, nonintact family structure, CBCL externalising, learning difficulties, YSR internalising, bullied  Model 3: Female sex, mother's health problems, CBCL aggressivity, self-reports of deliberate self-harm, nonintact family structure, YSR somatic complaints, learning difficulties, bullied | 1. Association between BV and self-harm ideation only (controlling for gender only) ***aOR* 5.1, 95% *CI* [2.0, 13.1]**  2. Association between BV and self-harm acts only (controlling for gender only) *aOR* 0.96 [0.3, 2.8]  3. (Multivariate) Model 1 (including CBCL/YSR total scores):  a. BV and self-harm ideations only ***aOR* 3.4 (1.2*,* 9.6)**  b. BV and self-harm acts only *aOR* 0.5, 95% *CI* [0.1, 1.7]  4. (Multivariate) Model 2 (including CBCL/YSR sub scores):  a. BV and self-harm ideations only *aOR* **3.8 (1.3*,* 10.8)**  b. BV and self-harm acts only *aOR* 0.5, 95% *CI* [0.1, 1.9]  5. (Multivariate) Model 3 (including CBCL/YSR syndrome scales):  a. BV and self-harm ideations only *aOR* **4.0 (1.4*,* 11.4)**  b. BV and self-harm acts only *aOR* 0.6 [0.2, 2.2]  Prevalence (exposure): 10.30%  Prevalence (outcome): See right | Prevalence: self-harm ideation or acts (child report) at age 12: Girls, 2.7%; Boys, 3.1%  Prevalence: self-harm ideation or acts (child report) at age 15: Girls, 12.6%; Boys, 4.6%.  Prevalence: self-harm ideation or acts (parent report) at age 12: Girls, 2.3%; Boys, 3.1%  Prevalence: self-harm ideation or acts (parent report) at age 15: Girls, 3.3%; Boys, 2.7%.  Girls significantly more likely to experience self-harm acts **(*OR* 3.4 [1.7, 6.8], *p <* .001)** when child self-report. Sex not significant predictor of ideation, nor for acts in parents’ self-reports. |
| Undheim & Sund, 2013  YMHS | Baseline, *N =* 2,464  FU, *N =* 2,359  T1, *M =* 13.7 (0.58, 12.5 - 15.7 years)  T2, *M =* 14.9 (0.59, 13.7 - 17.0) | T1/T2 =, 50.8% / 50.4% female  Place of parents’ birth: Norway (93.4%), one parent outside Norway (3.9%), Eastern Europe or Third World country (2.7%)  Parental occupation (SES proxy): Professional leader (9.8%), Upper middle class (28.6%), Lower middle class (13.2%), primary industry (8.3%), manual worker (36.5%), missing (3.7%) | 1 year, 2 waves  Model 1: Depression at age 15 (T2), gender, age, SES, and being bullied and being aggressive toward others at age  14 (T1)  Model 2: as above, and additionally suicidal ideation at T1 | (Multivariable) Association between BV aged 14 and SI aged 15: **Unstandardised b coefficient 0.38 (*SE* = 0.10); Standardised ß 0.07, T value = 4.1, *p <* .001, Part^2^ = 0.5%.** This was no longer significant once suicidal ideation at age 14 (T1) was also controlled for (data NR).  Prevalence (exposure): 10.03%  Prevalence (outcome): 13.4% - 32.5% across 5 items constituting the suicidal ideation scale. | **Among bullied adolescents, girls showed significantly higher scores of suicidal ideation than boys at Time 2 (*p <* .05).** |
| Winsper et al., 2012  ALSPAC | *N =* 6,043 (analyses based on 4,404 – 5,778)  *M =* 11.7 (10.4 – 13.6) | 51% female | 7 years, multiple waves:  Child: 3 waves at age 8, 10 and 11 years old; Mothers: 2 waves at child age 4, 7 and 9; Teachers: 2 waves at child age 7 and 9.  Model 1: Controlling for age and gender.  Model 2: Controlling for age, gender, and additionally abuse, domestic violence, and maladaptive parenting.  Model 3: Controlling for negative emotionality and conduct disorder in addition to age, gender, abuse, domestic violence, and maladaptive parenting. | 1. BV (victims only) age 8 and suicidal ideation (child report)  **Model A *aOR* 1.70, 95% *CI* [1.26, 2.29]**  **Model B *aOR* 1.70 [1.25, 2.31]**  **Model C *aOR* 1.57 [1.15, 2.16]**  2. BV (bully-victims) age 8 and suicidal ideation (child report)  **Model A *aOR* 3.50, 95% *CI* [2.34, 5.25]**  **Model B *aOR* 3.41 (2.24, 5.18)**  **Model C *aOR* 2.84 (1.81, 4.45)**  3. BV (victims only) age 10 and suicidal ideation (child report) **Model A *aOR* 2.40 (1.80, 3.19)**  **Model B *aOR* 2.20 (1.64, 2.96)**  **Model C *aOR* 1.95 (1.42, 2.66)**  4. BV (bully-victims) age 10 and suicidal ideation (child report)  **Model A *aOR* 4.23 (2.88, 6.20)**  **Model B *aOR* 3.84 (2.57, 5.74)**  **Model C *aOR* 3.20 (2.07, 4.95)**  5. Overt BV and suicidal ideation  **Model A *aOR* 2.30 (1.79, 2.96)**  **Model B *aOR* 2.19 (1.69, 2.84)**  **Model C *aOR* 1.88 (1.43*,* 2.47)**  6. Relational BV and suicidal ideation  **Model A *aOR* 1.76 (1.34*,* 2.32)**  **Model B *aOR* 1.74 (1.31*,* 2.30)**  **Model C *aOR* 1.60 (1.18*,* 2.16)**  7. BV (victims only) age 8 and suicidal/self-injurious behaviour (SIB) (child report)  **Model A *aOR* 2.36 (1.75*,* 3.18)**  **Model B *aOR* 2.28 (1.67*,* 3.09)**  **Model C *aOR* 2.05 (1.48*,* 2.83)**  8. BV (bully-victims) age 8 and SIB (child report)  **Model A *aOR* 2.92 (1.88*,* 4.53)**  **Model B *aOR* 2.60 (1.64*,* 4.13)**  **Model C *aOR* 2.67 (1.66*,* 4.29)**  9. BV (victims only) age 10 and SIB (child report)  **Model A *aOR* 2.53 (1.89*,* 3.38)**  **Model B *aOR* 2.45 (1.81*,* 3.32)**  **Model C *aOR* 2.25 (1.63*,* 3.09)**  10. BV (bully-victims) age 10 and SIB (child report)  **Model A *aOR* 3.87 (2.63*,* 5.69)**  **Model B *aOR* 4.07 (2.74*,*6.03)**  **Model C *aOR* 3.34 (2.17*,* 5.15)**  11. Overt BV and SIB  **Model A *aOR* 2.90 (2.22*,* 3.79)**  **Model B *aOR* 2.87 (2.17*,* 3.79)**  **Model C *aOR* 2.56 (1.91*,* 3.44)**  12. Relational BV and SIB  **Model A *aOR* 1.92 (1.46*,* 2.53)**  **Model B *aOR* 1.88 (1.41*,* 2.50)**  **Model C *aOR* 1.77 (1.31*,* 2.41)**  Prevalence (exposure): Child report (8 years): Victim (32.4%), bully-victim (6.8%); Child report (10 years): Victim (18.6%), bully-victim (5.4%); Overt victimisation (40.6%); Relational victimisation (19.6%)  Prevalence (outcome): Suicidal ideation (4.8%); Suicidal or self-injurious behaviour (4.6%) | Prevalence: Victim only, child report at 8 years (boys: 32.5%, girls: 32.4%, *OR* 1.10, 95% *CI* [0.98, 1.25])  **Bully-victim (boys: 9.3%, girls: 4.5%, *OR* 2.30, [1.82, 2.90])**  Victim only, child report at 10 years (boys 18.8%, girls: 18.4% *OR* 1.12, [0.97, 1.28])  **Bully-victim at 10 years (boys: 8.2%, Girls: 2.8% *OR* 3.20 [2.45, 4.14])**  **Overt bullying (boys: 46.3%, girls: 35.2%, *OR* 1.59 [1.43, 1.77])**  Relational bullying (boys: 17.9%, girls: 21.2%, *OR* 0.81, 95% *CI* [0.71, 0.92])  Suicidal ideation (boys: 5.2%, girls: 4.4%, *OR* 1.19 [0.94, 1.50])  **Suicidal or self-injurious behaviours (boys: 6.4%, girls: 90, 2.9%, *OR* 2.29 [1.77, 2.96]).** |
| Wu et al., 2021 | Baseline*, N =* 813  FU, *N =* 524  T1, *M =* 13.15 years (1.10, 11-16) | 43% female  High SES | 1 year, 2 waves  Age and gender | 1. Model A: Association between BV and NSSI, where bully-victims were distinguished from pure bullies and pure victims.  a. Bully-victims ***aOR* = 2.76, b = 1.02, *SE* = 0.26, *p =* 0.008**  b. Victims only *aOR* = 1.26, b = 0.23, *SE* = 0.30, *p =* ns  2. Model C: Association between BV and NSSI, ***aOR* 1.68, b = 0.52, *SE* = 0.22, *p =* 0.02**  Prevalence: Not reported | Not reported – NB has results of gender in a multivariate model (just not looking at association between BV and NSSI) |
| Zhu et al., 2021 | Baseline, *N =* 1,987  FU (Wave 2), *N =* 1,818  FU (Wave 3), *N =* 1,820  T1, *M =* 12.32 (0.53, 10 to 14)  T3, *M =* 13.33 (0.53) | 43.90% female  The average monthly income in 71.7% of recruited families exceeded RMB3000 (almost equal to 447 US dollars) | 6 months, 3 waves  Adolescent gender, age, childhood trauma. Other covariates were anxiety symptoms and NSSI at baseline | 1. Association between CV at T1 and NSSI at T3 **(ß = .21, *p <* .05)** when gender, age, childhood trauma and NSSI at T1 were included as covariates  2. Anxiety symptoms at T2 had a significant and positive indirect effect in the association between CV at T1 and NSSI at T3 (indirect effect, **ß** = .**04, *p <* .05, 95% *CI* [.014*,* .083**]): CV at T1 positively predicted anxiety symptoms at T2 (**ß = .22, *p <* .001**), which was positively associated with NSSI at T3 (**ß = .19, *p <* .001**)  Prevalence (exposure): *M =* 0.17 (SD 0.38)  Prevalence (outcome): NSSI in the last six months (T1: 15.0%, *n* = 291; T3: 15.6%, *n* = 272) | Not reported |

Note. BV = bullying victimisation; CV = cyberbullying victimisation; NSSI = nonsuicidal self-injury; SH = self-harm; SI = suicidal ideation; SA = suicide attempt; *OR* = Odds Ratio; *aOR* = adjusted Odds Ratio; *RR* = relative risk; *CI* = confidence interval; SES = socioeconomic status; NR = not reported; ns = not significant
