# Supplementary material for: The longitudinal course of childhood bullying victimization and associations with self‐injurious thoughts and behaviors in children and young people: A systematic review of the literature
Source: J Adolesc. 2022 Oct 9;95(1):5–33. doi: 10.1002/jad.12097 (PMC10092090; doi:10.1002/jad.12097)
Supplement: Supplementary file 5 — Supporting information. [file JAD-95-5-s005.docx]

Supplementary file 5. Definition characteristics and components of bullying within the measures

| Source | **Definition of bullying victimisation given to participants** | | **Components measured (within the items/at the analysis stage rather than the definitions provided to the participants)** | | |
| --- | --- | --- | --- | --- | --- |
|  | Definition used | Components of definition | Power imbalance | Intent to cause harm | Repetition |
| Bannink et al., 2014 | Unknown | N/A | Unknown | Unknown | Yes |
| Benatov et al., 2021 | TBV: Yes  CBV: Yes | Intention to harm | TBV: No  CBV: No | TBV: Yes  CBV: Yes | TBV: Yes  CBV: Yes |
| Blasco et al., 2019 | Yes | Power imbalance  Intent to cause harm  Repetition | Yes | Yes | Yes ^a^ |
| Borschmann et al., 2020 | No | N/A | No | Yes | Yes |
| Brunstein Klomek et al., 2019 | No | N/A | No | Yes | Yes |
| Cho, 2019 | No | N/A | No | Yes | Yes ^a^ |
| Cho and Glassner, 2019 | No | N/A | No | Yes | Yes ^a^ |
| Copeland et al., 2013 | Yes | Intention to harm | No | Yes | Yes ^a^ |
| Fisher et al., 2012 | Yes | Power imbalance  Intent to cause harm  Repetition | Yes | Yes | Yes |
| Garisch and Wilson, 2016 | Yes | Power imbalance  Intent to cause harm  Repetition | Yes | Yes | Yes ^a^ |
| Geoffroy et al., 2021 | No | N/A | No | No | Yes |
| Heikkilä et al., 2013 | Yes | Power imbalance  Intent to cause harm  Repetition | Yes | Yes | Yes |
| Hemphill et al., 2015 | No | N/A | No | Yes | Yes |
| Kiekens et al., 2019 | Yes | Power imbalance  Intent to cause harm  Repetition | Yes | Yes | Yes ^a^ |
| Kim et al., 2009 | Unknown | N/A | No | Yes | Yes |
| Klomek et al., 2008 | Unknown | N/A | Unknown | Unknown | Yes |
| Klomek et al., 2009 | Unknown | N/A | Unknown | Unknown | Yes |
| Le et al., 2017 | Yes | Power imbalance  Intent to cause harm  Repetition | Yes | Yes | Yes ^a^ |
| Le et al., 2019 | Yes | Power imbalance  Intent to cause harm  Repetition | Yes | Yes | Yes |
| Lereya et al., 2013 | No ^a^ | N/A | No | Yes | Yes ^a^ |
| Lereya et al., 2015 | ALSPAC: No ^a^  GSMS: Yes | ALSPAC: N/A  GSMS: Intention to harm | ALSPAC: No  GSMS: No | ALSPAC: Yes  GSMS: Yes | ALSPAC: Yes ^a^  GSMS: Yes ^a^ |
| Lung et al., 2020 | Unknown | N/A | No | No | No |
| Mars et al., 2020 | No | N/A | No | No | No |
| Mortier et al., 2017 | Yes | Power imbalance  Intent to cause harm  Repetition | Yes | Yes | Yes ^a^ |
| O’Connor et al., 2009 | No | N/A | No | No | No |
| Özdemir and Stattin, 2011 | No | N/A | No | Yes | Yes ^a^ |
| Perret et al., 2020 | TBV: No  CBV: Yes | TBV: N/A  CBV: Intention to harm | TBV: No  CBV: No | TBV: Yes  CBV: Yes | TBV: Yes  CBV: Yes ^a^ |
| Quintana-Orts et al., 2022 | No | N/A | No | Yes | Yes ^a^ |
| Sigurdson et al., 2018 | No | N/A | No | Yes | Yes |
| Silberg et al., 2016 | Yes | Intention to harm | No | Yes | Yes ^a^ |
| Sourander et al., 2006 | No | N/A | Unknown | Unknown | Yes |
| Undheim and Sund, 2013 | No | N/A | No | Yes | Yes |
| Winsper et al., 2012 | No ^a^ | N/A | No | Yes | Yes ^a^ |
| Wu et al., 2021 | No | N/A | No | Yes | Yes ^a^ |
| Zhu et al., 2021 | Unknown | N/A | No | Yes | Yes ^a^ |

Note. Yes ^a^ = Yes, but possibly not as strict a minimum cutoff as threshold in the literature (i.e., two or three times a month or more; Solberg & Olweus [2003]); No ^a^ = No definition of bullying is provided but examples are given during the interview

References

Solberg, M. E., & Olweus, D. (2003). Prevalence estimation of school bullying with the Olweus Bully/Victim Questionnaire. *Aggressive Behavior: Official Journal of the International Society for Research on Aggression*, *29*(3), 239-268.
