# Supplementary material for: The longitudinal course of childhood bullying victimization and associations with self‐injurious thoughts and behaviors in children and young people: A systematic review of the literature
Source: J Adolesc. 2022 Oct 9;95(1):5–33. doi: 10.1002/jad.12097 (PMC10092090; doi:10.1002/jad.12097)
Supplement: Supplementary file 6 — Supporting information. [file JAD-95-5-s008.docx]

Supplementary file. Quality assessment using Newcastle-Ottawa scale (2014)

| Source | Selection of cohorts | | | | Comparability | Assessment of outcome(s) | | | Overall score |
| --- | --- | --- | --- | --- | --- | --- | --- | --- | --- |
|  | Representativeness of exposed cohort | Non-exposed cohort selection | Ascertainment of exposure to bullying | Evidence of outcome not present at start | Confounders adjusted for | Assessment of outcome (self-harm, suicidality) | Length of follow-up | Retention good, information about attrition |  |
| Bannink et al., 2014 | * | * | - | * | ** | - | * | * | 7 |
| Benatov et al., 2021 | 0.5 | * | * | * | * | * | * | * | 7.5 |
| Blasco et al., 2019 | 0.5 | * | * | * | ** | * | * | * | 8.5 |
| Borschmann et al., 2020 | * | * | 0.5 | - | ** | - | * | * | 6.5 |
| Brunstein Klomek et al., 2019 | * | * | * | - | ** | * | * | * | 8 |
| Cho, 2019 | * | * | 0.5 | - | * | * | * | - | 5.5 |
| Cho and Glassner, 2019 | * | * | 0.5 | - | * | * | * | - | 5.5 |
| Copeland et al., 2013 | * | * | * | - | * | * | * | * | 7 |
| Fisher et al., 2012 | * | * | * | - | * | * | * | * | 7 |
| Garisch and Wilson, 2016 | 0.5 | * | 0.5 | - | * | * | - | - | 4 |
| Geoffroy et al., 2021 | * | * | - | - | ** | - | * | * | 6 |
| Heikkilä et al., 2013 | * | * | * | - | ** | * | * | * | 8 |
| Hemphill et al., 2015 | * | * | 0.5 | - | * | - | * | * | 5.5 |
| Kiekens et al., 2019 | * | * | * | - | * | * | * | * | 7 |
| Kim et al., 2009 | - | * | * | * | * | 0.5 | * | * | 6.5 |
| Klomek et al., 2008 | 0.5 | * | - | - | * | * | * | * | 5.5 |
| Klomek et al., 2009 | * | * | - | - | * | * | * | * | 6 |
| Le et al., 2017 | - | * | * | * | * | 0.5 | * | * | 6.5 |
| Le et al., 2019 | - | * | * | * | ** | 0.5 | * | * | 7.5 |
| Lereya et al., 2013 | * | * | * | - | * | - | * | * | 6 |
| Lereya et al., 2015 | * | * | * | - | * | * | * | * | 7 |
| Lung et al., 2020 | * | * | - | - | - | - | * | * | 4 |
| Mars et al., 2020 | * | * | - | - | * | - | * | * | 5 |
| Mortier et al., 2017 | - | * | * | * | ** | * | * | * | 8 |
| O’Connor et al., 2009 | - | * | - | - | - | * | * | - | 3 |
| Özdemir and Stattin, 2011 | * | * | - | - | * | * | * | * | 6 |
| Perret et al., 2020 | * | * | - | * | * | - | * | * | 6 |
| Quintana-Orts et al., 2022 | - | * | * | - | ** | * | - | * | 6 |
| Sigurdson et al., 2018 | * | * | - | - | * | * | * | * | 6 |
| Silberg et al., 2016 | * | * | * | - | - | * | * | * | 6 |
| Sourander et al., 2006 | * | * | - | * | ** | - | * | * | 7 |
| Undheim and Sund, 2013 | * | * | - | * | ** | * | * | * | 8 |
| Winsper et al., 2012 | * | * | * | - | ** | * | * | - | 7 |
| Wu et al., 2021 | - | * | * | - | * | - | * | * | 5 |
| Zhu et al., 2021 | * | * | * | * | ** | - | * | * | 8 |

* = Study received a Newcastle-Ottawa Scale star; - = Study not received a Newcastle-Ottawa Scale star

Reference

Wells, G., Shea, B., O’connell, D., Peterson, J., Welch, V., Losos, M., & Tugwell, P. (2014). The Newcastle-Ottawa Scale (NOS) for assessing the quality of nonrandomised studies in meta-analyses
