# Supplementary material for: The longitudinal course of childhood bullying victimization and associations with self‐injurious thoughts and behaviors in children and young people: A systematic review of the literature
Source: J Adolesc. 2022 Oct 9;95(1):5–33. doi: 10.1002/jad.12097 (PMC10092090; doi:10.1002/jad.12097)
Supplement: Supplementary file 7 — Supporting information. [file JAD-95-5-s004.docx]

Supplementary file. In-depth summaries by outcome.

## 1 Association between traditional bullying only and SITB

### 1.1 NSSI

Only one study (Wu et al., 2021) assessed NSSI as an outcome, finding a medium association, OR 1.68, (95% CI not reported, *p* = 0.02). When distinguishing bully-victims from victims only, there was a large association between bully-victims (OR 2.76, 95% CI not reported, *p* = 0.08) and NSSI but not for victims only (OR 1.26, 95% CI not reported, p > .05).

### 1.2 Self-harm

Six studies measured self-harm under 18 years, with one study looking at this outcome during early adolescence (age 12; Fisher et al., 2012) and four in mid-late adolescence (Hemphill et al., 2015; Lereya et al., 2013; Özdemir & Stattin, 2011; Sourander et al., 2006). One study measured self-harm in both mid-adolescence and again in young adulthood (Sigurdson et al., 2018). Quality assessment scores of the seven papers ranged from 5.5 (medium) to 7 (high), of which three are rated high quality (Fisher et al., 2012; Özdemir & Stattin, 2011; Sourander et al., 2006). All studies found some associations between traditional bullying and self-harm, although in one study this association was evident for self-harm ideation but not acts (Sourander et al., 2006). In a UK-based study (Lereya et al., 2013), the risks were largest in teacher-reported accounts that a child was a victim of bullying at both 7 and 10 years (OR 4.75, 95% CI [1.72, 13.07]), as compared to mother (OR 1.59 [1.19, 2.13]) or child reports (OR 1.66 [1.20, 2.31]). In one study of 15 to 17 years olds (Hemphill et al., 2015), fully-adjusted models that assessed traditional and cyberbullying as separate constructs found larger associations between traditional bullying and self-harm, particularly for bully-victims, up to aOR 2.40, 95% CI [1.34, 4.29], *p* < .01. Across all studies, associations between bullying and self-harm were generally larger during mid-adolescence (aORs 3.30, 95% CI [2.07, 5.26] for females to 4.62 [2.47, 8.67], *p* values < .001 for males; Sigurdson et al., 2018) compared to young adulthood (aORs 1.91, 95% CI [1.02, 3.58] in Hemphill et al., 2015, to 3.86 [1.31, 11.41], *p* values ranging between .014 – .047; Sigurdson et al., 2018).

### 1.3 Suicidal ideation

Fourteen studies from 12 different samples across Europe, Asia and North America looked at the association between traditional bullying and suicidal ideation. The quality assessment scores ranged from 5.5 (medium) to 8.5 (high), with 8 studies of high quality (Bannink et al., 2014; Benatov et al., 2021; Blasco et al., 2019; Brunstein Klomek et al., 2019; Heikkilä et al., 2013; Mortier et al., 2017; Undheim & Sund, 2013; Winsper et al., 2012). Seven studies assessing suicidal ideation under 18 years found some associations with the exposure, suggesting the odds of suicidal ideation is 1.56, 95% CI [1.21, 2.02] to 6.39, 95% CI [1.58, 25.88] times the odds as compared to non-victimised adolescents (Bannink et al., 2014; Brunstein Klomek et al., 2019; Cho, 2019; Heikkilä et al., 2013; Kim et al., 2009; Sigurdson et al., 2018; Winsper et al., 2012). In a study of 10 European countries, physical bullying in mid-adolescence (Time 2) increased the odds of suicidal ideation (at Time 3) by aOR 2.54 (95% CIs unreported, p < .05; see Section 1.6 for results by other sub-types). Three studies reported associations for bully-victims, with some large effects for suicidal ideation in the past two weeks in boys (aOR 6.39, 95% CI [1.58, 25.88]; Kim et al., 2009). These effects were also found after controlling for several demographics and childhood adversities among pre-adolescents (aOR 2.84, 95% CI [1.81, 4.45] aged 8, aOR 3.20 [2.07, 4.95] aged 10; Winsper et al., 2012). In the third study (Heikkilä et al., 2013), the associations dropped from aOR 5.4, 95% CI [1.5, 20.0], *p* = .01, adjusting for sex and age, to aOR 1.9 [0.4, 10.7], *p* = 0.05, after additionally adjusting for baseline depression and externalising symptoms. Among the studies looking at suicidal ideation aged 18+, associations were found for females (aOR 2.68, 95% CI [1.52, 4.73], p < .001; Sigurdson et al., 2018), in twin studies (ORs 1.9, 95% CI [1.3, 3.0] – 2.9 [1.2, 7.2], p <.01; Silberg et al., 2016), and for college students reporting first onset of suicidal ideation at Time 2 (aOR 3.2, 95% CI [1.08, 9.53], p = .04; Blasco et al., 2019). Only 4 studies, all with small effect sizes (aORs 1.04, 95% CI [0.67, 1.60] to 1.26, [0.83, 1.93]), did not find any statistically significant direct associations; two assessed the outcome in young adulthood (i.e., 18+; Klomek et al., 2008; Mortier et al., 2017), one looked at a sample of at-risk youths in Israeli vocational schools (Benatov et al., 2021), and the other only found an indirect effect via negative emotions (ß = .089, p < .01; Cho & Glassner, 2020). One study no longer found an association once controlling for baseline suicidal ideation (Undheim & Sund, 2013).

### 1.4 Suicide attempt

Three studies looked at the association with suicide attempt in mid-adolescence (Benatov et al., 2021; Brunstein Klomek et al., 2019; Sigurdson et al., 2018), with one study following their cohort into early adulthood (Sigurdson et al., 2018). One study looked at whether suicide attempts were adolescent-limited or persisted into their early 20s (Geoffroy et al., 2021). Quality assessment ratings ranged from 6 (medium) to 8 (high), with two rated high (Benatov et al., 2021; Brunstein Klomek et al., 2019). Multivariable models found small, weak associations (aORs 1.15, 95% CI [0.63, 2.09] to 1.58 [0.88, 2.81]) in mid-adolescence for Israeli students excluded from mainstream education (Benatov et al., 2021), but larger associations in a Norwegian sample that adjusted for gender and parental SES (aORs 3.90, 95% CI [2.26, 6.73] to 6.26 [2.94, 13.30], p values < .001; Sigurdson et al., 2018). Large associations across 10 European countries were also found for victims of physical bullying (aOR 4.72 [95% CI not reported], p < .001), both sporadic (i.e., measured at one time point) and chronic (i.e., measured at two time points) where a dose response was found (Brunstein Klomek et al., 2019). The impact of childhood bullying on suicide attempts in early adulthood approached statistical significance with a medium effect in one study (RR 2.53, 95% CI [0.93, 6.93], p = .071; Geoffroy et al., 2021), whilst the effect was large for young men in another (aOR 6.06, 95% CI [2.25, 16.36], p < .001; Sigurdson et al., 2018).

### 1.5 Other

Two studies combined measures of suicidality (i.e., suicide attempt/death; suicidal ideation/attempt) in their analyses (Klomek et al., 2009; Perret et al., 2020), one study measured the association with making a suicide plan (Mortier et al., 2017) and 4 studies used an aggregated measure of suicidality in their study design (Copeland et al., 2013; Kim et al., 2009; Mortier et al., 2017; Winsper et al., 2012). The quality assessment scores ranged from 6 (medium) to 8 (high), with 3 studies of high quality (Copeland et al., 2013; Mortier et al., 2017; Winsper et al., 2012). A UK-based birth cohort study (Winsper et al., 2012) found medium to large associations between bullying at the ages of 8 and 10 and suicidal behaviours aged 11 (aORs 1.77, [95% CI [1.31, – 2.41] to 3.34 [95% CI 2.17, – 5.15]) while a Korean sample of 1,666 adolescents aged 14 had inconsistent results (aORs 0.53, 95% CI [0.04, 6.75] to 4.94 [0.86, 28.33], p > .05; Kim et al., 2009), using a partially validated scale to measure the outcome. Medium associations with suicide ideation/attempt were found during mid-adolescence in a Canadian sample of youths (Perret et al., 2020), although the measures in this study were unvalidated; after adjusting for a variety of socioeconomic and family influences and baseline suicidal history, the odds of suicidal ideation/attempt two years after being victims of bullying at age 13 and 15 were 2.45 and 2.06 times the odds as compared to non-victims. In two studies of higher quality assessment, associations were smaller and weaker in older adolescents or young adults (Copeland et al., 2013; Mortier et al., 2017), except for male bully-victims where the odds of suicidality was 18.5 times the odds for non-bully-victims in their early 20s, although CIs were wide ([6.2, 55.1]; Copeland et al., 2013).

### 1.6 Associations by traditional bullying sub-types

Only 2 studies (Brunstein Klomek et al., 2019; Winsper et al., 2012), both rated of high quality, looked at associations between bullying and SITB by sub-type. In a study of 10 European countries (Brunstein Klomek et al., 2019), physical bullying but not verbal nor relational bullying victimisation had a medium association with suicidal ideation in mid to late adolescence (aOR 2.54 [95% CI not reported], *p* <.05) and a large association with suicide attempts (aOR 4.72 [95% CI not reported], *p* < .001). A dose response was found, where chronic relational and physical bullying (i.e., reported at Times 1 and 2) were associated with suicidal ideation at Time 3 compared to non-victimised youths (relational: aOR 2.63 [95% CI not reported], *p* < .05); verbal: aOR 2.08 95% CI nr], *p* > .05, nr). This was not found for chronic verbal bullying (aOR 0.58 (95% CI nr), *p* > .05, nr). Chronic physical bullying had a very large association with suicide attempts (aOR 7.69 [95% CI nr], *p* < .01) but not this did not exist for chronic verbal or relational bullying (aORs 0.51 to 1.10, 95% CIs and *p*-values nr). In a UK-based birth cohort study (ALSPAC; Winsper et al., 2012), victims of overt and relational bullying were more likely to experience suicidal ideation (aORs 1.88, 95% CI [1.43, 2.47] and 1.60 95% CI [1.18, 2.16]), and self-injurious behaviours (aORs 2.56 95% CI [1.91, 3.44] and 1.77 95% CI [1.31, 2.41]) compared to non-victims at 11 years old.

## 2 Association between cyberbullying only and SITB

### 2.1 NSSI

In a structural equation model with covariates that included baseline NSSI, one study in China (quality assessment: high) found a small, weak association between cyberbullying at Time 1 and NSSI measured six months’ later at Time 3 (ß = .21, p < .05; Zhu et al., 2021). This study also found a small, weak indirect effect of anxiety symptoms at T2 in the association between cyberbullying and NSSI (ß = .04, *p* < .05).

### 2.2 Self-harm

In an Australian sample of over 690 participants, cyberbullying victims were associated with greater odds of self-harm in mid-adolescence (OR 95% CI [1.51, 6.81]) but lost statistical significance after controlling for established risk factors including traditional bullying victimisation (aORs 0.87, 95% CI [0.36, 2.11] to 2.01 [0.82, 4.92]; Hemphill et al., 2015). In the only study looking at the effect of cyberbullying on self-harm in early adulthood, the largest associations were found for females, who were OR 2.42, 95% CI [1.41, 4.15]) at risk of self-harm compared to non-victims after adjusting for socioeconomic position (SEP), hours online and previous mental health problems (Mars et al., 2020). Males were OR 1.59, [0.35, 7.26], although CIs were wide. Both studies scored a medium quality assessment.

### 2.3 Suicidal ideation and suicide attempt

Two studies (Bannink et al., 2014; Benatov et al., 2021), of high quality assessment, found medium associations between cyberbullying and suicidal ideation in mid-adolescence (aORs 1.74, 95% CI [1.17, 2.61] to 2.13, 95% CI [1.27, 3.57]), but in Bannink et al. (2014) this association was not present in their Dutch sample of 14 years olds when controlling for baseline suicidal ideation. Despite finding an association with suicidal ideation, Benatov et al. (2021) did not find these associations with suicide attempts in their sample of Israeli adolescents. One study (Perret et al., 2020) looked at the association with suicidal ideation and attempts (combined) due to low numbers reporting these outcomes, measured at 15 and 17 years. In models only adjusted for sex, ORs ranged from 3.02 aged 15 (95% CI [2.28, 4.00]) to aOR 2.23 aged 17 [1.74, 2.86]. The associations at 15 and 17 years greatly reduced after controlling for baseline suicidal ideation and attempts in addition to several childhood variables, aORs 1.37 95% CI [0.97, 1.93] and 0.98 [0.73, 1.33], respectively.

## 3 Association between bullying (aggregate of traditional and cyberbullying) and SITB

### 3.1 NSSI

In a sample of Belgian college students (quality assessment 7 out of 9), bullying victimisation was associated with NSSI over a 12 month period in multivariable analyses (Kiekens et al., 2019). No differences were found for sporadic (1- 4 times) or repetitive (5 + times) NSSI, both with aORs of around 1.6. Conversely, in one sample of secondary school students in New Zealand, no significant associations were found between bullying and NSSI using cross-lagged panel correlational analysis across two waves, with a five month follow-up (Garisch & Wilson, 2015).

### 3.2 Self-harm

Associations were found between bullying and self-harm in pre-adolescent (Borschmann et al., 2020), early adolescent (Lung et al., 2020) and mid-adolescent samples (O'Connor et al., 2009), with some exceptions. Notably, the association was present among those with repeated self-harm (unadjusted OR 2.98, 95% CI [1.15, 7.71]) but not those reporting self-harm for the first time at Time 2, OR 1.06 [0.32, 3.49] (O'Connor et al., 2009). In a study with wide CIs (Borschmann et al., 2020), large associations were present in those reporting bullying at two waves (aOR 24.6, 95% CI [3.53, 150.55]) but this association was smaller and weaker at only one wave before the outcome, aOR 6.78, 95% CI [0.94, 49.07]. The quality assessment of these studies ranged from 3 (low) to 6.5 (medium), using mostly unvalidated measures.

### 3.3 Suicidal ideation

Two studies of medium-high quality assessment followed the same survey design at the same schools in Vietnam, but among two different ages groups (11 – 16 vs. 12 – 17), reporting results for 1) victims only and 2) bully-victims. Adjusted results found that victims at Time 1 were 2.02 to 2.5 times more likely to have suicidal ideation compared to non-involved students. In Le et al. (2017), victims at both time points had an increased risk of 3.1. Among bully-victims, a medium association was found in one study (aOR 1.83, p < .05; Le et al., 2019), but in the slightly younger sample the association was only present when bullying victimisation occurred at two time points (aOR 3.8, p < .01; Le et al., 2017).

## 4 Influence of sex/gender on the association between bullying and SITB

Sex and/or gender was often included in multivariable models as a control variable and many studies provided prevalence rates of bullying and/or SITB by sex/gender. The quality assessment of these studies ranged from 5 to 7.5 (out of 9), with four studies of high-quality assessment (Bannink et al., 2014; Copeland et al., 2013; Fisher et al., 2012; Le et al., 2019).

### 4.1 Prevalence rates (bullying)

Across three studies (Bannink et al., 2014; Mars et al., 2020; Perret et al., 2020), prevalence rates for cyberbullying were slightly higher in girls than boys at age 13, 15 and 18 but not 12 and 17 years old. Only two studies (Sigurdson et al., 2018; Winsper et al., 2012) presented rates of bullying by gender and sub-type (e.g., overt vs. relational). In a UK study (Winsper et al., 2012), girls aged 8 – 10 were more likely to report relational bullying than boys (21.2% vs. 17.9%, respectively, OR 0.81, 95% CI [0.71, 0.92]) and boys were more likely to report overt bullying than girls (46.3% vs. 35.2%, respectively, OR 1.59 [1.43, 1.77]), although in a sample of 13 year olds the distributions between the bullying sub-types (i.e., teasing, physical assault, exclusion) were similar (Sigurdson et al., 2018). Across other forms of bullying, prevalence rates for girls ranged from 3.7% (bullied frequently) to 36.1% (bullied sometimes) and from 9.4% (bullied frequently) to 47.8% (bullied sometimes) for boys (Bannink et al., 2014; Copeland et al., 2013; Klomek et al., 2009; Le et al., 2017; Le et al., 2019; Sigurdson et al., 2018; Winsper et al., 2012). In pre-adolescence, bully victimisation was higher for boys in two studies (Lereya et al., 2013; Winsper et al., 2012) using ALSPAC data (UK) and another with a Finnish sample (Brunstein Klomek et al., 2019). In mid-adolescence, the studies that reported on rates by gender found little differences apart from boys being more likely to be bully-victims (Copeland et al., 2013; Le et al., 2017; Winsper et al., 2012).

### 4.2 Prevalence rates (SITBs)

Few studies looked at prevalence of SITBs by sex/gender. In early adolescence, there were similar levels of prevalence for suicidal ideation and slightly higher rates of suicidal or self-injurious behaviours in boys (Fisher et al., 2012; Winsper et al., 2012). Suicidal ideation was higher in girls than boys in two samples during mid-adolescence (Bannink et al., 2014; Kim et al., 2009), including ideations over the past 6 months (41.59% vs. 24.38%, p < .001) and the past 2 weeks (50.55% vs. 35.33%, p <.001) but rates were the same between genders in a sample of Vietnamese high school students (Le et al., 2017; Le et al., 2019). Self-harm was higher for girls in studies in the UK (Lereya et al., 2013; Mars et al., 2020; O'Connor et al., 2009), Australia (Borschmann et al., 2020) and Norway (Sigurdson et al., 2018). No gender differences were found for lifetime NSSI or self-harm without suicidal intent for a study in 16-year-olds in New Zealand (Garisch & Wilson, 2015). In one study, suicide attempts were higher in young men than young women (O'Connor et al., 2009), but not in another study (Sigurdson et al., 2018). However, in this study, the risk difference over time between young adults previously bullied versus not bullied was larger among males (12.8%) than females (3.0%) for suicide attempts (Sigurdson et al., 2018).

### 4.3 Longitudinal associations between bullying and SITBs, by sex/gender

Of the 4 studies that included interactions terms between sex/gender and bullying on SITBs in multivariable models, one reported non-statistically significant interactions without specifying the effect size (Perret et al., 2020), one did not report on the interaction terms (Sigurdson et al., 2018), and the other did not specify the interaction terms but stratified significant interactions by gender (Copeland et al., 2013). The final study reported small interactions for gender x traditional bullying (aOR 1.41, 95% CI [0.83, 2.33], *p* = .20) and gender x cyberbullying (aOR 1.39 [0.56, 3.45], *p* = .48) but did not stratify the results due to a non-statistically significant interaction (Bannink et al., 2014).

In the 7 studies that stratified results by gender, 5 presented results on the association with self-harm, 4 with suicidal ideation, and 4 with other suicidal behaviours.

#### 4.3.1 Associations between bullying and self-harm

The association with self-harm by sex/gender was explored in one study of young adults (Mars et al., 2020) and two in pre-early adolescence and one which looked at mid-adolescence and young adults (Sigurdson et al., 2018). The association of cyberbullying with self-harm was stronger for young women (aOR 2.42, 95% CI [1.41, 4.15]) than young men (aOR 1.59 [0.35, 7.26]), while traditional bullying had a stronger association with self-harm for young men (aOR 3.86, 95% CI [1.31, 11.41], *p* = .014) than women (aOR 1.91 [1.01, 3.63], *p* = .047). The risk of self-harm aged 12 after being bullied in pre-adolescence was high for both boys and girls (Fisher et al., 2012), with the associations strongest for boys when bullying was reported by the mother (RR 4.92, 95% CI [2.33, 10.40]) and strongest for girls when reported by the children themselves (RR 4.16, [1.93, 8.95]).

#### 4.3.2 Associations between bullying and suicidal ideation

In adjusted models, girls who were victims of bullying were aORs 2.1 to 4.1 more likely to experience suicidal ideation compared to female non-victims, with strong associations at both early/mid-adolescence and young adulthood (Kim et al., 2009; Le et al., 2017; Le et al., 2019; Sigurdson et al., 2018). The pattern for boys was less clear, ranging from aOR 0.94 to 3.63 compared to male non-victims. The association with suicidal ideation for victims only (in childhood) remained modest in young women (aOR 2.68, 95% CI [1.52, 4.73], p < .001) and smaller in young men (aOR 1.76 [0.89, 3.49], p = 103; Sigurdson et al., 2018). For bully-victims in Kim et al. (2009), the association with ideation was high for boys (aOR 6.39, [1.58, 25.88] p < .01), and for girls (aOR 6.5, 95% CI [2.2, 19.5], *p* < .001).

#### 4.3.3 Associations between bullying and other suicidal behaviours

Finally, a strong effect for bullying on suicide attempts or death by suicide was found when bullying was frequent rather than occasional (Klomek et al., 2009), particularly in girls (aOR 6.3, 95% CI [1.5, 25.9]) compared to boys (aOR 3.8, 95% CI [0.99, 14.3]). Another Scandinavian study found high odds of suicide attempts in mid-adolescence for boys (aOR 6.26, 95% CI [2.94, 13.30]) and girls (aOR 3.90 [2.26, 6.73], bullied aged 13, that continued into young adulthood for these same young men (aOR 6.06 [2.25, 16.36]) but not the young women in the sample (aOR 1.30 [0.49, 3.45]; Sigurdson et al., 2018). For bully-victims in a US-based study (Copeland et al., 2013), the association with suicidality (i.e., self-harm, suicidal ideation and attempts) was even higher for young adult men (OR 18.5, 95% CI [6.2, 55.1], *p* < .001) compared to young women (OR 0.6 95% CI [0.1, 3.9], *p* = .56), although these unadjusted odds ratios did not control for other variables.
