# Supplementary material for: The longitudinal course of childhood bullying victimization and associations with self‐injurious thoughts and behaviors in children and young people: A systematic review of the literature
Source: J Adolesc. 2022 Oct 9;95(1):5–33. doi: 10.1002/jad.12097 (PMC10092090; doi:10.1002/jad.12097)
Supplement: Supplementary file 8 — Supporting information. [file JAD-95-5-s007.docx]

Supplementary file. Prevalence rates.

Table 1. Prevalence of bullying, cyberbullying and aggregated bullying/cyberbullying by gender

| Exposure | Author | Age (years) | Timeframe | Prevalence | | Statistical tests | Notes |
| --- | --- | --- | --- | --- | --- | --- | --- |
|  |  |  |  | Girls | Boys |  |  |
| Traditional bullying only | Bannink et al., 2014 | M = 12.50 (SD = 0.62) | 4 weeks | 17.9% | 19.6% | NR |  |
|  | Copeland et al., 2013 | 9 – 16 | 3 months | 23.4% | 28.8% | *p* = .15 | Additionally, more boys were bully-victims (27.6% girls vs. boys: 72.4%, *p* < .01) but similar numbers were victims only (52.9% girls vs. 47.8% boys, *p* = .34) |
|  | Fisher et al., 2012 | 7 – 12  7 – 12 | NR | 15.11%  10.28% | 17.87%  12.09% | NR | (Mothers’ reports)  (Child reports) |
|  | Klomek et al., 2009 | 8  8 | Past month  Past month | 3.7%  36.1% | 9.4%  47.8% | NR | (Frequent bullying victimisation)  (Sometimes bullying victimisation) |
|  | Sigurdson et al., 2018 | M = 13.7 (SD = 0.58) | 6 months | 10.0% | 9.7% | *p* = .784 |  |
|  | Winsper et al., 2012 | 8  8  10  10  8 – 10  8 – 10 | 6 months  6 months  6 months  6 months  6 months  6 months | 32.4%  4.5%  18.4%  2.8%  35.2%  21.2% | 32.5%  9.3%  18.8%  8.2%  46.3%  17.9% | OR 1.10, 95% CI [0.98, 1.25]  OR 2.30 [1.82, 2.90]  OR 1.12, [0.97, 1.28])  OR 3.20 [2.45, 4.14])  OR 1.59 [1.43, 1.77])  OR 0.81 [0.71, 0.92]) | (Victim only, child report)  (Bully-victims, child report)  (Victim only, child report)  (Bully-victims, child report)  (Overt bullying)  (Relational bullying) |
| Cyberbullying only | Bannink et al., 2014 | M = 12.50 (SD = 0.62) | 4 weeks | 3.2% | 2.0% | NR |  |
|  | Perret et al., 2020 | 12  13  15  17 | Since start of school year | 7.1%  11.6%  19.3%  8.3% | 6.4%  6.8%  10.7%  5.3% | *p* = .600  *p* = .005  *p* < .001  *p* = .044 |  |
| Aggregated bullying (traditional and cyber) | Bannink et al., 2014 | M = 12.50 (SD = 0.62) | 4 weeks | 2.4% | 2.8% | NR |  |
|  | Le et al., 2017 | M = 14.7 (SD = 1.9) | 6 months  6 months | 23.3%  27.0% | 24.9%  34.7% | NR  NR | (Victims only)  (Bully-victims) |
|  | Le et al., 2019 | M = 13.71 (SD = 1.89) | 6 months  6 months | 21.61%  8.18% | 31.78%  10.90% | NR  NR | (Victims only)  (Bully-victims) |

Note. M = Mean; SD = Standard deviation; OR = (unadjusted) Odds Ratio; NR = not reported

Table 2. Prevalence of SITBs by gender

| Outcome | Author | Age (years) | Timeframe | Prevalence | | Statistical tests | Notes |
| --- | --- | --- | --- | --- | --- | --- | --- |
|  |  |  |  | Girls | Boys |  |  |
| Self-harm | Borschmann et al., 2020 | 11 - 12 | Past 12 months | 3% | 2% | OR 3.2 [2.1, 5.0] |  |
|  | Fisher et al., 2012 | 7 – 12 | Past 6 months | 1.5% | 1.4% | NR |  |
|  | Lereya et al., 2013 | M = 16.7 (SD = 0.2) | Past 12 months | 13.13%  15.06% | 3.38%  3.74% | NR  NR |  |
|  | Mars et al., 2020 | M = 20.8 | Past 12 months | 13% | 10% | NR |  |
|  | O’Connor et al., 2009 | M = 15.2 (SD = 0.72) | Past 6 months | 4.0% | 2.2% | NR |  |
|  | Sigurdson et al., 2018 | M = 14.9 (SD = 0.59)  M = 27.2 (SD = 0.59) | Lifetime  Lifetime | 12.48%  14.24% | 5.37%  4.28% | NR  NR |  |
|  | Sourander et al., 2006 | 12  12  15  15 | Past 6 months  Past 6 months  Past 6 months  Past 6 months | 2.7%  2.3%  12.6%  3.3% | 3.1%  3.1%  4.6%  2.7% | NR  NR  NR  NR | (Child report)  (Parent report)  (Child report)  (Parent report)  Self-harm acts more likely in girls when child self-report (OR 3.4 [1.7, 6.8], p < .001) |
| Suicidal ideation | Bannink et al., 2014 | M = 14.31 (SD = 0.58) | Past 12 months | 16.3% | 7.5% | *p* < .001 |  |
|  | Kim et al., 2009 | 13 – 15 | Past 6 months  Past 2 weeks | 41.59%  50.55% | 24.38%  35.33% | *p* < .001  *p* < .001 |  |
|  | Le et al., 2017 | M = 14.7 (SD = 1.9) | Past 6 months | 13.4% | 12.2% | *p* > .05 |  |
|  | Le et al., 2019 | M = 13.71 (SD = 1.89) | Past 6 months | 13.43% | 12.15% | *p* > .05 |  |
|  | Sigurdson et al., 2018 | M = 14.9 (SD = 0.59)  M = 27.2 (SD = 0.59) | Past 2 weeks  Past 2 weeks | 32.55%  15.66% | 16.83%  19.83% |  |  |
|  | Winsper et al., 2012 | M = 11.7 (Range 10.4 – 13.6) | Past 2 years | 4.4% | 5.2% | OR 1.19 [0.94, 1.50] |  |
| Other | Kim et al., 2009 | 13 – 15 | Past 6 months | 13.4% | 9.5% | *p* < .001 | (Suicidal behaviours) |
|  | Winsper et al., 2012 | M = 11.7 (Range 10.4 – 13.6) | Past 2 years | 2.9% | 4.4% | OR 2.29 [1.77, 2.96] | (Suicidal or self-injurious behaviours) |

Note. M = Mean; SD = Standard deviation; OR = (unadjusted) Odds Ratio; NR = not reported
